# Supplementary material for: Women’s preference to apply shared decision-making in breast cancer screening: a discrete choice experiment
Source: BMJ Open. 2022 Nov 8;12(11):e064488. doi: 10.1136/bmjopen-2022-064488 (PMC9644356; doi:10.1136/bmjopen-2022-064488)
Supplement: Supplementary data [file bmjopen-2022-064488supp001.pdf]

"Women's Preferences for Breast Cancer Screening"

We appreciate your collaboration in the ProShare research project "*The collaboration of healthcare professionals to include Shared Decision Making in the breast cancer screening program*" (P18/00773), led by Dr. Misericòrdia Carles. This project is supported and funded by the Carlos III Institute of Health and co-financed by the European Regional Development Fund (ERDF) "A way of making Europe" y "European Union's Horizon 2020 research and innovation program under the Marie Skłodowska-Curie grant agreement" No. 713679 and from the Universitat Rovira i Virgili (URV).

The objective of your participation is to identify women's preferences and determine the cost-benefit of a new care model for the breast cancer screening program. So, we present you, in two hypothetical options and you must choose only one of them according to your preferences. Consider that there are no good or bad answers.

Your answers will be confidential and will only be used for research purposes. Answer the following questions truthfully, considering that your identity will never be exposed.

Shared Decision Making in Health

1. Socio-demographic characteristics
a. Name: __________

b. Age (years):______________

c. Birthplace (mark with an X)

	Spain
	Other country

d. Civil status (mark with an X)

	Single
	With partner

e. Education level (mark with an X)

	Primary
	Secondary
	University

f. Main occupation (mark with an X)

	Paid work
	Household chores
	Unemployed

g. Family history of breast cancer (mark with an X)

	Yes
	No
	Unknown

h. Do you have your mammogram regularly? (mark with an X)

	Yes
	No

2. Experimental design

The next 12 questions will be asked in consultation on three characteristics of the breast cancer screening program:

1. **How do I receive the information?** You will find two possibilities; in one of them you are informed about the benefits and risks of mammography through a **leaflet** or by **health professional**.

2. Dialogue about my preferences? You'll find two possibilities; in one, you **meet with a healthcare professional** to discuss your preferences, fears, or concerns about mammography. And on the other hand, **the health system** simply programs its mammography according to its age.

3. Who makes the decision? You will find three options about who makes the decision for attended -or no- to mammography: the **health professional**, **the woman** or **jointly**. Independent if later this is concrete the mammography

In each question you must choose the option you prefer. Each option includes the three features above.

For example:
Question 0: Mark with an "X" the option you prefer

Option A	Option B
I am informed by a **healthcare professional** about the benefits and risks, **I also express my fears**, beliefs, or preferences of mammography. Finally, **I myself made the decision** attend -or not- mammography.	I am informed by a **healthcare professional** about the benefits and risks, **I also express my fears**, beliefs or preferences of mammography. Finally, **we jointly made the decision** attend -or not- to mammography.

We are aware that in some questions the options may be very similar to you, yet there is always some different characteristics There are no correct or incorrect answers, you should select only based on your personal appreciation.

SURVEY

1: Mark with an "X" the option you prefer

Option A	Option B

**The healthcare professionals inform** me about the benefits and risks; **I also tell them about my fears**, beliefs, or preferences for mammography. Finally, **the healthcare professional makes the decision** attend -or not- to the mammography.	**The healthcare professionals inform me** about the benefits and risks; **I also tell them about my fears**, beliefs, or preferences for mammography. Finally, **I myself made the decision** attend -or not- to the mammography.

2.Mark with an "X" the option you prefer

Option A	Option B
I receive information about the benefits and risks through a **leaflet**; **I also share with a healthcare professional my fears** or beliefs about mammography. Finally, **we jointly made the decision** attend -or not- to the mammography.	**I am informed by a healthcare professional** about the benefits and risks; **I also express my fears, beliefs,** or preferences of mammography. Finally, **I myself made the decision** attend -or not- to the mammography.

3. Mark with an "X" the option you prefer

Option A	Option B
I receive information about the benefits and risks through **a leaflet**; **I also tell a healthcare professional my fears** or beliefs about mammography. Finally, **the professional makes the decision** attend -or not- to the mammography.	**The healthcare professionals inform** me about the benefits and risks; **I also tell them about my fears**, beliefs, or preferences for mammography. Finally, **I myself made the decision** attend -or not- to the mammography.

4. Mark with an "X" the option you prefer

Option A	Option B
**I am informed by a healthcare professional** about the benefits and risks, but **without the possibility of expressing my fears** or beliefs about mammography as it is defined by the health program. Finally, **I myself made the decision** attend -or not- to the mammography.	**The healthcare professional informs me** about the benefits and risks**; I also tell them about my fears**, beliefs, or preferences for mammography. Finally, **the healthcare professional makes the decision** attend -or not- to the mammography.

5. Mark with an "X" the option you prefer

Option A	Option B
I receive information about the benefits and risks through **a leaflet**; I also **tell a**	**The healthcare professional informs me** about the benefits and risks; **I also**

**healthcare professional my fears** or beliefs about mammography. Finally, **we jointly made the decision** attend -or not- to the mammography.	**tell them about my fears**, beliefs, or preferences for mammography. Finally, the **healthcare professional makes the decision** attend -or not- to the mammography.

6. Mark with an "X" the option you prefer

Option A	Option B
I receive information about the benefits and risks through **a leaflet**; **I also tell a healthcare professional my fears** or beliefs about mammography. Finally, **I myself made the decision** attend -or not- to the mammography.	**The healthcare professional informs me** about the benefits and risks; **I also tell them about my fears**, beliefs, or preferences for mammography. Finally, **the healthcare professional makes the decision** attend -or not- to the mammography.

7. Mark with an "X" the option you prefer

Option A	Option B
I receive information about the benefits and risks through **a leaflet**; **my mammography programs the health system**, so I do not manifest my fears, beliefs, or preferences about mammography to a health professional. Finally, **I myself made the decision** attend -or not- to the mammography.	**The healthcare professional informs me** about the benefits and risks; **I also tell them about my fears**, beliefs, or preferences for mammography. Finally, the **healthcare professional makes the decision** attend -or not- to the mammography.

8. Mark with an "X" the option you prefer

Option A	Option B
I receive information about the benefits and risks through **a leaflet**; **I also tell a healthcare professional my fears** or beliefs about mammography. Finally, **we jointly made the decision** attend -or not- to the mammography.	**I am informed by a health professional** about the benefits and risks; **my mammography programs the health system**, so I do not manifest my fears, beliefs, or preferences about mammography to a health professional. Finally, **I myself made the decision** attend -or not- to the mammography.

9. Mark with an "X" the option you prefer

Option A	Option B
I receive information about the benefits and risks in a **leaflet; I also share my fears** or beliefs about mammography with a health professional. Finally, **I myself**	**I am informed by a health professional** about the benefits and risks; my mammography programs the health system, **so I do not manifest my fears**, beliefs or preferences about

**made the decision** attend -or not- to the mammography.	mammography with a health professional. Finally, **I myself made the decision** attend -or not- to the mammography.

10. Mark with an "X" the option you prefer

Option A	Option B
I receive information about the benefits and risks in a **leaflet**; **I also share my fears** or beliefs about mammography with a health professional. Finally, **the professional makes the decision** attend -or not- to the mammography.	**I am informed by a health professional** about the benefits and risks; however, **I do not manifest my fears**, beliefs or preferences about mammography as it programs the health system. Finally, **I myself made the decision** attend -or not- to the mammography

11. Mark with an "X" the option you prefer

Option A	Option B
I receive information about the benefits and risks in a **leaflet**; **I also share my fears** or beliefs about mammography with a health professional. Finally, **the professional makes the decision** attend -or not- to the mammography	I receive information about the benefits and risks in a **leaflet**; **I also share my fear**s or beliefs about mammography with a health professional. Finally, **I myself made the decision** attend -or not- to the mammography

12. Mark with an "X" the option you prefer

Option A	Option B
I receive information about the benefits and risks a **leaflet**; **my mammography programs the health system**, so I do not manifest my fears, beliefs or preferences about mammography with a health professional. Finally, **I myself made the decision** attend -or not- to the mammography	I receive information about the benefits and risks in a **leaflet**; **I also share my fears** or beliefs about mammography with a health professional. Finally**, the professional makes the decision** attend -or not- to the mammography

3. Cost of health

The breast cancer screening is currently not paid to women, as it is covered by the public health service. Regardless of the current cost, if you had the option of attending an appointment with a healthcare professional to inform you about the benefits and risks of mammography, discuss with him/her your preferences and concerns and jointly make the decision to attend -or not to mammography **How much money would you be willing to pay?**

	0 euros

	10 euros
	20 euros
	30 euros
	40 euros
	60 euros or more

Thank you so much for the time you took to answer this survey to improve women's care in breast cancer screening.

ProShare
