# Supplementary material for: Women’s preference to apply shared decision-making in breast cancer screening: a discrete choice experiment
Source: BMJ Open. 2022 Nov 8;12(11):e064488. doi: 10.1136/bmjopen-2022-064488 (PMC9644356; doi:10.1136/bmjopen-2022-064488)
Supplement: Author's manuscript [file bmjopen-2022-064488.draft_revisions.pdf]

BMJ Open

BMJ Open is committed to open peer review. As part of this commitment we make the peer review history of every article we publish publicly available.

When an article is published we post the peer reviewers' comments and the authors' responses online. We also post the versions of the paper that were used during peer review. These are the versions that the peer review comments apply to.

The versions of the paper that follow are the versions that were submitted during the peer review process. They are not the versions of record or the final published versions. They should not be cited or distributed as the published version of this manuscript.

BMJ Open is an open access journal and the full, final, typeset and author-corrected version of record of the manuscript is available on our site with no access controls, subscription charges or pay-per-view fees (http://bmjopen.bmj.com).

If you have any questions on BMJ Open's open peer review process please email info.bmjopen@bmj.com

BMJ Open

Women’s Preference to Apply Shared Decision-Making in Breast Cancer Screening: A Discrete Choice Experiment

Journal:	*BMJ Open*
Manuscript ID	bmjopen-2022-064488
Article Type:	Original research
Date Submitted by the Author:	05-May-2022
Complete List of Authors:	Hernández Leal, María José; Universitat Rovira i Virgili, Economic ; Research Group on Statistics, Economic Evaluation and Health (GRAEES).<br>Pérez-Lacasta, María José; Universitat Rovira i Virgili, Economic ; Research Group on Statistics, Economic Evaluation and Health (GRAEES).<br>Cardona, Angels; Area Q: Evaluation and Research in the Field of Social Sciences and Health<br>Codern-Bové, Núria; Autonomous University of Barcelona, School of Nursing and Occupational Therapy (EUIT)<br>Vidal, Carmen; Catalan Institute of Oncology, Cancer Prevention and Control Program<br>Rue, Montserrat; University of Lleida, Basic Medical Sciences; Research Group on Statistics, Economic Evaluation and Health (GRAEES). ,<br>Forné, Carles; University of Lleida, Department of Basic Medical Sciences; Heorfy Consulting, HEOR freelance consultant<br>Carles-Lavila, Misericòrdia; Universitat Rovira i Virgili, Economics; Research Group on Statistics, Economic Evaluation and Health (GRAEES).
Keywords:	HEALTH ECONOMICS, Breast tumours < ONCOLOGY, PREVENTIVE MEDICINE, PRIMARY CARE

SCHOLARONE™
Manuscripts

I, the Submitting Author has the right to grant and does grant on behalf of all authors of the Work (as defined in the below author licence), an exclusive licence and/or a non-exclusive licence for contributions from authors who are: i) UK Crown employees; ii) where BMJ has agreed a CC-BY licence shall apply, and/or iii) in accordance with the terms applicable for US Federal Government officers or employees acting as part of their official duties; on a worldwide, perpetual, irrevocable, royalty-free basis to BMJ Publishing Group Ltd ("BMJ") its licensees and where the relevant Journal is co-owned by BMJ to the co-owners of the Journal, to publish the Work in this journal and any other BMJ products and to exploit all rights, as set out in our licence.

The Submitting Author accepts and understands that any supply made under these terms is made by BMJ to the Submitting Author unless you are acting as an employee on behalf of your employer or a postgraduate student of an affiliated institution which is paying any applicable article publishing charge ("APC") for Open Access articles. Where the Submitting Author wishes to make the Work available on an Open Access basis (and intends to pay the relevant APC), the terms of reuse of such Open Access shall be governed by a Creative Commons licence – details of these licences and which Creative Commons licence will apply to this Work are set out in our licence referred to above.

Other than as permitted in any relevant BMJ Author's Self Archiving Policies, I confirm this Work has not been accepted for publication elsewhere, is not being considered for publication elsewhere and does not duplicate material already published. I confirm all authors consent to publication of this Work and authorise the granting of this licence.

For peer review only

Original Research
BMJ Open

Women's Preference to Apply Shared Decision-Making in Breast Cancer Screening: A Discrete Choice Experiment

Running head: A DCE for SDM in breast cancer screening

María José Hernández-Leal[a,b,c] María José Pérez-Lacasta [a,c] Angels Cardona[d] Núria Codern-Bové[e] Carmen Vidal-Lancis[f] Montserrat Rué[c,g] Carles Forné[h,i], Misericòrdia Carles-Lavila [a,b,c] on behalf of the ProShare Group

a. Department of Economics, University of Rovira i Virgili. Reus, Spain.
b. Research Centre on Economics and Sustainability (ECO-SOS). Reus, Spain.
c. Research Group on Statistics, Economic Evaluation and Health (GRAEES). Reus, Spain.
d. Area Q: Evaluation and Research in the Field of Social Sciences and Health, Barcelona, Spain.
e. School of Nursing and Occupational Therapy (EUIT), Autonomous University of Barcelona. Terrassa, Spain.
f. Cancer Prevention and Control Program, Catalan Institute of Oncology-IDIBELL. L'Hospitalet de Llobregat, Spain
g. Department of Basic Medical Sciences, University of Lleida-IRB. Lleida, Spain.
h. Department of Basic Medical Sciences, University of Lleida, Spain.
i. HEOR freelance consultant, Heorfy Consulting. Reus, Spain.

ProShare Group: Misericòrdia Carles-Lavila, Núria Codern-Bové, María José Hernández-Leal, María José Pérez, Roger Pla, Vanesa Ramos, Carmen Vidal

Corresponding author: Misericòrdia Carles-Lavila, Department of Economic, University of Rovira i Virgili, Avenida Universitat 1, 43204. Reus, Spain. Email: misericordia.carles@urv.cat

For peer review only

ABSTRACT

Objective: To analyse women's stated preferences for establishing the relative importance of each attribute of Shared Decision-making (SDM) and their willingness to pay (WTP) for more participatory care in breast cancer (BC) screening.
Methods: A Discrete Choice Experiment was conducted in which women, aged between 50 and 60 years, of Catalonia were invited to participate. It was designed with 12 choice tasks comparing three attributes of usual care and SDM: 'Information talk', 'Dialogue talk', and 'Decision talk' to attend screening. The survey also included one question on WTP and seven socioeconomic questions. The analysis was performed using conditional mixed-effects logit regression and stratification according to WTP.
Results: Sixty-five women completed the survey. The only relevant attribute is 'Decision talk'. If they decided to attend screening alone, they would get 20% more utility (coefficient=2.879; 95%CI=2.297 , 3.461) than if they would do it jointly with the healthcare professional (coefficient=2.375; 95%CI=1.573 , 3.177). They were indifferent to the way of receiving the information, and dialog their beliefs with a professional to schedule the next screening. Fifty-two percent were willing to pay for SDM screenings.

Conclusions: **'**Decision talk'step was the dominant SDM attribute for women in BC screening. They prefer to make the decision by themselves -applying an Informed Decision-making- or with a healthcare professional using SDM.

Keywords: Shared Decision-making; Informed Decision-making; Breast cancer screening; Discrete Choice Experiments; Willingness-to-pay.

Strengths and limitations of this study

- Women reject a paternalistic model for breast cancer screening and prefer Informed Decision-making or Shared Decision-making.
- Decision talk step (who makes the decision to attend -or not- screening) is the only significant attribute.
- Women perceive a 20% more utility when making the decision alone than making it jointly with a health professional.
- The 52.3% were willingness-to-pay for a SDM in BC screening.
- There is no difference in the responses of women who are and are not willing-to-pay.

For peer review only

1. BACKGROUND

Breast cancer (BC) screening programs have been widely adopted in developed countries because of their ability to detect malignant lesions early, thereby reducing mortality and improving survival (1,2). In Spain, an incidence of 34,000 cases and 6,600 deaths due to BC were estimated in 2020 (3). The screening program in the Spanish region of Catalonia is organised by the public health system. This screening program entails sending a postal letter to women between 50 and 69 years of age every 2 years to undergo mammography in one health centre for free pay (4). Several research and communication campaigns have focused on increasing women's adherence to screening to raise awareness of its importance. This is to the effect that the Spanish coverage reached 81.5% in 2017 in the target population (and has dropped to 73.7%, 3 years later, in a pandemic situation) (5). The most cited reason for attending is the invitation letter issued by the organisations of the screening program (6).

However, research also recognises the adverse effects of BC screening, which are usually not explained in a balanced way to women (7). Adverse effects include false positives, false negatives, overdiagnosis, and therefore over-treatment (8). Overdiagnosis refers to screen detected malignancy that would not have progressed to clinical or symptomatic disease and would never have caused a health problem during the lifetime of the women (9). Currently, it is not possible to identify which lesions may progress. Therefore, all lesions are usually treated.

In cases of uncertainty, the literature recommends Shared Decision-making (SDM) (10). It is based on more participatory care, so that women, based on their own beliefs and values, reach an agreement with the health professional (11) to undergo mammography, considering scientific evidence and available options. SDM has shown multiple benefits (12); however, examining its costs, quality, and efficiency (12) to determine its applicability in different contexts is necessary. Some studies indicate that the application of SDM can mean a reduction in health costs (13), while other studies mention that such savings are unclear (14,15). Thus, from health economics perspective, an approach to cost-benefit analysis can be generated through Discrete Choice Experiments (DCE) (16).

The DCE allows the determination of the utility of more participatory health care in the screening of BC through the preferences declared by women, as well as monetising those intangible values that improve the satisfaction of patients (17,18).

There is a dearth of studies focusing on knowing patients' preferences in an SDM (19), and none on women's preferences for the BC screening program and their willingness to pay (WTP) for it. Therefore, this study aimed to analyse the declared preferences of women on the attributes of more participatory care (SDM) in contrast to the usual care with characteristics of a paternalist model for BC screening, establishing the relative importance of each through a DCE. In addition, the study participants were asked about their WTP for this type of health care.

For peer review only

2. METHODS

Our DCE design includes eight hypothetical profiles -choice sets- each with three characteristics -attributes- that can present different values -levels-. For the conjugation of these attributes and levels, a factorial design is considered, and it is assumed that they are independent of each other. Finally, each *choice set* contrasts with another, forming a *choice task* (20) that is presented to women to choose from as per their preference.

2.1. Definition of attributes and levels

The Three Talk model describes and simplifies the implementation of the SDM in three steeps (21,22). The attributes and levels of this experiment were defined taking as reference that model adapting it to the characteristics of the current breast cancer screening program:

1) Information talk: The patients are aware that they must make a decision and know the risks and benefits as well as the pros and cons of the available therapeutic options. For the breast cancer screening the women can be informed through a leaflet **or** by a health professional.

2) **Dialogue talk:** The knowledge of the patient is checked and the information is described in greater detail, as well as the patient`personal preferences between the different options are explored. In our case, there are two options**;** women communicate and discuss their preferences, fears, beliefs about breast cancer screening with a healthcare professional, **or** the health system's schedule for mammography.

3) **Decision talk**: The professional should focus on the deliberative process and whether the patient is prepared to decide or requires support to make the decision. In this case we focus on who has the ability to decide to participate in breast cancer screening: the healthcare professional, the woman, **or** both.

The current functioning of the screening program in a Spanish region, Catalonia, was used to determine the levels for each attribute: the health system schedules a mammography appointment by invitation letter every 2 years to women between 50 and 69 years of age; an information leaflet is attached to the invitation letter and the women decide whether to attend or not. Conversely, in relation to SDM, women inform themselves through a health professional, meet with him/her to discuss their beliefs about screening, and decide whether or not a woman should undergo mammography.

As a result, two levels were established for 'Information talk' and 'Dialogue talk' attributes representing the functioning of the SDM; the usual model and 3 levels were contemplated for the last attribute 'Decision talk' (Table 1).

Table 1. Attributes and levels for options of Shared Decision-making in breast cancer screening

Attribute	Description	Levels

**1. Information talk**	How the information is obtained	1. Leaflet<br>2. Healthcare professional
**2. Dialogue talk**	Dialogue for mammography scheduled	1. No, health system schedules mammography<br>2. Yes, women share and discuss with a healthcare professional their beliefs and values about mammography.
**3. Decision talk**	Who makes the decision	1. The healthcare professional makes the decision<br>2. Shared-decision making by the healthcare professional and the woman<br>3. The woman makes the decision

2.2. Experimental Design

In this phase, attributes and levels are combined to create different choice sets that will be evaluated by women. The number of choice sets depends on the number of attributes and their levels. Following Reed Johnson et al.'s suggestions (23), four-choice sets were removed because they were implausible combinations and not consistent with the theory, leaving a total of eight choice sets. Those were eliminated if, without prior discussion, the professional or jointly, professional and women, decided for attending –or not- the screening, independent if they received the information with a leaflet or by a health professional.

The eight choice sets combined in pairs resulted in a maximum of 28 tasks (the action of choosing between two alternatives). Many of these could be eliminated, such as in the 16 cases in which one alternative dominated or was dominated by the other. Since SDM is considered a better option to resolve the uncertainty of this context (24), it was considered as a dominant choice set. The remaining 12 choice tasks were incorporated into the DCE survey, as this number is below the maximum number of tasks that can be included without causing participants to saturate (25).

2.3. Pre-test and refinement of the questionnaire

In March 2021, snowball sampling was carried out, and 11 women answered the questionnaire to identify the level of difficulty and understand the questions. Based on their comments, it was determined that the previous formulation of the questions had a conventional structured form to a descriptive form that included all the three attributes in one sentence (Figure 1). Subsequently, a randomised pilot study was conducted on 10 women to evaluate the recruitment process and the application of the survey, which was of a mixed form: online system (self-applied) and telephone system (guided) (26).

Figure 1. Choice task example of Shared Decision-making in breast cancer screening

For peer review only

The survey was conducted between June and November of the same year in Spanish and Catalan. On the 12 choice task corresponding to the DCE, seven questions on sociodemographic (age, nationality, marital status, education, employment status, history of family breast cancer, and participation in screening) and a single, multiple-choice question on WTP for breast cancer screening care with SDM characteristics were incorporated (Annex 1).

2.4. Participants

The study was conducted in Catalonia, Spain, using a random sample of women aged 50–60 years who participated in three breast cancer screening programs: Hospital del Mar in Barcelona, Cancer Prevention and Control Program of the Catalan Institute of Oncology, and the Health Region of Lleida. This population had already been invited at least once by the national program to undergo mammography. Therefore, it was assumed that the topic was more sensitive to their interests. Women who had been diagnosed with breast cancer were excluded from the study.

The minimum required sample size was estimated to be 63 participants, calculated based on the empirical rule recommended by Johnson and Orme (27).

$$N>(500c)/(t*a).$$

N=number of respondents, t=number of choice tasks, a=number of alternatives, and c= maximum number of levels

2.5. patient and public involvement

The patients and public was involvement on the pilote survey. So, the women gave recommendation to researcher for edited the final version.

2.6. Data analysis

A mixed-effects conditional logit model was used to estimate women's preferences for different levels of SDM attributes in breast cancer screening. This is based on Daniel McFadden's theory of discrete choice, which seeks to describe the behaviour of decision-makers in the face of a decision problem, assuming that the declared preferences of those elections are based on obtaining the maximum possible utility (28). The mixed-effects logistic regression model allows for the heterogeneity of preferences in the sample by treating the coefficients as random. It also allows multiple observations from each respondent, appropriate for our study, where each woman was presented with 12 choice tasks. All models included main effects without interaction terms. A model for each subgroup was fitted to allow for comparisons according to the WTP.

All the attribute variables were coded as dummy variables, with reference levels identified in tables with the results; further, they were specified as having a random component, assuming a normal distribution for all model coefficients. The coefficients indicate a change in preference from the reference level for each attribute (29).

For peer review only

When interpreting the model results, the statistical significance of the coefficients indicates whether attribute levels influence the choice set, whereas the coefficient size indicates the relative importance of one attribute level to another. We did not include an alternative-specific constant variable because our choice sets were unlabelled. Therefore, they had no utility beyond the attributes assigned to them in the experiment.

For all analyses, statistical significance was set at 0.05. All statistical analyses were performed using R statistical software.

3. RESULTS

Of the 292 women invited to participate, 134 refused, 92 accepted but did not reply, and 66 submitted the survey (reflecting a 22.6% response rate), with one of them excluded due to a history of BC. Therefore, a total sample of 65 women was obtained, of which 2 responses were obtained by telephone and 63 via online forms (Figure 2). The participants' characteristics are presented in Table 2.

Figure 2. Flowchart of participants

Table 2. Characteristics of participants

**Characteristic**		
Age (years)	56.9	(1.34)
Birthplace		
Spain	57	(87.7%)
Other country	8	(12.3%)
Civil status		
Single	17	(26.2%)
With a partner	48	(73.8%)
Education level		
Primary	18	(27.7%)
Secondary	26	(40.0%)
University	21	(32.3%)
Main occupation		
Unemployed	6	(9.23%)
Paid work	48	(73.8%)
Household chores	11	(16.9%)
Paid work		
No	17	(26.2%)
Yes	48	(73.8%)
Family history of breast cancer		
No	50	(76.9%)

For peer review only

Yes	14	(21.5%)
Unknown	1	(1.54%)
Do you have your mammogram regularly?		
No	11	(16.9%)
Yes	54	(83.1%)

Categorical variables summarized with frequency and percentage. Age is summarized with the mean and the standard deviation.

3.1. Discrete Choice Experiment Results and Trade–off

According to the mixed-effects conditional logit model, the weighting for each attribute was estimated using the responses of the 12 tasks. There were no missing values in the answers.

The results show that the attribute 'Decision talk' was the only one important to women when considering SDM in breast cancer screening. Women preferred to make decisions alone (coefficient=2.879; 95%CI=2.297, 3.461) or shared decision-making with healthcare professionals (coefficient=2.375; 95%CI=1.573, 3.177), instead of the healthcare professional making it for them (Table 3). The marginal rate of substitution (MRS) between the two levels of who made the decision was 1.21. This meant that women perceive 20% more utility when making decisions by themselves than when they do with a healthcare professional.

The attributes of 'Information talk' and 'Dialogue talk' have no significant results. Therefore, women would be indifferent between receiving information on the benefits and risks of mammography through a leaflet or being informed by a healthcare professional (coefficient=-0.168; 95%CI=-0.665 , 0.329) and having a meeting with a healthcare professional to discuss about their preferences and beliefs for scheduling the next mammography instead of standard screening schedule (coefficient=0.145; 95%CI=-0.373 , 0.663).

Table 3: Results from the mixed-effects conditional logit model

Attribute	Levels	Coefficient/SD	SE	p-value
**1. Information talk**	Leaflet	Reference category	-	-
	Healthcare professional, mean	-0.168	0.2536	0.509
	Healthcare professional, SD	1.473	0.3206	<0.001
**2. Dialogue talk**	No, health system schedules mammography	Reference category	-	-
	Yes, women share and discuss with a	0.145	0.2645	0.583

For peer review only

	healthcare professional their beliefs and values about mammography, mean			
	Yes, women share and discuss with a healthcare professional their beliefs and values about mammography, SD	1.371	0.3212	<0.001
**3. Decision talk**	The healthcare professional makes the decision	Reference category	-	-
	Shared decision making by the healthcare professional and the woman, mean	2.375	0.4093	<0.001
	Shared decision making by the healthcare professional and the woman, SD	1.415	0.5208	0.007
	The woman makes the decision, mean	2.879	0.2967	<0.001
	The woman makes the decision, SD	1.988	0.2626	<0.001

SD, standard deviation (of the random effects); SE, standard error.

3.2. Willingness to pay for SDM

More than half of the participants (52.3%) were willing to pay for the SDM process in BC screening; most (38.5%) would be willing to pay between 10€ and 30€, and the rest (13.8%) would be willing to pay 40€. (Table 4).

Table 4: Willingness-to-pay woman for an SDM in BC screening

For peer review only

Co-payment (euros)	n	%
0	31	(47.7%)
10	6	(9.23%)
20	6	(9.23%)
30	13	(20.0%)
40	2	(3.08%)
60 or more	7	(10.8%)

Two regression models were fitted to the sample of women according to their WTP: those who showed WTP and those who did not (Table 5). The results for both subgroups were similar to those obtained for the entire sample. Thus, the attribute 'who makes the decision' was the only one important to women regardless of their WTP.

Furthermore, the results for the subgroups show the same trends in women's preferences about 'Information talk´ and 'Dialogue talk' as in the regression model for the entire sample.

Table 5: Results from the mixed-effects conditional logit model stratified by willingness-to-pay

		No willing-to-pay			Willing-to-pay		
**Attribute**	**Levels**	**Coef./SD**	**SE**	**p-value**	**Coef./SD**	**SE**	**p-value**
**1. Information talk**	Leaflet	Ref.	-	-	Ref.	-	-
	Healthcare professional, mean	-0.323	0.3706	0.383	-0.074	0.3768	0.845
	Healthcare professional, SD	1.537	0.4233	<0.001	1.434	0.4684	0.002
**2. Dialogue talk**	No, health system schedules mammography	Ref.	-	-	Ref.	-	-
	Yes, women share and discuss with a healthcare professional their beliefs and values about	0.187	0.3944	0.636	0.136	0.3825	0.721

	mammography, mean						
	Yes, women share and discuss with a healthcare professional their beliefs and values about mammography, SD	1.599	0.4653	<0.001	1.479	0.4756	0.002
**3. Decision talk**	The healthcare professional makes the decision	Ref.	-	-	Ref.	-	-
	Shared decision making by the healthcare professional and the woman, mean	2.220	0.5852	<0.001	2.826	0.6392	<0.001
	Shared decision making by the healthcare professional and the woman, SD	1.449	0.5600	0.010	1.649	0.8826	0.062
	The woman makes the decision, mean	2.784	0.4220	<0.001	3.006	0.4328	<0.001
	The woman makes the decision, SD	1.803	0.3734	<0.001	2.548	0.4410	<0.001

Coef., coefficient; SD, standard deviation (of the random effects); SE, standard error; Ref., reference category.

For peer review only

4. DISCUSSION AND CONCLUSION

4.1. Main findings

This study analysed women's preferences for the attributes -Information talk, Dialogue talk, and Decision talk- of Shared Decision-making in a breast cancer screening program through

a DCE. The main result is that women prefer to make decisions themselves or together with the healthcare professional, but they exclude that the healthcare professional makes it for them. Women perceive 20 percent more efficacy in deciding than in making it jointly with the health professional. More than half of the women were willing to pay for screening with SDM characteristics. There was no difference in the responses obtained between women with a positive WTP and those who did not.

4.2. Attributes implications

For the first attribute, ***Information talk***, the women in our study did not show a clear preference for an information leaflet where they were explained in a simple and balanced way the benefits and risks of screening or that this was done by a health professional. The lack of difference between the two alternatives could be related to the fact that women do not perceive a difference in the information received. This fact would be supported by studies such as the one by Longo et al. (19), who performed a DCE to know the SDM preferences of patients in the context of chronic diseases when the doctor was receiving, or not, some training for the use of risk communication tools. They reported that the second most important attribute for patients was that information was easy to understand. Therefore, using a patient decision aid (PtDA) could be adequate according to the preferences of women.

PtDAs deliver balanced information on the risks and benefits of BC screening, which increases informed decision-making (IDM) (30). In a recent study in Spain (31), 23% of women who received information through this route made informed decisions about their participation in screening, compared to 0.5% in the control group, who only received information attached to the invitation letter by the screening program. PtDAs would also have the advantage of being repeatedly consulted by women to review information, as opposed to a clinical visit. However, both strategies could be complementary, and PtDAs could prepare appointments for the latter to resolve doubts and review the information contained in PtDAs (32).

Some institutions, such as the NHS Cancer Screening Programs, have already incorporated the invitation letter for screening a leaflet with information on the explicit need to make a decision: overdiagnosis, the balance of benefits and risk, and scientific uncertainties (33). Documentation is scarce in our country, and until recently, various programs have not provided balanced information on the benefits and adverse effects of early detection testing (34).

Conversely, a PtDA could be a good alternative for participants with a higher educational level, which is associated with greater literacy in health. Therefore, a greater ability to understand complex data such as prevalence and absolute risks (32). It should be considered that the literature also points out that the higher the educational level the higher the interest of an SDM (19) since these have better access to information to be contrasted with the professional (35,36). This difference may also be due to the type of decisions and contexts. In our particular case, we targeted preventive programs, and the other studies mentioned were associated with disease processes.

For peer review only

Regarding the second attribute, ***Dialogue talk*** , the women in our study were indifferent between attending a presential appointment with a health professional to discuss their beliefs and concerns about participation in the screening or the standard way in which the healthcare system schedules the screening data. These results may be due to the wide acceptance of screening among women, due to the recognised benefits in addition to the minimisation of risks, which have been disseminated by persuasive public health preventive campaigns of different governments. This has led women to unquestionably participate in screening and not necessarily hesitantly discuss the benefits or risks, as well as their beliefs and fears about screening (37).

However, in a study by Longo et al. (2006) (19), the most relevant attribute for patients was their need to feel heard by their physicians, particularly because patients currently report not feeling heard (38). Thus, communication remains an element to be considered by healthcare professionals when they interact with their patients. Having a receptive attitude, acceptance, not judging, and empathy are the first steps to generate a trusting relationship and thus advance to a more participatory model of health (39). Without this, it is impossible to know the beliefs of patients and, therefore, encourage them to participate in the process of shared decision-making. This requires training in communication and transversal skills (38).

Conversely, there is a high probability that women already have beliefs or fears about BC and they want to discuss them with a health professional. In particular, if we consider that having breast cancer and surviving it is not unusual (6), it makes women usually know other women already diagnosed or treated for BC. In our study, one in five women had a first-degree relative with a history of BC. This would increase if it were extended to other affective bonds such as friendships and other familiar or known people. This is an interesting point when you consider personalisation of screening, where women must be assisted by a healthcare professional to determine whether their risk is high, medium, or low, and thus, make recommendations for the frequency of screening in an SDM process (40).

In the third attribute, ***Decision talk***, women reject a paternalistic health model, in which the healthcare professional or the system makes health decisions on their own. This result is consistent with the current trend of empowering patients and giving them more autonomy in any clinical decision (30,33). However, this degree of autonomy may be unrealistic because of the lack of knowledge about adverse effects (41). For example, a recent study indicated that Spanish women have low awareness of overdiagnosis (42,43). Only 8.1% knew the meaning of overdiagnosis, although it increased to 54.2% in women who received decision aid (43).

In the study by Longo et al. (19), patients preferred to make decisions for themselves or with the health care professional and rejected the decision being made by a professional on their own, which coincides with our results. However, Longo's study this attribute was one of the least important.

The characteristics of the clinical process are different when it comes to deciding on treatment than when working in an early detection context, where the target population is healthy. An option that has been well evaluated by women in previous studies (44) is to recommend the development of a targeted SDM to specific groups of women, such as women

For peer review only - http://bmjopen.bmj.com/site/about/guidelines.xhtml

who need more information on the benefits and risks, those who have their first experience in screening, women who have lower educational levels, and those who have a high risk of being diagnosed with breast cancer. All of these strongly require face-to-face appointments with healthcare professionals (32,45). However, the implementation of Informed Decision-making (IDM) for women with a higher level of education and a low risk of being diagnosed with BC would be sufficient, and an appointment with a healthcare professional would not be essential (46). This second group can be given PtDAs with simple and clear information on screening to make the decision autonomously (30,47,48), which implies a lack of decisión follow-up by the healthcare professional. A study is currently being conducted at the Mayo Clinic to determine whether women's discussion groups for decision-making could be a new line to support and prepare women for deciding on breast cancer screening (49). This is based on the strategy mentioned above: the PtDAs could be delivered before the clinical meeting, and a conversation with other women about the information provided there would subsequently take place. As such, the appointment between the healthcare professional and the woman would aim only at the resolution of doubts or concerns born from the PtDAs (32).

In conclusion, the results of the DCE allow us to analyse SDM barriers and the difficulties in its applicability (36,50,51). As such, the main approaches have been developed in contexts where decision-making is particularly difficult (51). Therefore, there are still few examples of application in primary care, where decisions that could be considered less difficult but not less important for their impact on people's quality of life (47).

4.3. Willingness to pay

The subgroups separating those who were or were not willing to pay showed a preference for making decisions jointly or alone. No studies were found on the WTP for SDM in the context of BC screening. Only one article was found for patients with BC diagnosis, but the results were focused on the WTP according to the type of treatment and not on the SDM characteristics (52). Another study reported a prostate cancer study in which WTP participants preferred an active versus passive participation model (53).

Regarding the monetary value of an SDM. This can be compared with the results of Wilson et al. (53) for patients with prostate cancer in the United States. It was determined that the men had WTP between $25 and $50 for the implementation of an SDM (53). WTP was associated with marital status (single people valued more SDM), stage of SDM (those who were initiating the process of decision making preferred to pay versus those who were close to deciding), and the participation model (more active patients prefer to pay versus passive patients) (53).

In addition, Brito et al. (2020) (54) evaluated patient-reported experiences in the adult population in outpatient care through a DCE, in which SDM characteristics were included. In this case, respondents would pay, on average, €16 for a doctor who provides easy-to-understand explanations in relation to one who does not provide understandable explanations. They would also pay, on average, €20 for physicians who can ask questions or raise concerns, versus physicians who focus on providing information. Finally, they would pay, on average,

For peer review only

€22 to physicians who involve the patient in making decisions in contrast to those who make decisions in a one-way manner (54).

Finally, it should be considered that in Spain, screening is fully covered by the National Health System (NHS) for women aged 50–69 years. although this is free cost, and therefore the availability to pay does not effectively coincide with the real payment, the WTP allows us to know the value that the society gives by leaving other services to consume for a innovation of the attention model on appointment (55), and in the case of innovation being implemented in the NHS could imply a burden for the same health system, copayment or direct payment to women (13). In the latter case, the cost of pocket to perform screening is one of the determining elements for adherence to screening (56).

4.4. Limitations

The main limitation of the study, which forced us to change the design of the questions, was the difficulty for women to compare hypothetical profiles; we detected difficulties in understanding the differences between the profiles proposed in each choice task. In addition, their ability to detect differences between the 12 questions in the DCE survey was limited. There may be a tendency to choose models that have already been experienced in contrast to other unknown ones, such as the case of SDM.

Finally, it is difficult to precisely determine the availability to pay, since in Spain the screening of BC belongs to the NHS. Therefore, the services are free at the time of receiving them, and the population assumes that this is a consolidated health benefit and does not consider it should pay, more so when screening for other types of cancer (colorectal, for example) is currently being introduced, in which the NHS is strongly promoting.

4.5. Conclusion

This is the first study to determine women's stated preferences for BC screening when SDM is applied. Women reject a paternalistic health model and favour one with SDM or a variation of it (IDM). In contrast, more than half of the participants were willing to pay for active involvement in their health decisions.

Future research could include randomised clinical trials, in which women would experience health care with all the attributes that SDM elicits and then analyse their preferences, perceived satisfaction, and profit in contrast to regular attendance, and to identify the costs that the implementation of SDM would entail in the screening of BC for the NHS.

4.6. Practice Implications

The change from a paternalistic model to a participatory model of person-centred medicine would require restructuring of the BC screening program so that women could make informed decisions for themselves or SDM together with the health professional.

5. CONTRIBUTION STATEMENT

María José Hernández-Leal, María José Pérez-Lacasta and **Misericòrdia Carles-Lavila**: Conceptualization, Methodology, Formal analysis, Investigation, Project administration,

For peer review only

Supervision, Visualization, Writing-original draft. **Angels Cardona** and **Núria Codern-Bové**; Data curation, Formal analysis, Writing-review & editing. **Carmen Vidal-Lancis**: Resources, Writing-review & editing. **Montserrat Rué**: Resources, Formal analysis, Writing-review & editing. **Carles Forné**: Software, Formal analysis, Writing-review & editing.

6. COMPETING INTERESTS

The authors declare that they have no conflict of interest

7. FUNDING INFORMATION

Financial support for this study was provided entirely by a grant from Instituto de Salud Carlos III through the project PI18/00773 (Co-funded by European Regional Development Fund). European Union's Horizon 2020 research and innovation programme under the Marie Skłodowska-Curie grant agreement No. 713679 from the Universitat Rovira i Virgili (URV). The funding agreement ensures the authors independence in designing the study, interpreting data, writing and publishing the report.

8. DATA SHARING STATEMENT

All data relevant to the study are included in the manuscript and tables or uploaded as supplementary information.

9. ETHICAL APPROVAL STATEMENT

This research was approved by the Medicinal Product Research Ethics Committee (CEIm) of the Institut d'Investigació Sanitària Pere Virgili (Pere Virgili Health Research Institute) Ref. CEIM: 175/2019. Informed consent was obtained, which stated that the participants accepted the conditions of participation upon agreeing to respond to the questionnaire. These conditions specified that the responses were confidential and would be used only for this research.

10. REFERENCES

1. Kerlikowske K, Grady D, Rubin SM, Sandrock C, Ernster VL. Efficacy of screening mammography. A meta-analysis. JAMA. 1995;273(2):149-154. PMID: 7799496
2. Walter LC, Lewis CL, Barton MB. Screening for colorectal, breast, and cervical cancer in the elderly: a review of the evidence. Am J Med. 2005;118(10):1078-1086. doi: 10.1016/j.amjmed.2005.01.063.
3. Sung H, Ferlay J, Siegel RL, Laversanne M, Soerjomataram I, Jemal A, et al. Global cancer statistics 2020: GLOBOCAN estimates of incidence and mortality worldwide for 36 cancers in 185 countries. CA Cancer J Clin. 2021;71(3):209-249. doi:https://doi.org/10.3322/caac.21660.
4. Institut Català d'Oncologia, de Càncer de Mama DP. [Early breast cancer detection]. 2020. Available from: http://gencat.cat/ca/el-cancer/programes_de_deteccio_precoc/programa_de_deteccio_precoc_de_cancer_de_mama/index.html.

For peer review only

5. Ministerio de Sanidad, Servicios Sociales e Igualdad, Instituto Nacional de Estadística. Encuesta europea de Salud en España EESE 2020 [European survey of health in Spain EESE 2020]. Madrid: ministerio de sanidad. 2020. Available from: https://www.sanidad.gob.es/estadEstudios/estadisticas/EncuestaEuropea/EncuestaEuropea2020/EESE2020_inf_evol_princip_result.pdf.
6. Ministerio de Sanidad, Servicios Sociales e Igualdad, Instituto Nacional de Estadística. Encuesta europea de salud en España EESE 2017 [European survey of health in Spain EESE 2017]. 2010. Disponible enhttps:. Available from: http://www.mscbs.gob.es/estadEstudios/estadisticas/encuestaNacional/encuestaNac2017/DETECCION_PRECOZ_CANCER.pdf.
7. Jørgensen KJ, Klahn A, Gøtzsche PC. Are benefits and harms in mammography screening given equal attention in scientific articles? A cross-sectional study. BMC Med. 2007;5:12. doi: 10.1186/1741-7015-5-12.
8. Marmot MG, Altman DG, Cameron DA, Dewar JA, Thompson SG, Wilcox M. The benefits and harms of breast cancer screening: an independent review. Br J Cancer. 2013;108(11):2205-2240. doi: 10.1038/bjc.2013.177.
9. Houssami N. Overdiagnosis of breast cancer in population screening: does it make breast screening worthless? Cancer Biol Med. 2017;14(1):1-8. doi: 10.20892/j.issn.2095-3941.2016.0050.
10. Hargraves IG, Montori VM, Brito JP, Kunneman M, Shaw K, LaVecchia C, Wilson M, Walker L, Thorsteinsdottir B. Purposeful SDM: A problem-based approach to caring for patients with shared decision making. Patient Educ Couns 2019102. 2019;102(10):1786-1792. doi: 10.1016/j.pec.2019.07.020. PMID: 31353170; PMCID: PMC6717012.
11. Elwyn G, Edwards A, Kinnersley P, Grol R. Shared decision making and the concept of equipoise: the competences of involving patients in healthcare choice. Br J Gen Pract. 2000;50:892-897. Available from: https://bjgp.org/content/bjgp/50/460/892.full.pdf.
12. Elwyn G, Frosch DL, Kobrin S. Implementing shared decision-making: consider all the consequences. Implement Sci. 2016;11:114. doi: 10.1186/s13012-016-0480-9. PMID: 27502770; PMCID: PMC4977650.
13. Walsh T, Barr PJ, Thompson R, Ozanne E, O'Neill C, Elwyn G. Undetermined impact of patient decision support interventions on healthcare costs and savings: systematic review. BMJ. 2014;348:g188. doi: 10.1136/bmj.g188. PMID: 24458654; PMCID: PMC3900320.
14. Katz SJ, Hawley S. The value of sharing treatment decision making with patients: expecting too much? JAMA. 2013;310(15):1559-1560. doi: 10.1001/jama.2013.278944. PMID: 24061082.
15. Oshima Lee E, Emanuel EJ. Shared decision making to improve care and reduce costs. N Engl J Med. 2013;368(1):6-8. doi: 10.1056/NEJMp1209500. PMID: 23281971.
16. Ryan M, Gerard K. Using discrete choice experiments to value health care programmes: current practice and future research reflections. Appl Health Econ Health Policy. 2003;2(1):55-64. PMID: 14619274.

For peer review only

17. Ryan M, Bate A, Eastmond CJ, Ludbrook A. Use of discrete choice experiments to elicit preferences. Qual Health Care. 2001;10;Suppl 1:i55-60. doi: 10.1136/qhc.0100055: 11533440; PMCID: PMC1765744.
18. Taguchi R, Yamazaki Y, Nakayama K. Eliciting preferences for mammography: using a discrete choice experiment approach. Nihon koshu eisei zasshi. 2010;57(2):83-94. PMID: 20415238.
19. Longo MF, Cohen DR, Hood K, Edwards A, Robling M, Elwyn G, Russell IT. Involving patients in primary care consultations: assessing preferences using discrete choice experiments. Br J Gen Pract. 2006 ;56(522):35-42. PMID: 16438813; PMCID: PMC1821413.
20. Norman R, Craig BM, Hansen P, Jonker MF, Rose J, Street DJ, Mulhern B. Issues in the design of discrete choice experiments. Patient. 2019;12(3):281-285. doi: 10.1007/s40271-018-0346-0. PMID: 30446958.
21. Elwyn G, Frosch D, Thomson R, Joseph-Williams N, Lloyd A, Kinnersley P, Cording E, Tomson D, Dodd C, Rollnick S, Edwards A, Barry M. Shared decision making: a model for clinical practice. J Gen Intern Med. 2012 Oct;27(10):1361-7. doi: 10.1007/s11606-012-2077-6. Epub 2012 May 23. PMID: 22618581; PMCID: PMC3445676.
22. Elwyn G, Durand MA, Song J, Aarts J, Barr PJ, Berger Z, Cochran N, Frosch D, Galasiński D, Gulbrandsen P, Han PKJ, Härter M, Kinnersley P, Lloyd A, Mishra M, Perestelo-Perez L, Scholl I, Tomori K, Trevena L, Witteman HO, Van der Weijden T. A three-talk model for shared decision making: multistage consultation process. BMJ. 2017;359:j4891. doi: 10.1136/bmj.j4891. PMID: 29109079; PMCID: PMC5683042.
23. Reed Johnson F, Lancsar E, Marshall D, Kilambi V, Mühlbacher A, Regier DA, Bresnahan BW, Kanninen B, Bridges JF. Constructing experimental designs for discrete-choice experiments: report of the ISPOR Conjoint Analysis Experimental Design Good Research Practices Task Force. Value Health. 2013;16(1):3-13. doi: 10.1016/j.jval.2012.08.2223. PMID: 23337210.
24. Hernández-Leal MJ, Carles-Lavila M, Pérez-Lacasta M. La participación de los profesionales de la salud en la Toma de Decisiones Compartida en el cribado de cáncer de mama: manual de apoyo para implementación de la TDC. [The participation of health professionals in shared decision-making in breast cancer screening: A support manual for the implementation of SDM]. Spain: María José Hernández editor. 2021. Available from: https://www.researchgate.net/publication/358277309_The_Participation_of_Health_Professionals_in_Shared_Decision-Making_on_Breast_Cancer_Screening
25. Ryan M, Bate A. Testing the assumptions of rationality, continuity and symmetry when applying discrete choice experiments in health care. Appl Econ Lett. 2001;8(1):59-63. doi: 10.1080/135048501750041312.
26. Mariel P, Hoyos D, Meyerhoff J, Czajkowski M, Dekker T, Glenk K, Jacobsen JB, Liebe U, Olsen SB, Sagebiel J, Thiene M. 2020. Environmental valuation with discrete choice

experiments: guidance on design, implementation and data analysis. Springer;2020, pp 54-58. Available from: http://eprints.whiterose.ac.uk/168622/.

27. de Bekker-Grob EW, Donkers B, Jonker MF, Stolk EA. Sample size requirements for discrete-choice experiments in healthcare: a practical guide. Patient. 2015;8(5):373-384. doi: 10.1007/s40271-015-0118-z. PMID: 25726010; PMCID: PMC4575371.
28. McFadden D. Conditional logit analysis of qualitative choice behavior. 1973. Zarembka, P. Frontiers in econometrics. Academic Press: New York, pp 105-142.
29. Herrera C. Valoración y modelado de las preferencias en calidad de vida relacionadas con la salud de menores [Valuation and modeling of health related quality of life preferences of younger population]. 2020. Available from: https://riull.ull.es/xmlui/bitstream/handle/915/20075/Valoracion%20y%20modelado%20de%20las%20preferencias%20en%20calidad%20de%20vida%20relacionadas%20con%20la%20salud%20de%20menores..pdf?sequence=1.
30. Hersch JK, Nickel BL, Ghanouni A, Jansen J, McCaffery KJ. Improving communication about cancer screening: moving towards informed decision making. Public Health Res Pract. 2017;27(2):2731728. doi: 10.17061/phrp2731728. PMID: 28765861.
31. Pérez-Lacasta MJ, Martínez-Alonso M, Garcia M, Sala M, Perestelo-Pérez L, Vidal C, et al. Effect of information about the benefits and harms of mammography on women's decision making: the InforMa randomised controlled trial. PLOS ONE. 2019;14(3):e0214057. doi: 10.1371/journal.pone.0214057.
32. Maschke A, Paasche-Orlow MK, Kressin NR, Schonberg MA, Battaglia TA, Gunn CM. Discussions of potential mammography benefits and harms among patients with limited health literacy and providers: 'oh, there are harms?' J Health Commun. 2020;25(12):951-961. doi: 10.1080/10810730.2020.1845256. PMID: 33455518; PMCID: PMC8062298.
33. Forbes LJ, Ramirez AJ, Expert group on Information about Breast Screening. Offering informed choice about breast screening. J Med Screen. 2014;21(4):194-200. doi: 10.1177/0969141314555350. PMID: 25312639.
34. Ballesteros-Peña S, Gavilán-Moral E. Contenido de los documentos informativos dirigidos a las mujeres sobre el cribado de cáncer de mama en España [Content of official addressed to women informative documents about breast cancer screening in Spain]. Rev Esp Salud Publica. 2018;92:e201810076. Spanish. PMID: 30361472.
35. Millaruelo J, Martín F. Toma de decisiones compartidas con el paciente [Shared decision-making with the patient]. Rev Clin Elect en Aten Pri. 2009. Available from. Available from: https://ddd.uab.cat/pub/rceap/rceap_a2009m5n17/rceap_a2009m5n17a9.pdf
36. Hernández-Leal MJ, Pérez-Lacasta MJ, Feijoo-Cid M, Ramos-García V, Carles-Lavila M. Healthcare professionals' behaviour regarding the implementation of shared decision-making in screening programmes: A systematic review. Patient Educ Couns. 2021 Aug;104(8):1933-1944. doi: 10.1016/j.pec.2021.01.032. PMID: 33581968.
37. Hersch J, Jansen J, McCaffery K. Decision-making about mammographic screening: pursuing informed choice. Climacteric. 2018;21(3):209-213, doi: 10.1080/13697137.2017.1406912

38. Holmström I, Rosenqvist U. A change of the physicians understanding of the encounter parallels competence development. Patient Educ Couns. 2001;42(3):271-278. doi: 10.1016/s0738-3991(00)00132-4. PMID: 11164326.
39. Vargas I, Guerrero S, Hernández-Leal M, Campos R. La Confianza en la relación profesional de la salud y paciente [Confidence in the health professional and patient relation] Rev Cubana Salud Pública. 2020;26(3). Available from: http://www.revsaludpublica.sld.cu/index.php/spu/article/view/1575/1634.
40. Sala M, Domingo L, Rué M, Comas M, Torá-Rocamora I, Macià F, Castells X. Investigación en cribado de cáncer de mama: camino hacia estrategias personalizadas y decisiones compartidas [Research on breast cancer screening: toward personalized strategies and shared decision-making]. Revista de Senología y Patología Mamaria. 2014;27(4):176-182. doi: 10.1016/j.senol.2014.07.007.
41. Mathioudakis AG, Salakari M, Pylkkanen L, Saz-Parkinson Z, Bramesfeld A, Deandrea S, Lerda D, Neamtiu L, Pardo-Hernandez H, Solà I, Alonso-Coello P. Systematic review on women's values and preferences concerning breast cancer screening and diagnostic services. Psycho-Oncology. 2019;28(5):939-947. doi: 10.1002/pon.5041. PMID: 30812068; PMCID: PMC6594004.
42. Baena-Cañada JM, Rosado-Varela P, Expósito-Álvarez I, González-Guerrero M, Nieto-Vera J, Benítez-Rodríguez E. Women's perceptions of breast cancer screening. Spanish screening programme survey. Breast. 2014;23(6):883-888. doi: 10.1016/j.breast.2014.09.010. PMID: 25456103.
43. Toledo-Chávarri A, Rué M, Codern-Bové N, Carles-Lavila M, Perestelo-Pérez L, Pérez-Lacasta MJ, et al. A qualitative study on a decision aid for breast cancer screening: views from women and health professionals. Eur J Cancer Care (Engl). 2017;26(3):1-11. doi: 10.1111/ecc.12660.
44. Rainey L, van der Waal D, Donnelly LS, Southworth J, French DP, Evans DG, Broeders MJM. Women's health behaviour change after receiving breast cancer risk estimates with tailored screening and prevention recommendations. BMC Cancer. 2022;22(1):69. doi: 10.1186/s12885-022-09174-3. PMID: 35033023.
45. Esquivel-Sada D, Lévesque E, Hagan J, Knoppers BM, Simard J. Envisioning implementation of a personalized approach in breast cancer screening programs: stakeholder perspectives. Healthc Policy. 2019;15(2):39-54. doi: 10.12927/hcpol.2019.26072. PMID: 32077844; PMCID: PMC7020798.
46. Rimer BK, Briss PA, Zeller PK, Chan EC, Woolf SH. Informed decision making: what is its role in cancer screening? Cancer. 2004;101(5);Suppl:1214-1228. doi: 10.1002/cncr.20512. PMID: 15316908.
47. Caverly TJ, Hayward RA. Dealing with the lack of time for detailed shared decision-making in primary care: everyday shared decision-making. J Gen Intern Med. 2020;35(10):3045-3049. doi: 10.1007/s11606-020-06043-2; PMID: 32779137; PMCID: PMC7572954.
48. Stacey D, Légaré F, Lewis K, Barry MJ, Bennett CL, Eden KB, Holmes-Rovner M, Llewellyn-Thomas H, Lyddiatt A, Thomson R, Trevena L. Decision aids for people facing health treatment or screening decisions. Cochrane Database Syst Rev.

For peer review only

2017;4(4):CD001431. doi: 10.1002/14651858.CD001431.pub5. PMID: 28402085; PMCID: PMC6478132.
49. Hernández-Leal M, Montori V. 2021. Discussion groups for decision-making on breast cancer screening [research proyect]. Mayo Clinic.
50. Légaré F, Ratté S, Gravel K, Graham ID. Barriers and facilitators to implementing shared decision-making in clinical practice: update of a systematic review of health professionals' perceptions. Patient Educ Couns. 2008;73(3):526-535. doi: 10.1016/j.pec.2008.07.018.
51. Légaré F, Witteman HO. Shared decision making: examining key elements and barriers to adoption into routine clinical practice. Health Aff (Millwood). 2013;32(2):276-284. doi: 10.1377/hlthaff.2012.1078.
52. Johnson P, Bancroft T, Barron R, Legg J, Li X, Watson H, Naeim A, Watkins A, Marshall DA. Discrete choice experiment to estimate breast cancer patients' preferences and willingness to pay for prophylactic granulocyte colony-stimulating factors. Value Health. 2014;17(4):380-389. doi: 10.1016/j.jval.2014.01.002. PMID: 24968998.
53. Wilson LS, Blonquist TM, Hong F, Halpenny B, Wolpin S, Chang P, Filson CP, Master VA, Sanda MG, Chien GW, Jones RA, Krupski TL, Berry DL. Assigning value to preparation for prostate cancer decision making: a willingness to pay analysis. BMC Med Inform Decis Mak. 2019;19(1):6. doi: 10.1186/s12911-018-0725-4. PMID: 30626400; PMCID: PMC6327504.
54. Brito Fernandes Ó, Péntek M, Kringos D, Klazinga N, Gulácsi L, Baji P. Eliciting preferences for outpatient care experiences in Hungary: A discrete choice experiment with a national representative sample. PLoS One. 2020;15(7):e0235165. doi: 10.1371/journal.pone.0235165. PMID: 32735588; PMCID: PMC7394384.
55. Puig-Junoy J, Pinto-Prades JL, Ortún-Rubio V. El análisis coste-beneficio en sanidad [Cost-benefit analysis in health care]. Aten Primaria. 2001;27(6):422-7. Spanish. doi: 10.1016/s0212-6567(01)78825-7. PMID: 11334581; PMCID: PMC7677961.
56. Mansfield C, Tangka FK, Ekwueme DU, Smith JL, Guy GP Jr, Li C, Hauber AB. Stated preference for cancer screening: A systematic review of the literature, 1990-2013. Prev Chronic Dis. 2016;13:E27. doi: 10.5888/pcd13.150433. PMID: 26916898; PMCID: PMC4768876.

11. FIGURE LEGEND

Figure 1. Choice task example of Shared Decision-making in breast cancer screening

Figure 2. Flowchart of participants

For peer review only

Figure 1. Choice task example of Shared Decision-making in breast cancer screening

**Option A**	**Option B**
I receive **information about the benefits and risks in a leaflet**, and I also **talk with a healthcare professional** my fears or beliefs about mammography. Finally, **I make the decision** to attend -or not- mammography.	I am **informed by a health professional** about the benefits and risks; my **mammography programs the health system**, so I do not manifest my fears, beliefs or preferences about mammography to a health professional. Finally, **I make the decision** to attend -or not- mammography.

210x115mm (144 x 144 DPI)

Figure 2. Flowchart participants

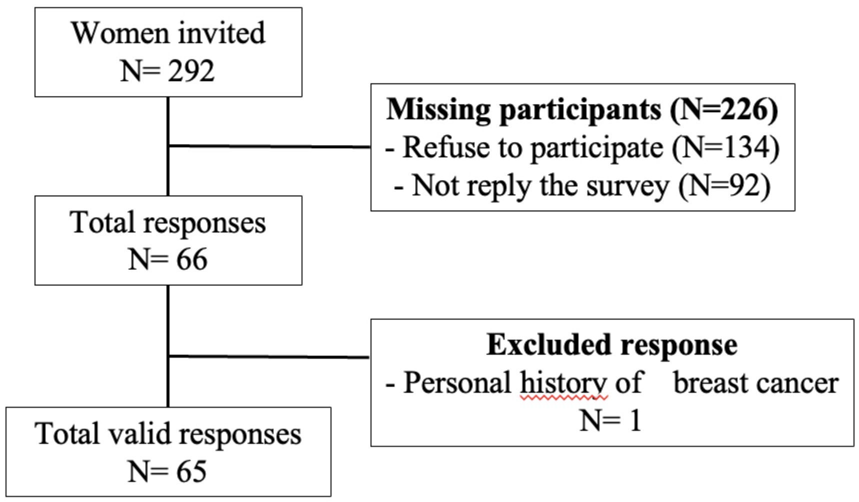

176x119mm (144 x 144 DPI)

"Women's Preferences for Breast Cancer Screening"

We appreciate your collaboration in the ProShare research project "*The collaboration of healthcare professionals to include Shared Decision Making in the breast cancer screening program*" (P18/00773), led by Dr. Misericòrdia Carles. This project is supported and funded by the Carlos III Institute of Health and co-financed by the European Regional Development Fund (ERDF) "A way of making Europe" y "European Union's Horizon 2020 research and innovation program under the Marie Skłodowska-Curie grant agreement" No. 713679 and from the Universitat Rovira i Virgili (URV).

The objective of your participation is to identify women's preferences and determine the cost-benefit of a new care model for the breast cancer screening program. So, we present you, in two hypothetical options and you must choose only one of them according to your preferences. Consider that there are no good or bad answers.

Your answers will be confidential and will only be used for research purposes. Answer the following questions truthfully, considering that your identity will never be exposed.

ProShare
SHARED DECISION MAKING IN HEALTH
Shared Decision Making in Health

1. Socio-demographic characteristics
a. Name: ___________

For peer review only

b. Age (years):______________

c. Birthplace (mark with an X)

	Spain
	Other country

d. Civil status (mark with an X)

	Single
	With partner

e. Education level (mark with an X)

	Primary
	Secondary
	University

f. Main occupation (mark with an X)

	Paid work
	Household chores
	Unemployed

g. Family history of breast cancer (mark with an X)

	Yes
	No
	Unknown

h. Do you have your mammogram regularly? (mark with an X)

	Yes
	No

For peer review only

2. Experimental design

The next 12 questions will be asked in consultation on three characteristics of the breast cancer screening program:

1. **How do I receive the information?** You will find two possibilities; in one of them you are informed about the benefits and risks of mammography through a **leaflet** or by **health professional**.

2. Dialogue about my preferences? You'll find two possibilities; in one, you **meet with a healthcare professional** to discuss your preferences, fears, or concerns about mammography. And on the other hand, **the health system** simply programs its mammography according to its age.

3. Who makes the decision? You will find three options about who makes the decision for attended -or no- to mammography: the **health professional**, **the woman** or **jointly**. Independent if later this is concrete the mammography

In each question you must choose the option you prefer. Each option includes the three features above.

For example:
Question 0: Mark with an "X" the option you prefer

Option A	Option B
I am informed by a **healthcare professional** about the benefits and risks, **I also express my fears**, beliefs, or preferences of mammography. Finally, **I myself made the decision** attend -or not- mammography.	I am informed by a **healthcare professional** about the benefits and risks, **I also express my fears**, beliefs or preferences of mammography. Finally, **we jointly made the decision** attend -or not- to mammography.

We are aware that in some questions the options may be very similar to you, yet there is always some different characteristics There are no correct or incorrect answers, you should select only based on your personal appreciation.

SURVEY

1: Mark with an "X" the option you prefer

Option A	Option B

**The healthcare professionals inform** me about the benefits and risks; **I also tell them about my fears**, beliefs, or preferences for mammography. Finally, **the healthcare professional makes the decision** attend -or not- to the mammography.	**The healthcare professionals inform me** about the benefits and risks; **I also tell them about my fears**, beliefs, or preferences for mammography. Finally, **I myself made the decision** attend -or not- to the mammography.

2.Mark with an "X" the option you prefer

Option A	Option B
I receive information about the benefits and risks through a **leaflet**; **I also share with a healthcare professional my fears** or beliefs about mammography. Finally, **we jointly made the decision** attend -or not- to the mammography.	**I am informed by a healthcare professional** about the benefits and risks; **I also express my fears, beliefs,** or preferences of mammography. Finally, **I myself made the decision** attend -or not- to the mammography.

3. Mark with an "X" the option you prefer

Option A	Option B
I receive information about the benefits and risks through **a leaflet**; **I also tell a healthcare professional my fears** or beliefs about mammography. Finally, **the professional makes the decision** attend -or not- to the mammography.	**The healthcare professionals inform** me about the benefits and risks; **I also tell them about my fears**, beliefs, or preferences for mammography. Finally, **I myself made the decision** attend -or not- to the mammography.

4. Mark with an "X" the option you prefer

Option A	Option B
**I am informed by a healthcare professional** about the benefits and risks, but **without the possibility of expressing my fears** or beliefs about mammography as it is defined by the health program. Finally, **I myself made the decision** attend -or not- to the mammography.	**The healthcare professional informs me** about the benefits and risks**; I also tell them about my fears**, beliefs, or preferences for mammography. Finally, **the healthcare professional makes the decision** attend -or not- to the mammography.

5. Mark with an "X" the option you prefer

Option A	Option B
I receive information about the benefits and risks through **a leaflet**; I also **tell a**	**The healthcare professional informs me** about the benefits and risks; **I also**

For peer review only

**healthcare professional my fears** or beliefs about mammography. Finally, **we jointly made the decision** attend -or not- to the mammography.	**tell them about my fears**, beliefs, or preferences for mammography. Finally, the **healthcare professional makes the decision** attend -or not- to the mammography.

6. Mark with an "X" the option you prefer

Option A	Option B
I receive information about the benefits and risks through **a leaflet**; **I also tell a healthcare professional my fears** or beliefs about mammography. Finally, **I myself made the decision** attend -or not- to the mammography.	**The healthcare professional informs me** about the benefits and risks; **I also tell them about my fears**, beliefs, or preferences for mammography. Finally, **the healthcare professional makes the decision** attend -or not- to the mammography.

7. Mark with an "X" the option you prefer

Option A	Option B
I receive information about the benefits and risks through **a leaflet**; **my mammography programs the health system**, so I do not manifest my fears, beliefs, or preferences about mammography to a health professional. Finally, **I myself made the decision** attend -or not- to the mammography.	**The healthcare professional informs me** about the benefits and risks; **I also tell them about my fears**, beliefs, or preferences for mammography. Finally, the **healthcare professional makes the decision** attend -or not- to the mammography.

8. Mark with an "X" the option you prefer

Option A	Option B
I receive information about the benefits and risks through **a leaflet**; **I also tell a healthcare professional my fears** or beliefs about mammography. Finally, **we jointly made the decision** attend -or not- to the mammography.	**I am informed by a health professional** about the benefits and risks; **my mammography programs the health system**, so I do not manifest my fears, beliefs, or preferences about mammography to a health professional. Finally, **I myself made the decision** attend -or not- to the mammography.

9. Mark with an "X" the option you prefer

Option A	Option B
I receive information about the benefits and risks in a **leaflet; I also share my fears** or beliefs about mammography with a health professional. Finally, **I myself**	**I am informed by a health professional** about the benefits and risks; my mammography programs the health system, **so I do not manifest my fears**, beliefs or preferences about

For peer review only

**made the decision** attend -or not- to the mammography.	mammography with a health professional. Finally, **I myself made the decision** attend -or not- to the mammography.

10. Mark with an "X" the option you prefer

Option A	Option B
I receive information about the benefits and risks in a **leaflet**; **I also share my fears** or beliefs about mammography with a health professional. Finally, **the professional makes the decision** attend -or not- to the mammography.	**I am informed by a health professional** about the benefits and risks; however, **I do not manifest my fears**, beliefs or preferences about mammography as it programs the health system. Finally, **I myself made the decision** attend -or not- to the mammography

11. Mark with an "X" the option you prefer

Option A	Option B
I receive information about the benefits and risks in a **leaflet**; **I also share my fears** or beliefs about mammography with a health professional. Finally, **the professional makes the decision** attend -or not- to the mammography	I receive information about the benefits and risks in a **leaflet**; **I also share my fear**s or beliefs about mammography with a health professional. Finally, **I myself made the decision** attend -or not- to the mammography

12. Mark with an "X" the option you prefer

Option A	Option B
I receive information about the benefits and risks a **leaflet**; **my mammography programs the health system**, so I do not manifest my fears, beliefs or preferences about mammography with a health professional. Finally, **I myself made the decision** attend -or not- to the mammography	I receive information about the benefits and risks in a **leaflet**; **I also share my fears** or beliefs about mammography with a health professional. Finally**, the professional makes the decision** attend -or not- to the mammography

3. Cost of health

The breast cancer screening is currently not paid to women, as it is covered by the public health service. Regardless of the current cost, if you had the option of attending an appointment with a healthcare professional to inform you about the benefits and risks of mammography, discuss with him/her your preferences and concerns and jointly make the decision to attend -or not to mammography **How much money would you be willing to pay?**

	0 euros

For peer review only

	10 euros
	20 euros
	30 euros
	40 euros
	60 euros or more

Thank you so much for the time you took to answer this survey to improve women's care in breast cancer screening.

ProShare

For peer review only

Women's Preference to Apply Shared Decision-Making in Breast Cancer Screening: A Discrete Choice Experiment

STROBE Statement—Checklist of items that should be included in reports of ***cross-sectional studies***

	Item No	Recommendation	Page No
**Title and abstract**	1	(*a*) Indicate the study's design with a commonly used term in the title or the abstract	1
		(*b*) Provide in the abstract an informative and balanced summary of what was done and what was found	2
**Introduction**			
Background/rationale	2	Explain the scientific background and rationale for the investigation being reported	3
Objectives	3	State specific objectives, including any prespecified hypotheses	3
**Methods**			
Study design	4	Present key elements of study design early in the paper	4
Setting	5	Describe the setting, locations, and relevant dates, including periods of recruitment, exposure, follow-up, and data collection	6
Participants	6	(*a*) Give the eligibility criteria, and the sources and methods of selection of participants	6
Variables	7	Clearly define all outcomes, exposures, predictors, potential confounders, and effect modifiers. Give diagnostic criteria, if applicable	5
Data sources/ measurement	8*	For each variable of interest, give sources of data and details of methods of assessment (measurement). Describe comparability of assessment methods if there is more than one group	*5*
Bias	9	Describe any efforts to address potential sources of bias	5
Study size	10	Explain how the study size was arrived at	6
Quantitative variables	11	Explain how quantitative variables were handled in the analyses. If applicable, describe which groupings were chosen and why	6
Statistical methods	12	(*a*) Describe all statistical methods, including those used to control for confounding	6
		(*b*) Describe any methods used to examine subgroups and interactions	6
		(*c*) Explain how missing data were addressed	NA
		(*d*) If applicable, describe analytical methods taking account of sampling strategy	6
		(*e*) Describe any sensitivity analyses	6
**Results**			
Participants	13*	(a) Report numbers of individuals at each stage of study—eg numbers potentially eligible, examined for eligibility, confirmed eligible, included in the study, completing follow-up, and analysed	7
		(b) Give reasons for non-participation at each stage	7
		(c) Consider use of a flow diagram	7
Descriptive data	14*	(a) Give characteristics of study participants (eg demographic, clinical, social) and information on exposures and potential confounders	7-8
		(b) Indicate number of participants with missing data for each variable of interest	7
Outcome data	15*	Report numbers of outcome events or summary measures	8

For peer review only - http://bmjopen.bmj.com/site/about/guidelines.xhtml

Main results	16	(*a*) Give unadjusted estimates and, if applicable, confounder-adjusted estimates and their precision (eg, 95% confidence interval). Make clear which confounders were adjusted for and why they were included	8-9
		(*b*) Report category boundaries when continuous variables were categorized	
		(*c*) If relevant, consider translating estimates of relative risk into absolute risk for a meaningful time period	NA
Other analyses	17	Report other analyses done—eg analyses of subgroups and interactions, and sensitivity analyses	10-12
**Discussion**			
Key results	18	Summarise key results with reference to study objectives	10
Limitations	19	Discuss limitations of the study, taking into account sources of potential bias or imprecision. Discuss both direction and magnitude of any potential bias	15-16
Interpretation	20	Give a cautious overall interpretation of results considering objectives, limitations, multiplicity of analyses, results from similar studies, and other relevant evidence	12-15
Generalisability	21	Discuss the generalisability (external validity) of the study results	15-16
**Other information**			
Funding	22	Give the source of funding and the role of the funders for the present study and, if applicable, for the original study on which the present article is based	16

*Give information separately for exposed and unexposed groups.

Note: An Explanation and Elaboration article discusses each checklist item and gives methodological background and published examples of transparent reporting. The STROBE checklist is best used in conjunction with this article (freely available on the Web sites of PLoS Medicine at http://www.plosmedicine.org/, Annals of Internal Medicine at http://www.annals.org/, and Epidemiology at http://www.epidem.com/). Information on the STROBE Initiative is available at www.strobe-statement.org.

For peer review only

BMJ Open

Women's Preference to Apply Shared Decision Making in Breast Cancer Screening: A Discrete Choice Experiment

Journal:	*BMJ Open*
Manuscript ID	bmjopen-2022-064488.R1
Article Type:	Original research
Date Submitted by the Author:	02-Sep-2022
Complete List of Authors:	Hernández Leal, María José; Universitat Rovira i Virgili, Economic ; Research Group on Statistics, Economic Evaluation and Health (GRAEES).<br>Pérez-Lacasta, María José; Universitat Rovira i Virgili, Economic ; Research Group on Statistics, Economic Evaluation and Health (GRAEES).<br>Cardona, Angels; Area Q: Evaluation and Research in the Field of Social Sciences and Health<br>Codern-Bové, Núria; Autonomous University of Barcelona, School of Nursing and Occupational Therapy (EUIT)<br>Vidal, Carmen; Catalan Institute of Oncology, Cancer Prevention and Control Program<br>Rue, Montserrat; University of Lleida, Basic Medical Sciences; Research Group on Statistics, Economic Evaluation and Health (GRAEES). ,<br>Forné, Carles; University of Lleida, Department of Basic Medical Sciences; Heorfy Consulting, HEOR freelance consultant<br>Carles-Lavila, Misericòrdia; Universitat Rovira i Virgili, Economics; Research Group on Statistics, Economic Evaluation and Health (GRAEES).
**Primary Subject Heading**:	Health economics
Secondary Subject Heading:	Communication, Diagnostics, Health economics, Patient-centred medicine, Research methods
Keywords:	HEALTH ECONOMICS, Breast tumours < ONCOLOGY, PREVENTIVE MEDICINE, PRIMARY CARE

SCHOLARONE™
Manuscripts

I, the Submitting Author has the right to grant and does grant on behalf of all authors of the Work (as defined in the below author licence), an exclusive licence and/or a non-exclusive licence for contributions from authors who are: i) UK Crown employees; ii) where BMJ has agreed a CC-BY licence shall apply, and/or iii) in accordance with the terms applicable for US Federal Government officers or employees acting as part of their official duties; on a worldwide, perpetual, irrevocable, royalty-free basis to BMJ Publishing Group Ltd ("BMJ") its licensees and where the relevant Journal is co-owned by BMJ to the co-owners of the Journal, to publish the Work in this journal and any other BMJ products and to exploit all rights, as set out in our licence.

The Submitting Author accepts and understands that any supply made under these terms is made by BMJ to the Submitting Author unless you are acting as an employee on behalf of your employer or a postgraduate student of an affiliated institution which is paying any applicable article publishing charge ("APC") for Open Access articles. Where the Submitting Author wishes to make the Work available on an Open Access basis (and intends to pay the relevant APC), the terms of reuse of such Open Access shall be governed by a Creative Commons licence – details of these licences and which Creative Commons licence will apply to this Work are set out in our licence referred to above.

Other than as permitted in any relevant BMJ Author's Self Archiving Policies, I confirm this Work has not been accepted for publication elsewhere, is not being considered for publication elsewhere and does not duplicate material already published. I confirm all authors consent to publication of this Work and authorise the granting of this licence.

For peer review only

Original Research
BMJ Open

Women's Preference to Apply Shared Decision Making in Breast Cancer Screening: A Discrete Choice Experiment

Running head: A DCE for SDM in breast cancer screening

María José Hernández-Leal[a,b,c] María José Pérez-Lacasta [a,c] Angels Cardona[d] Núria Codern-Bové[e] Carmen Vidal-Lancis[f] Montserrat Rué[c,g] Carles Forné[h,i], Misericòrdia Carles-Lavila [a,b,c] on behalf of the ProShare Group

a. Department of Economics, University of Rovira i Virgili, Reus, Spain.
b. Research Centre on Economics and Sustainability (ECO-SOS), Reus, Spain.
c. Research Group on Statistics, Economic Evaluation and Health (GRAEES), Reus, Spain.
d. Area Q: Evaluation and Research in the Field of Social Sciences and Health, Barcelona, Spain.
e. School of Nursing and Occupational Therapy (EUIT), Autonomous University of Barcelona, Terrassa, Spain.
f. Cancer Prevention and Control Program, Catalan Institute of Oncology-IDIBELL, L'Hospitalet de Llobregat, Spain
g. Department of Basic Medical Sciences, University of Lleida-IRB, Lleida, Spain.
h. Department of Basic Medical Sciences, University of Lleida, Spain.
i. HEOR freelance consultant, Heorfy Consulting, Reus, Spain.

ProShare Group: Misericòrdia Carles-Lavila, Núria Codern-Bové, María José Hernández-Leal, María José Pérez, Roger Pla, Vanesa Ramos, Carmen Vidal

Corresponding author: Misericòrdia Carles-Lavila, Department of Economic, University of Rovira i Virgili, Avenida Universitat 1, 43204, Reus, Spain. Email: misericordia.carles@urv.cat

For peer review only

ABSTRACT

Objective: To analyse women's stated preferences for establishing the relative importance of each attribute of Shared Decision making (SDM) and their willingness to pay (WTP) for more participatory care in a breast cancer screening programme (BCSP).
Design: A Discrete Choice Experiment was designed with 12 questions (choice tasks). It included three attributes: 'How the information is obtained' regarding benefits and harms, whether there is a 'Dialogue for mammography scheduled' between the healthcare professional and the woman, and, 'Who makes the decision' regarding participation in BCSP. The data were obtained using a survey that included 12 choice tasks, one question on WTP, and seven socioeconomic questions. The analysis was performed using conditional mixed-effects logit regression and stratification according to WTP.
Setting: Data collection related to BCSP was conducted between June and November 2021 in Catalonia, Spain.
Participants: Sixty-five women between 50 and 60 years old.
Main outcome measures: Women's perceived utility of each attribute, trade-off on these attributes, and WTP for SDM in BCSP.
Result: The only significative attribute was 'Who makes the decision'. The decision made by oneself alone (coefficient=2.879; 95% CI=2.297, 3.461) and the decision made together with a healthcare professional (2.375; 95%CI=1.573, 3.177) were the options preferred by women. The former contributes 21% more utility than the latter. Moreover, 52.3% of women stated a WTP 10 or more euros for SDM. Women's preferences regarding attributes did not influence their WTP.
Conclusions: The women refused a current paternalistic model and prefer SDM or Informed Decision making for BCSP.

Keywords: Shared Decision -making; Informed Decision-making; Breast cancer screening; Discrete Choice Experiments; Willingness to pay.

Strengths and limitations of this study

- DCEs have limitations on sample size, and SDM, as a little-known model, makes it difficult for participants to understand the hypothetical situations described in the choice task.
- A DCE is an indirect method to obtain stated preferences and information that are otherwise impossible to reveal when actual choice behaviour is restricted in some way.
- The follow-up plan of this study based on the response level of the participants allowed us to obtain a strong response rate.
- The responses obtained on participants' WTP may be sensitive/not robust when performed in a public health system context.
- This is the first study concerning women's preferences related to SDM regarding BCSPs.

For peer review only

1. BACKGROUND

Breast cancer (BC) screening programmes have been widely adopted in developed countries because of their ability to detect malignant lesions early, thereby reducing mortality and improving survival (1,2). In Spain, 34,000 cases of and 6,600 deaths due to BC were estimated in 2020 (3). The screening programme in the Spanish region of Catalonia is organised by the public health system. This screening programme entails sending a postal letter to women between 50 and 69 years of age every 2 years to undergo mammography in a health centre free of charge (4). Several research and communication campaigns have focused on increasing women's adherence to screening to raise awareness of its importance, which led to the Spanish coverage reaching 81.5% in 2017 in the target population (although it had dropped to 73.7% 3 years later, during the pandemic) (5). The most cited reason for attending is the invitation letter issued by the organisations affiliated with the screening programme (6).

However, research also recognises the adverse aspects of BC screening, which are usually not explained to women in a balanced way (7). These include false positives, false negatives, overdiagnosis, and, consequently, over-treatment (8). Overdiagnosis refers to screen-detected malignancy that would not have progressed to clinical or symptomatic disease and would never have caused health problems during the woman's lifetime (9). Currently, it is not possible to identify which lesions may progress. Therefore, all lesions are generally treated.

In cases of uncertainty, the literature recommends Shared Decision making (SDM) (10). SDM is characterised as a participatory care model, in which the professional and the patient are on par with each other in terms of power to deliberate regarding the best decision. This contrasts with the paternalistic health model, in which the health professional or the system makes such decisions (11). The latter phenomenon remains in force in some health programmes despite declarations and intention to shift to a more participatory one. SDM allows women, based on their own beliefs and values, to decide, together with a health professional, whether to undergo mammography (12) while considering scientific evidence and the options available to them. SDM has shown multiple benefits (13); however, examining its costs, quality, and efficiency (13) to determine its applicability in different contexts is necessary. Some studies indicate that the application of SDM can mean a reduction in health costs (14), while other studies suggest that the degree of such savings is unclear (15,16). Thus, from a health economics perspective, an approach to cost-benefit analysis can be generated through Discrete Choice Experiments (DCEs) (17).

Using a DCE allows for the determination of the utility of more participatory healthcare in the screening of BC through allowing women to declare their preferences as well as through monetising the intangible values that improve patient satisfaction (18,19).

There is a dearth of studies focusing on awareness of patients' preferences in SDM (20), and there are none on women's preferences regarding breast cancer screening programmes (BCSP) and their willingness to pay (WTP) for them. Therefore, this study aimed to analyse the declared preferences of women in Catalonia regarding the attributes of more participatory care (SDM) in contrast to the usual care, which reflects characteristics of a paternalist model

For peer review only - http://bmjopen.bmj.com/site/about/guidelines.xhtml

for BC screening, by establishing the relative importance of each through a DCE. In addition, the study participants were asked about their WTP for this type of healthcare.

2. METHODS

Our DCE design includes eight hypothetical profiles, referred to as choice sets, each with three characteristics—attributes—that can present different values, termed levels. For the pairing of these attributes and levels, a factorial design is considered, and it is assumed that they are independent of each other. Finally, each *choice set* contrasts with another, forming a *choice task* (21) that is presented to women to choose from as per their preference.

2.1. Definitions of attributes and levels

The Three Talk model describes and simplifies the implementation of SDM in three steps (22,23). The attributes and levels of this experiment were defined in consideration of the model, adapting it to the characteristics of the current BCSP. Table 1 describes the levels of a participatory healthcare model with characteristics of SDM (a health professional informs the patient of the risks and benefits, and a dialogue is held between the woman and the health professional, who jointly make the decision) as well as of the current model of care, which has characteristics of a paternalistic model (the risks and benefits are provided via a leaflet, the healthcare system schedules mammography according to age criteria, and the health professional makes the decision). Moreover, included in the third attribute ('Decision talk') is an additional level wherein the woman makes a decision for herself, that is, informed decision-making (IDM). Here, women receive the same information as in the first two attributes, but it is their responsibility alone to decide whether to participate in the screening.

Table 1. Attributes and levels of the options for shared decision making in breast cancer screening

Attribute	Definition	Model	Description	Levels
**1. Information talk**	How the information is obtained	Paternalist	Women are informed via a leaflet of the risks and benefits of BC screening	Leaflet
		SDM	Women are informed by a health professional of the risks and benefits of BC screening	Healthcare professional
**2. Dialogue talk**	Dialogue for mammography scheduled	Paternalist	The health system schedules mammography screening appointments in relation to age criteria	The health system schedules mammography
		SDM	A discussion of therapeutic options and women's values determines the appointment for BC screening	Women share and discuss their beliefs and values regarding mammography with a healthcare professional

For peer review only - http://bmjopen.bmj.com/site/about/guidelines.xhtml

**3. Decision talk**	Who makes the decision	Paternalist	The decision is made by the healthcare professional.	The healthcare professional makes the decision
		SDM	The decision is made through a deliberative process between the health professional and the woman	Shared decision making by the healthcare professional and the woman
		IDM	The decision is made exclusively by the woman without the support of a health professional	The woman makes the decision

SDM: Shared Decision-making; IDM: Informed Decision-making: BC: breast cancer

The current functioning of the BCSP in a Spanish region, Catalonia, was used to determine the levels for each attribute: the health system schedules a mammography appointment every 2 years for women between 50 and 69 years of age via an invitation letter; an informational leaflet is attached to the invitation letter, and the women decide whether to attend. Conversely, in relation to SDM, women inform themselves through a health professional, meet with him/her to discuss their beliefs regarding screening, and decide whether to undergo mammography.

As a result, two levels were established for the 'Information talk' and 'Dialogue talk' attributes representing the functioning of SDM; the typical model and three levels were considered for the last attribute, 'Decision talk' (Table 1).

2.2. Willingness-to-pay

There is no previous literature on WTP regarding SDM in BCSP. Therefore, the options were determined according to the average value (40€) of a consultation with a primary care doctor in Spain (24), and a variation (30€) was considered for the fraction of time devoted to SDM. Moreover, we considered other works that have analysed how to introduce SDM, which indicate that the ideal context is the primary care context with either a physician or a nurse for applicate the SDM model (25).

2.3. Experimental design

In this phase, attributes and levels are combined to create different choice sets that will be evaluated by women. The number of choice sets depends on the number of attributes and their levels. Following Reed Johnson et al.'s suggestions (26), four-choice sets were removed because they were implausible combinations and not consistent with the theory, leaving a total of eight choice sets. Those were eliminated if, without prior discussion, the professional or the professional and the woman jointly decide whether to participate, independent of whether they received the relevant information via a leaflet or a health professional.

The eight choice sets presented in pairs resulted in a maximum of 28 tasks (the action of choosing between two alternatives). Many of these could be eliminated, such as in the 16 cases in which one alternative dominated or was dominated by the other. Because SDM is considered a better option to resolve uncertainty in this context (27), it was considered a dominant choice set. The remaining 12 choice tasks were incorporated into the DCE survey,

For peer review only - http://bmjopen.bmj.com/site/about/guidelines.xhtml

as this number is below the maximum number of tasks that can be included without causing fatigued on the participants (28,29).

2.4. Piloting and refinement of the questionnaire

In March 2021, snowball sampling was carried out, and 11 women completed the questionnaire to clarify its difficulty level and ensure that the questions could be understood. This pre-pilot allowed us to identify the difficulties in understanding the elements to be evaluated. The choice tasks were reconfigured from the conventional (structured) arrangement to a descriptive one that narrated a hypothetical scenario with the three attributes in question. To better clarify structured vs descriptive surveys, the two versions are shown in Figure 1. Subsequently, a randomised pilot study was conducted on 10 women to evaluate the recruitment process and the conduct of the survey, which was of a mixed form: online system (self-applied) and telephone system (guided) (29).

Figure 1. Examples of a choice task for conventional (first version) and narrative (final version)

The survey was conducted between June and November of the same year in the Spanish and Catalan languages. The women were invited to participate, voluntarily and unpaid, through a telephone call during which they were informed of the objectives of the research project and were asked to complete a survey via telephone or online if they agreed to participate. Those who did not complete the survey within one week were reminded (Figure 2). The 12-choice task corresponding to the DCE included seven questions on sociodemographic (age, nationality, marital status, education, employment status, history of family BC, and participation in screening) and a single, multiple-choice question on WTP for BC screening care with SDM characteristics (Annex 1).

Figure 2. Net follow-up of survey responses

2.5. Participants

The study was conducted in Catalonia, Spain, with a random sample of women aged 50–60 years who participated in three BCSP: Hospital del Mar in Barcelona, Cancer Prevention and Control Program of the Catalan Institute of Oncology, and the Health Region of Lleida. This population had already been invited at least once under the national programme to undergo mammography. Therefore, it was assumed that the topic was more sensitive to their interests. Women who had been diagnosed with BC were excluded from the study.

The minimum required sample size was estimated to be 63 participants as calculated based on the empirical rule recommended by Johnson and Orme (30).

$$N>(500c)/(t*a).$$

For peer review only - http://bmjopen.bmj.com/site/about/guidelines.xhtml

N=number of respondents, t=number of choice tasks, a=number of alternatives, and c= maximum number of levels

2.6. Patient and public involvement

The patients and public were involvement on the pilot survey. So, the women gave recommendation to researchers for edit the first version and get the final survey version.

2.7 Data analysis

A mixed-effects conditional logit model was used to estimate women's preferences for different levels of SDM attributes in BC screening. This was based on Daniel McFadden's theory of discrete choice, which seeks to describe the behaviour of decision-makers in the face of a decision problem, assuming that the declared preferences of those elections are based on obtaining the maximum possible utility (31). The mixed-effects logistic regression model allows for the heterogeneity of preferences in the sample by treating the coefficients as random. It also allows multiple observations from each respondent, which is appropriate for our study, in which each woman was presented with 12 choice tasks. All models included main effects without interaction terms. A model for each subgroup was fitted to allow for comparisons according to the WTP.

All of the attribute variables were coded as dummy variables, with reference levels identified in tables with the results; further, they were specified as having a random component, assuming a normal distribution for all model coefficients. The coefficients indicate a change in preference from the reference level for each attribute (32).

When interpreting the model results, the statistical significance of the coefficients indicates whether attribute levels influence the choice set, whereas the coefficient size indicates the relative importance of one attribute level to another. We did not include an alternative-specific constant variable because our choice sets were unlabelled. Therefore, they had no utility beyond the attributes assigned to them in the experiment.

For all analyses, statistical significance was set at 0.05. All statistical analyses were performed using the R statistical software.

3. RESULTS

Of the 292 women invited to participate, 93 accepted; 27 did not reply, and 66 submitted the survey (reflecting a 70.97% response rate), with one excluded due to a history of BC. Therefore, a total sample of 65 women was obtained; the responses from two were collected by telephone, and those from the remaining 63 were collected via online forms (Figure 3). The participants' characteristics are presented in Table 2.

Figure 3. Flowchart of participants

Table 2. Characteristics of participants

For peer review only - http://bmjopen.bmj.com/site/about/guidelines.xhtml

Characteristic		
Age (years)	56.9	(1.34)
Birthplace		
Spain	57	(87.7%)
Other country	8	(12.3%)
Relationship status		
Single	17	(26.2%)
With a partner	48	(73.8%)
Education level		
Primary	18	(27.7%)
Secondary	26	(40.0%)
University	21	(32.3%)
Main occupation		
Unemployed	6	(9.23%)
Paid work	48	(73.8%)
Homemaker	11	(16.9%)
Family history of breast cancer		
No	50	(76.9%)
Yes	14	(21.5%)
Unknown	1	(1.54%)
Do you have a mammogram regularly?		
No	11	(16.9%)
Yes	54	(83.1%)

Categorical variables are summarised using frequency and percentage. Age is summarised using mean and standard deviation.

3.1. Discrete Choice Experiment results and trade–off

According to the mixed-effects conditional logit model, the weighting for each attribute was estimated using the responses to the 12 tasks. There were no missing values among the responses.

The results show that the attribute 'Decision talk' was the only one that was important to women when considering SDM in BC screening. Women preferred to make decisions alone (coefficient=2.879; 95%CI=2.297, 3.461) or to engage in SDM with healthcare professionals (coefficient=2.375; 95%CI=1.573, 3.177), in comparison to having a healthcare professional make the decision for them (Table 3). The marginal rate of substitution (MRS) between the two coefficients for who made the decision was 1.21. This meant that women perceive 21% more utility when making decisions by themselves compared to making them with a healthcare professional.

The attributes of 'Information talk' and 'Dialogue talk' showed no significant results. Therefore, women are indifferent in regard to either receiving information on the benefits

For peer review only - http://bmjopen.bmj.com/site/about/guidelines.xhtml

and risks of mammography through a leaflet or being informed by a healthcare professional (coefficient=-0.168; 95% CI=-0.665, 0.329). Neither did they show clear preference for having a meeting with a healthcare professional to discuss their beliefs for scheduling their next mammogram over a standard screening schedule (coefficient=0.145; 95% CI=-0.373, 0.663).

Table 3: Results from the mixed-effects conditional logit model

Attribute	Levels	Coefficient/SD	SE	p-value
**1. Information talk**	Leaflet	Reference category	-	-
	Healthcare professional, mean	-0.168	0.2536	0.509
	Healthcare professional, SD	1.473	0.3206	<0.001
**2. Dialogue talk**	No, health system schedules mammography	Reference category	-	-
	Yes, women share and discuss their beliefs and values about mammography with a healthcare professional, mean	0.145	0.2645	0.583
	Yes, women share and discuss their beliefs and values about mammography with a healthcare professional, SD	1.371	0.3212	<0.001
**3. Decision talk**	The healthcare professional makes the decision	Reference category	-	-

For peer review only - http://bmjopen.bmj.com/site/about/guidelines.xhtml

Shared decision making by the healthcare professional and the woman, mean	2.375	0.4093	<0.001
Shared decision making by the healthcare professional and the woman, SD	1.415	0.5208	0.007
The woman makes the decision, mean	2.879	0.2967	<0.001
The woman makes the decision, SD	1.988	0.2626	<0.001

Table 3: SD, standard deviation (of the random effects); SE, standard error.

3.2. Willingness to pay for Shared Decision making

More than half of the participants (52.3%) were willing to pay for the SDM process in BC screening; most (38.5%) would be willing to pay between 10€ and 30€, and the remainder (13.8%) would be willing to pay 40€ (Table 4).

Table 4: Women's willingness to pay for Shared Decision making in breast cancer screening

Co-payment (euros)	n	%
0	31	(47.7%)
10	6	(9.23%)
20	6	(9.23%)
30	13	(20.0%)
40	2	(3.08%)
60 or more	7	(10.8%)

Two regression models were fitted to the sample of women according to their WTP: those who showed WTP and those who did not (Table 5). The results for both subgroups were similar to those obtained for the entire sample. Thus, the attribute 'who makes the decision' was the only one that was important to women regardless of their WTP.

For peer review only - http://bmjopen.bmj.com/site/about/guidelines.xhtml

Furthermore, the results for the subgroups show the same trends in women's preferences regarding 'Information talk' and 'Dialogue talk', as in the regression model for the entire sample.

Table 5: Results from the mixed-effects conditional logit model stratified by willingness to pay

		Not willing to pay			Willing to pay		
**Attribute**	**Levels**	**Coef./SD**	**SE**	**p-value**	**Coef./SD**	**SE**	**p-value**
**1. Information talk**	Leaflet	Ref.	-	-	Ref.	-	-
	Healthcare professional, mean	-0.323	0.3706	0.383	-0.074	0.3768	0.845
	Healthcare professional, SD	1.537	0.4233	<0.001	1.434	0.4684	0.002
**2. Dialogue talk**	No, health system schedules mammography	Ref.	-	-	Ref.	-	-
	Yes, women share and discuss their beliefs and values about mammography with a healthcare professional, mean	0.187	0.3944	0.636	0.136	0.3825	0.721
	Yes, women share and discuss their beliefs and values about mammography with a healthcare professional, SD	1.599	0.4653	<0.001	1.479	0.4756	0.002
**3. Decision talk**	The healthcare professional	Ref.	-	-	Ref.	-	-

For peer review only

makes the decision						
Shared decision making by the healthcare professional and the woman, mean	2.220	0.5852	<0.001	2.826	0.6392	<0.001
Shared decision making by the healthcare professional and the woman, SD	1.449	0.5600	0.010	1.649	0.8826	0.062
The woman makes the decision, mean	2.784	0.4220	<0.001	3.006	0.4328	<0.001
The woman makes the decision, SD	1.803	0.3734	<0.001	2.548	0.4410	<0.001

Coef., coefficient; SD, standard deviation (of the random effects); SE, standard error; Ref., reference category.

For peer review only

4. DISCUSSION AND CONCLUSION

4.1. Main findings

This study analysed women's preferences for the attributes of SDM—Information talk, Dialogue talk, and Decision talk—in a BCSP through a DCE. The main result is that women prefer to make decisions themselves or together with the healthcare professional, while they do not prefer when the healthcare professional makes a decision for them. Women perceive more utility in deciding for themselves than in deciding jointly with the health professional. More than half of the women were willing to pay for screening with SDM characteristics. There was no difference in the responses obtained between women with a positive WTP and those who were not willing to pay. Regarding methodology, this study has a strong response rate (>70%) due to the close monitoring of participants' responses (33).

The importance of this study is its contribution to the scarce existing literature on the economic quantification of SDM, research on which has always been carried out in the form of qualitative studies. Moreover, most studies have focused on its applicability to public health systems; few have examined the topic from an economic perspective, and even fewer have focused on WTP using a DCE methodology.

4.2. Attribute implications

For the first attribute, ***Information talk***, the women in our study did not show a clear preference for being given an informational leaflet explaining, in a simple and balanced way,

the benefits and risks of screening compared to having this information explained by a health professional. The lack of difference between the two alternatives may be related to the fact that women do not perceive a difference in the information received. This is supported by studies such as that conducted by Longo et al. (20), who performed a DCE to determine the SDM preferences of patients in the context of chronic diseases when the doctor was receiving compared to not receiving training for the use of risk communication tools. They reported that the second most important attribute for patients was that information was easy to understand. Therefore, using a patient decision aid (PtDA) may be adequate according to women's preferences.

PtDAs deliver balanced information on the risks and benefits of BC screening, which increases informed decision-making (IDM) (34). In a recent study in Spain (35), 23% of women who received information through this route made informed decisions about their participation in screening, compared to 0.5% in the control group, who only received information attached to the invitation letter provided by the BCSP. PtDAs have the advantage of being repeatedly consulted by women to review information, as opposed to a clinical visit. However, the two strategies could act in a complementary fashion, and PtDAs could be utilised to prepare for the latter appointments to resolve doubts and review the information they contain (36).

Some institutions, such as the Public BCSP in Catalonia, have already incorporated an invitation letter for screening with a leaflet including information on the explicit need to make a decision, such as overdiagnosis, the balance of benefits and risks, and scientific uncertainties (37). Documentation is scarce in Spain, and until recently, various programmes have not provided balanced information on the benefits and adverse effects of early detection testing (38).

Improving the information provided in the programme is proposed as a strategy of PtDAs, which can be used with individuals of different literacy levels; in the local context of this study, they would ideally be used with women of higher educational levels because they are the most interested in an SDM (20), and they can understand complex information such as prevalence and absolute risks (39,40) without the need for an explanation from a health professional.

Regarding the second attribute, ***Dialogue talk***, the women in our study were indifferent in regard to the choice between attending a face-to-face appointment with a health professional to discuss their beliefs and concerns about participation in the screening and the standard approach, in which the healthcare system schedules the screening. These results may be due to the wide acceptance of screening among women because of the recognised benefits in addition to the minimisation of risks, information on which has been disseminated by persuasive preventive public health campaigns of different governments. This has led women to unquestionably participate in screening and more openly discuss the benefits and risks as well as their beliefs and fears about screening (41).

However, in a study by Longo et al. (2006) (20), the most relevant attribute for patients was their need to feel heard by their physicians, particularly because patients reported not feeling that they were heard (42). Thus, communication remains an element that should be

For peer review only - http://bmjopen.bmj.com/site/about/guidelines.xhtml

considered by healthcare professionals when they interact with their patients. Showing a receptive attitude, acceptance, empathy, and lack of judgment are the first steps in generating a trusting relationship and thus advancing to a more participatory model of health (43). Without this, it is impossible to have awareness of the beliefs of patients and, therefore, encourage them to participate in the process of SDM. This requires training in competences and communication skills (42).

Conversely, there is a high probability that women already have beliefs or fears regarding BC and want to discuss them with a health professional. In particular, the fact that having BC and surviving it is not unusual (6) means that women usually know other women already diagnosed with or treated for BC. In our study, one in five women had a first-degree relative with a history of BC. This would increase if the scope were extended to other affective bonds, such as friendships and other familiar or known people. This is of interest when considering personalisation of screening, in which women must be assisted by a healthcare professional to determine whether their risk is high, medium, or low, and recommendations must thus be made for the frequency of screening via an SDM process (44).

Regarding the third attribute, ***Decision talk***, women reject paternalistic health models, in which the healthcare professional or the system exclusively makes health decisions. This result is consistent with the current trend of empowering patients and giving them more autonomy in clinical decisions (34,37). However, this degree of autonomy may be unrealistic because of the lack of knowledge regarding adverse effects (45). For example, a recent study indicated that Spanish women have low awareness of overdiagnosis (46,47). Only 8.1% knew the meaning of overdiagnosis, although the percentage increased to 54.2% in women who received PtDAs (47).

In Longo et al.'s study (20), patients preferred to make decisions for themselves or with a healthcare professional and rejected the approach of the decision being made exclusively by a professional, which accords with our results. However, in Longo's study, this attribute was one of the least important.

The characteristics of the clinical process differ when deciding on treatment compared with when working in an early detection context, in which the target population is healthy. An option that has been well evaluated by women in previous studies (48) is to recommend the development of a targeted SDM to specific groups of women, such as those who need more information on the benefits and risks, those who have their first experience with screening, those who have lower educational levels, and those who have a high risk of being diagnosed with BC. All of these require face-to-face appointments with healthcare professionals (36,49). However, the implementation of Informed Decision making (IDM) for women with a high level of education and a low risk of being diagnosed with BC would be sufficient, and an appointment with a healthcare professional would not be essential (50). This second group can be given PtDAs with simple and clear information on screening to make the decision autonomously (34,51,52), which implies a lack of follow-up by the healthcare professional. A study is currently being conducted at the Mayo Clinic to determine whether women's discussion groups for decision making could provide a new line to support and prepare women for deciding on BC screening (53). This is based on the strategy mentioned above: the PtDAs could be delivered before the clinical meeting, and a conversation with other

For peer review only - http://bmjopen.bmj.com/site/about/guidelines.xhtml

women about the information provided there would subsequently take place. As such, the appointment between the healthcare professional and the woman would aim only at the resolution of doubts or concerns born from the PtDAs (36).

In conclusion, the results of the DCE allow us to analyse SDM's barriers and the difficulties in its applicability (40,54,55). As such, the main approaches have been developed in contexts in which decision making is particularly difficult (55). Therefore, there are still few examples of application in primary care, where decisions may be considered less difficult but not less important due to their impact on people's quality of life (51).

4.3. Willingness to pay

The subgroups separating those who were or were not willing to pay showed a preference for making decisions jointly or alone. No studies were found regarding WTP for SDM in the context of BC screening. Only one article was found for patients with BC diagnosis, but the results were focused on WTP according to the type of treatment and not on SDM characteristics (56). Another study, focused on prostate cancer, reported that participants with high WTP preferred an active to a passive participation model (57).

Regarding the monetary value of SDM, this study's findings can be compared with the results of Wilson et al.'s (57) study on patients with prostate cancer in the United States. It was determined that the men had a WTP between $25 and $50 for the implementation of SDM (57). WTP was associated with marital status (single people valued SDM more highly), the stage of SDM (those who were initiating the process of decision making were more likely to be willing to pay compared with those who were close to deciding), and the participation model (more active patients are more likely to be willing to pay compared with passive patients) (57).

In addition, Brito et al. (2020) (58) evaluated patient-reported experiences in the adult population in outpatient care through a DCE, in which SDM characteristics were included. In this case, respondents were willing to pay, on average, €16 for a doctor who provided easy-to-understand explanations in comparison to one who did not provide understandable explanations. They were also willing to pay, on average, €20 for physicians who could ask questions or raise concerns as opposed to physicians who focused on providing information. Finally, they were willing to pay, on average, €22 to physicians who involved the patient in making decisions, in contrast to those who made decisions in a one-way manner (58).

Finally, it should be considered that in Spain, screening is fully covered by the National Health System (NHS) for women aged 50–69 years. Although this is free of charge, and therefore, the ability to pay does not effectively coincide with the real payment, WTP shows the value that the society imbues by shifting away from other services for the innovation of the attention model on appointment (59) and, in the case of innovation being implemented in the NHS, may imply a burden on the health system and co-payment or direct payment from women (14). In the latter case, the out-of-pocket cost to provide a screening is one of the determining elements for adherence to SDM in BCSP (60).

For peer review only - http://bmjopen.bmj.com/site/about/guidelines.xhtml

Future research could include randomised clinical trials, in which women would experience healthcare with all of the attributes that SDM elicits; their preferences, perceived satisfaction, and profit could be analysed in comparison to regular attendance, and the costs that the implementation of SDM would entail in the screening of BC for the NHS could be identified.

4.4. Limitations

The main limitation of the study, which forced us to change the design of the questions, was the difficulty women experienced in comparing hypothetical profiles; we detected difficulties in understanding the differences between the profiles proposed in each choice task. In addition, their ability to detect differences among the 12 questions in the DCE survey was limited. This may also be due to women's lack of familiarity with participatory health characteristics due to the current BCSP model (exposure bias) as well as that the decisions regarding screening seem to reflect a social consensus on the need for screening and that its utility is not questioned. To eliminate exposure bias, future randomised studies should be conducted in which women can experience both models of care (paternalist and SDM), and then their preferences and perceived satisfaction can be compared to their experience of regular attendance to identify the costs that the implementation of SDM would entail in the BCSP. Considering that 'who makes the decision' is the most important factor for women, strategies could be proposed to ensure that, through discussion groups, women can make an informed decision, as contexts of prevention, in contrast to those of treatment, may be more acceptable to women (53). These new procedures are currently being introduced in studies being conducted at the Mayo Clinic, among others, which are aimed at facilitating more participatory and more informed decision making (53).

Finally, it is difficult to precisely determine ability to pay because in Spain, BC screening is under the purview of the NHS. Therefore, the services are free at the time they are provided, and the population assumes that this is a consolidated health benefit and does not consider it to be something that they should pay for, particularly because screening for other types of cancer (e.g., colorectal) is currently being introduced and heavily promoted by the NHS.

4.5. Conclusion

This is the first study examining women's preferences regarding SDM in relation to BC screening. Women were shown to reject paternalistic health models in the context of BCSP and to prefer models with IDM or SDM. More than half of the participants were willing to pay for active involvement in their health decisions.

4.6. Practice Implications

The change from a paternalistic model to a participatory model of person-centred medicine would require restructuring of the BCSP so that women can make informed decisions for themselves or engage in SDM together with a health professional.

5. CONTRIBUTION STATEMENT

For peer review only - http://bmjopen.bmj.com/site/about/guidelines.xhtml

María José Hernández-Leal, María José Pérez-Lacasta, and **Misericòrdia Carles-Lavila**: Conceptualisation, Methodology, Formal analysis, Investigation, Project administration, Supervision, Visualisation, Writing- original draft. **Angels Cardona** and **Núria Codern-Bové**: Data curation, Formal analysis, Writing- review and editing. **Carmen Vidal-Lancis**: Resources, Writing- review and editing. **Montserrat Rué**: Resources, Formal analysis, Writing- review and editing. **Carles Forné**: Software, Formal analysis, Writing- review and editing.

6. COMPETING INTERESTS

The authors declare that they have no conflict of interest.

7. FUNDING INFORMATION

Financial support for this study was provided entirely by a grant from Instituto de Salud Carlos III through the project PI18/00773 (co-funded by European Regional Development Fund) and the European Union's Horizon 2020 research and innovation programme under the Marie Skłodowska-Curie grant agreement No. 713679 from the Universitat Rovira i Virgili (URV). The funding agreement ensures the authors' independence in designing the study, interpreting data, and writing and publishing the report.

8. DATA SHARING STATEMENT

All data relevant to the study are included in the manuscript and tables or uploaded as supplementary information.

9. ETHICAL APPROVAL STATEMENT

This research was approved by the Medicinal Product Research Ethics Committee (CEIm) of the Institut d'Investigació Sanitària Pere Virgili (Pere Virgili Health Research Institute) Ref. CEIM: 175/2019. Informed consent, which stated that the participants accepted the conditions of participation upon agreeing to respond to the questionnaire, was obtained. These conditions specified that the responses were confidential and would be used only for this research.

10. REFERENCES

1. Kerlikowske K, Grady D, Rubin SM, Sandrock C, Ernster VL. Efficacy of screening mammography. A meta-analysis. JAMA. 1995;273(2):149-154. PMID: 7799496
2. Walter LC, Lewis CL, Barton MB. Screening for colorectal, breast, and cervical cancer in the elderly: a review of the evidence. Am J Med. 2005;118(10):1078-1086. doi: 10.1016/j.amjmed.2005.01.063.
3. Sung H, Ferlay J, Siegel RL, Laversanne M, Soerjomataram I, Jemal A, et al. Global cancer statistics 2020: GLOBOCAN estimates of incidence and mortality worldwide for 36 cancers in 185 countries. CA Cancer J Clin. 2021;71(3):209-249. doi:https://doi.org/10.3322/caac.21660.
4. Institut Català d'Oncologia, de Càncer de Mama DP. [Early breast cancer detection]. 2020. Available from: http://gencat.cat/ca/el-

For peer review only

cancer/programes_de_deteccio_precoc/programa_de_deteccio_precoc_de_cancer_de_mama/index.html.

5. Ministerio de Sanidad, Servicios Sociales e Igualdad, Instituto Nacional de Estadística. Encuesta europea de Salud en España EESE 2020 [European survey of health in Spain EESE 2020]. Madrid: ministerio de sanidad. 2020. Available from: https://www.sanidad.gob.es/estadEstudios/estadisticas/EncuestaEuropea/EncuestaEuropea2020/EESE2020_inf_evol_princip_result.pdf.
6. Ministerio de Sanidad, Servicios Sociales e Igualdad, Instituto Nacional de Estadística. Encuesta europea de salud en España EESE 2017 [European survey of health in Spain EESE 2017]. 2010. Disponible enhttps:. Available from: http://www.mscbs.gob.es/estadEstudios/estadisticas/encuestaNacional/encuestaNac2017/DETECCION_PRECOZ_CANCER.pdf.
7. Jørgensen KJ, Klahn A, Gøtzsche PC. Are benefits and harms in mammography screening given equal attention in scientific articles? A cross-sectional study. BMC Med. 2007;5:12. doi: 10.1186/1741-7015-5-12.
8. Marmot MG, Altman DG, Cameron DA, Dewar JA, Thompson SG, Wilcox M. The benefits and harms of breast cancer screening: an independent review. Br J Cancer. 2013;108(11):2205-2240. doi: 10.1038/bjc.2013.177.
9. Houssami N. Overdiagnosis of breast cancer in population screening: does it make breast screening worthless? Cancer Biol Med. 2017;14(1):1-8. doi: 10.20892/j.issn.2095-3941.2016.0050.
10. Hargraves IG, Montori VM, Brito JP, Kunneman M, Shaw K, LaVecchia C, Wilson M, Walker L, Thorsteinsdottir B. Purposeful SDM: A problem-based approach to caring for patients with shared decision making. Patient Educ Couns 2019102. 2019;102(10):1786-1792. doi: 10.1016/j.pec.2019.07.020. PMID: 31353170; PMCID: PMC6717012.
11. Barr PJ, Scholl I, Bravo P, Faber MJ, Elwyn G, McAllister M. Assessment of patient empowerment--a systematic review of measures. PLoS One. 2015 May 13;10(5):e0126553. doi: 10.1371/journal.pone.0126553. PMID: 25970618; PMCID: PMC4430483.
12. Elwyn G, Edwards A, Kinnersley P, Grol R. Shared decision making and the concept of equipoise: the competences of involving patients in healthcare choice. Br J Gen Pract. 2000;50:892-897. Available from: https://bjgp.org/content/bjgp/50/460/892.full.pdf.
13. Elwyn G, Frosch DL, Kobrin S. Implementing shared decision-making: consider all the consequences. Implement Sci. 2016;11:114. doi: 10.1186/s13012-016-0480-9. PMID: 27502770; PMCID: PMC4977650.
14. Walsh T, Barr PJ, Thompson R, Ozanne E, O'Neill C, Elwyn G. Undetermined impact of patient decision support interventions on healthcare costs and savings: systematic review. BMJ. 2014;348:g188. doi: 10.1136/bmj.g188. PMID: 24458654; PMCID: PMC3900320.
15. Katz SJ, Hawley S. The value of sharing treatment decision making with patients: expecting too much? JAMA. 2013;310(15):1559-1560. doi: 10.1001/jama.2013.278944. PMID: 24061082.
16. Oshima Lee E, Emanuel EJ. Shared decision making to improve care and reduce costs. N Engl J Med. 2013;368(1):6-8. doi: 10.1056/NEJMp1209500. PMID: 23281971.
17. Ryan M, Gerard K. Using discrete choice experiments to value health care programmes: current practice and future research reflections. Appl Health Econ Health Policy. 2003;2(1):55-64. PMID: 14619274.

For peer review only - http://bmjopen.bmj.com/site/about/guidelines.xhtml

18. Ryan M, Bate A, Eastmond CJ, Ludbrook A. Use of discrete choice experiments to elicit preferences. Qual Health Care. 2001;10;Suppl 1:i55-60. doi: 10.1136/qhc.0100055: 11533440; PMCID: PMC1765744.
19. Taguchi R, Yamazaki Y, Nakayama K. Eliciting preferences for mammography: using a discrete choice experiment approach. Nihon koshu eisei zasshi. 2010;57(2):83-94. PMID: 20415238.
20. Longo MF, Cohen DR, Hood K, Edwards A, Robling M, Elwyn G, Russell IT. Involving patients in primary care consultations: assessing preferences using discrete choice experiments. Br J Gen Pract. 2006 ;56(522):35-42. PMID: 16438813; PMCID: PMC1821413.
21. Norman R, Craig BM, Hansen P, Jonker MF, Rose J, Street DJ, Mulhern B. Issues in the design of discrete choice experiments. Patient. 2019;12(3):281-285. doi: 10.1007/s40271-018-0346-0. PMID: 30446958.
22. Elwyn G, Frosch D, Thomson R, Joseph-Williams N, Lloyd A, Kinnersley P, Cording E, Tomson D, Dodd C, Rollnick S, Edwards A, Barry M. Shared decision making: a model for clinical practice. J Gen Intern Med. 2012 Oct;27(10):1361-7. doi: 10.1007/s11606-012-2077-6. Epub 2012 May 23. PMID: 22618581; PMCID: PMC3445676.
23. Elwyn G, Durand MA, Song J, Aarts J, Barr PJ, Berger Z, Cochran N, Frosch D, Galasiński D, Gulbrandsen P, Han PKJ, Härter M, Kinnersley P, Lloyd A, Mishra M, Perestelo-Perez L, Scholl I, Tomori K, Trevena L, Witteman HO, Van der Weijden T. A three-talk model for shared decision making: multistage consultation process. BMJ. 2017;359:j4891. doi: 10.1136/bmj.j4891. PMID: 29109079; PMCID: PMC5683042.
24. Department of Health. Approval of public prices of the Catalan Health Service SLT/30/2013. Num 6323 Diari Oficial de la Generalitat de Catalunya [Aprobación de precios públicos del Servicio Catalán de Salud SLT/30/2013. Num 6323 Diari Oficial de la Generalitat de Catalunya]. 2013, 6323; 10695-10862. Available from: https://www.uch.cat/documents/preus-publics-catsalut.pdf
25. Laza-Vásquez C, Codern-Bové N, Cardona-Cardona À, Hernández-Leal MJ, Pérez-Lacasta MJ, Carles-Lavila M, Rué M; DECIDO group. Views of health professionals on risk-based breast cancer screening and its implementation in the Spanish National Health System: A qualitative discussion group study. PLoS One. 2022 Feb 4;17(2):e0263788. doi: 10.1371/journal.pone.0263788. PMID: 35120169; PMCID: PMC8815913.Reed Johnson F, Lancsar E, Marshall D, Kilambi V, Mühlbacher A, Regier DA, Bresnahan BW, Kanninen B, Bridges JF. Constructing experimental designs for discrete-choice experiments: report of the ISPOR Conjoint Analysis Experimental Design Good Research Practices Task Force. Value Health. 2013;16(1):3-13. doi: 10.1016/j.jval.2012.08.2223. PMID: 23337210
26. Reed Johnson F, Lancsar E, Marshall D, Kilambi V, Mühlbacher A, Regier DA, Bresnahan BW, Kanninen B, Bridges JF. Constructing experimental designs for discrete-choice experiments: report of the ISPOR Conjoint Analysis Experimental Design Good Research Practices Task Force. Value Health. 2013;16(1):3-13. doi: 10.1016/j.jval.2012.08.2223. PMID: 23337210.
27. Hernández-Leal MJ, Carles-Lavila M, Pérez-Lacasta M. La participación de los profesionales de la salud en la Toma de Decisiones Compartida en el cribado de cáncer de mama: manual de apoyo para implementación de la TDC. [The participation of health professionals in shared decision-making in breast cancer screening: A support manual

For peer review only - http://bmjopen.bmj.com/site/about/guidelines.xhtml

for the implementation of SDM]. Spain: María José Hernández editor. 2021. Available from: https://www.researchgate.net/publication/358277309_The_Participation_of_Health_Professionals_in_Shared_Decision-Making_on_Breast_Cancer_Screening
28. Ryan M, Bate A. Testing the assumptions of rationality, continuity and symmetry when applying discrete choice experiments in health care. Appl Econ Lett. 2001;8(1):59-63. doi: 10.1080/135048501750041312.
29. Mariel P, Hoyos D, Meyerhoff J, Czajkowski M, Dekker T, Glenk K, Jacobsen JB, Liebe U, Olsen SB, Sagebiel J, Thiene M. 2020. Environmental valuation with discrete choice experiments: guidance on design, implementation and data analysis. Springer;2020, pp 54-58. Available from: http://eprints.whiterose.ac.uk/168622/.
30. de Bekker-Grob EW, Donkers B, Jonker MF, Stolk EA. Sample size requirements for discrete-choice experiments in healthcare: a practical guide. Patient. 2015;8(5):373-384. doi: 10.1007/s40271-015-0118-z. PMID: 25726010; PMCID: PMC4575371.
31. McFadden D. Conditional logit analysis of qualitative choice behavior. 1973. Zarembka, P. Frontiers in econometrics. Academic Press: New York, pp 105-142.
32. Herrera C. Valoración y modelado de las preferencias en calidad de vida relacionadas con la salud de menores [Valuation and modeling of health related quality of life preferences of younger population]. 2020. Available from: https://riull.ull.es/xmlui/bitstream/handle/915/20075/Valoracion%20y%20modelado%20de%20las%20preferencias%20en%20calidad%20de%20vida%20relacionadas%20con%20la%20salud%20de%20menores..pdf?sequence=1.
33. Watson V, Becker F, de Bekker-Grob E. Discrete Choice Experiment Response Rates: A Meta-analysis. Health Econ. 2017 Jun;26(6):810-817. doi: 10.1002/hec.3354. Epub 2016 Apr 27. PMID: 27122445.
34. Hersch JK, Nickel BL, Ghanouni A, Jansen J, McCaffery KJ. Improving communication about cancer screening: moving towards informed decision making. Public Health Res Pract. 2017;27(2):2731728. doi: 10.17061/phrp2731728. PMID: 28765861.
35. Pérez-Lacasta MJ, Martínez-Alonso M, Garcia M, Sala M, Perestelo-Pérez L, Vidal C, et al. Effect of information about the benefits and harms of mammography on women's decision making: the InforMa randomised controlled trial. PLOS ONE. 2019;14(3):e0214057. doi: 10.1371/journal.pone.0214057.
36. Maschke A, Paasche-Orlow MK, Kressin NR, Schonberg MA, Battaglia TA, Gunn CM. Discussions of potential mammography benefits and harms among patients with limited health literacy and providers: 'oh, there are harms?' J Health Commun. 2020;25(12):951-961. doi: 10.1080/10810730.2020.1845256. PMID: 33455518; PMCID: PMC8062298.
37. Forbes LJ, Ramirez AJ, Expert group on Information about Breast Screening. Offering informed choice about breast screening. J Med Screen. 2014;21(4):194-200. doi: 10.1177/0969141314555350. PMID: 25312639.
38. Ballesteros-Peña S, Gavilán-Moral E. Contenido de los documentos informativos dirigidos a las mujeres sobre el cribado de cáncer de mama en España [Content of official addressed to women informative documents about breast cancer screening in Spain]. Rev Esp Salud Publica. 2018;92:e201810076. Spanish. PMID: 30361472.
39. Millaruelo J, Martín F. Toma de decisiones compartidas con el paciente [Shared decision-making with the patient]. Rev Clin Elect en Aten Pri. 2009. Available from.

For peer review only - http://bmjopen.bmj.com/site/about/guidelines.xhtml

Available from: https://ddd.uab.cat/pub/rceap/rceap_a2009m5n17/rceap_a2009m5n17a9.pdf
40. Hernández-Leal MJ, Pérez-Lacasta MJ, Feijoo-Cid M, Ramos-García V, Carles-Lavila M. Healthcare professionals' behaviour regarding the implementation of shared decision-making in screening programmes: A systematic review. Patient Educ Couns. 2021 Aug;104(8):1933-1944. doi: 10.1016/j.pec.2021.01.032. PMID: 33581968.
41. Hersch J, Jansen J, McCaffery K. Decision-making about mammographic screening: pursuing informed choice. Climacteric. 2018;21(3):209-213, doi: 10.1080/13697137.2017.1406912
42. Holmström I, Rosenqvist U. A change of the physicians understanding of the encounter parallels competence development. Patient Educ Couns. 2001;42(3):271-278. doi: 10.1016/s0738-3991(00)00132-4. PMID: 11164326.
43. Vargas I, Guerrero S, Hernández-Leal M, Campos R. La Confianza en la relación profesional de la salud y paciente [Confidence in the health professional and patient relation] Rev Cubana Salud Pública. 2020;26(3). Available from: http://www.revsaludpublica.sld.cu/index.php/spu/article/view/1575/1634.
44. Sala M, Domingo L, Rué M, Comas M, Torá-Rocamora I, Macià F, Castells X. Investigación en cribado de cáncer de mama: camino hacia estrategias personalizadas y decisiones compartidas [Research on breast cancer screening: toward personalized strategies and shared decision-making]. Revista de Senología y Patología Mamaria. 2014;27(4):176-182. doi: 10.1016/j.senol.2014.07.007.
45. Mathioudakis AG, Salakari M, Pylkkanen L, Saz-Parkinson Z, Bramesfeld A, Deandrea S, Lerda D, Neamtiu L, Pardo-Hernandez H, Solà I, Alonso-Coello P. Systematic review on women's values and preferences concerning breast cancer screening and diagnostic services. Psycho-Oncology. 2019;28(5):939-947. doi: 10.1002/pon.5041. PMID: 30812068; PMCID: PMC6594004.
46. Baena-Cañada JM, Rosado-Varela P, Expósito-Álvarez I, González-Guerrero M, Nieto-Vera J, Benítez-Rodríguez E. Women's perceptions of breast cancer screening. Spanish screening programme survey. Breast. 2014;23(6):883-888. doi: 10.1016/j.breast.2014.09.010. PMID: 25456103.
47. Toledo-Chávarri A, Rué M, Codern-Bové N, Carles-Lavila M, Perestelo-Pérez L, Pérez-Lacasta MJ, et al. A qualitative study on a decision aid for breast cancer screening: views from women and health professionals. Eur J Cancer Care (Engl). 2017;26(3):1-11. doi: 10.1111/ecc.12660.
48. Rainey L, van der Waal D, Donnelly LS, Southworth J, French DP, Evans DG, Broeders MJM. Women's health behaviour change after receiving breast cancer risk estimates with tailored screening and prevention recommendations. BMC Cancer. 2022;22(1):69. doi: 10.1186/s12885-022-09174-3. PMID: 35033023.
49. Esquivel-Sada D, Lévesque E, Hagan J, Knoppers BM, Simard J. Envisioning implementation of a personalized approach in breast cancer screening programs: stakeholder perspectives. Healthc Policy. 2019;15(2):39-54. doi: 10.12927/hcpol.2019.26072. PMID: 32077844; PMCID: PMC7020798.
50. Rimer BK, Briss PA, Zeller PK, Chan EC, Woolf SH. Informed decision making: what is its role in cancer screening? Cancer. 2004;101(5);Suppl:1214-1228. doi: 10.1002/cncr.20512. PMID: 15316908.
51. Caverly TJ, Hayward RA. Dealing with the lack of time for detailed shared decision-making in primary care: everyday shared decision-making. J Gen Intern Med.

For peer review only - http://bmjopen.bmj.com/site/about/guidelines.xhtml

2020;35(10):3045-3049. doi: 10.1007/s11606-020-06043-2; PMID: 32779137; PMCID: PMC7572954.
52. Stacey D, Légaré F, Lewis K, Barry MJ, Bennett CL, Eden KB, Holmes-Rovner M, Llewellyn-Thomas H, Lyddiatt A, Thomson R, Trevena L. Decision aids for people facing health treatment or screening decisions. Cochrane Database Syst Rev. 2017;4(4):CD001431. doi: 10.1002/14651858.CD001431.pub5. PMID: 28402085; PMCID: PMC6478132.
53. Hernández-Leal M, Montori V. 2021. Discussion groups for decision-making on breast cancer screening [research proyect]. Mayo Clinic.
54. Légaré F, Ratté S, Gravel K, Graham ID. Barriers and facilitators to implementing shared decision-making in clinical practice: update of a systematic review of health professionals' perceptions. Patient Educ Couns. 2008;73(3):526-535. doi: 10.1016/j.pec.2008.07.018.
55. Légaré F, Witteman HO. Shared decision making: examining key elements and barriers to adoption into routine clinical practice. Health Aff (Millwood). 2013;32(2):276-284. doi: 10.1377/hlthaff.2012.1078.
56. Johnson P, Bancroft T, Barron R, Legg J, Li X, Watson H, Naeim A, Watkins A, Marshall DA. Discrete choice experiment to estimate breast cancer patients' preferences and willingness to pay for prophylactic granulocyte colony-stimulating factors. Value Health. 2014;17(4):380-389. doi: 10.1016/j.jval.2014.01.002. PMID: 24968998.
57. Wilson LS, Blonquist TM, Hong F, Halpenny B, Wolpin S, Chang P, Filson CP, Master VA, Sanda MG, Chien GW, Jones RA, Krupski TL, Berry DL. Assigning value to preparation for prostate cancer decision making: a willingness to pay analysis. BMC Med Inform Decis Mak. 2019;19(1):6. doi: 10.1186/s12911-018-0725-4. PMID: 30626400; PMCID: PMC6327504.
58. Brito Fernandes Ó, Péntek M, Kringos D, Klazinga N, Gulácsi L, Baji P. Eliciting preferences for outpatient care experiences in Hungary: A discrete choice experiment with a national representative sample. PLoS One. 2020;15(7):e0235165. doi: 10.1371/journal.pone.0235165. PMID: 32735588; PMCID: PMC7394384.
59. Puig-Junoy J, Pinto-Prades JL, Ortún-Rubio V. El análisis coste-beneficio en sanidad [Cost-benefit analysis in health care]. Aten Primaria. 2001;27(6):422-7. Spanish. doi: 10.1016/s0212-6567(01)78825-7. PMID: 11334581; PMCID: PMC7677961.
60. Mansfield C, Tangka FK, Ekwueme DU, Smith JL, Guy GP Jr, Li C, Hauber AB. Stated preference for cancer screening: A systematic review of the literature, 1990-2013. Prev Chronic Dis. 2016;13:E27. doi: 10.5888/pcd13.150433. PMID: 26916898; PMCID: PMC4768876.

11. FIGURE LEGENDS

Figure 1. Examples of a choice task for conventional (first version) and narrative (final version)

Figure 2. Net follow-up of survey responses

Figure 3. Flowchart of participants

For peer review only - http://bmjopen.bmj.com/site/about/guidelines.xhtml

Figure 1. Examples of a choice task for conventional (first version) and narrative (final version)

Conventional choice task (first version)

Characteristics	Option A	Option B
Information	I was informed by leaflet	I was informed by a health professional
Communication	I discuss mammography with a healthcare professional	The health system schedules my mammography
Make a decision	I make the decision of whether to have a mammogram	I make the decision of whether to have a mammogram
Choose one option		

→ Pre-test

Narrative choice task (final version)

Option A	Option B
I receive **information about the benefits and risks in a leaflet**, and I also **talk with a healthcare professional** about my fears or beliefs about mammography. Finally, **I make the decision** to attend -or not- to mammography.	I am **informed by a health professional** about the benefits and risks; my **mammography is scheduled by the health system**, so I do not manifest my fears, beliefs or preferences about the screening to a health professional. Finally, **I make the decision** to attend -or not- to mammography.

Figure 1. Examples of a choice task for conventional (first version) and narrative (final version)

254x97mm (144 x 144 DPI)

Figure 2. Net follow-up of survey responses

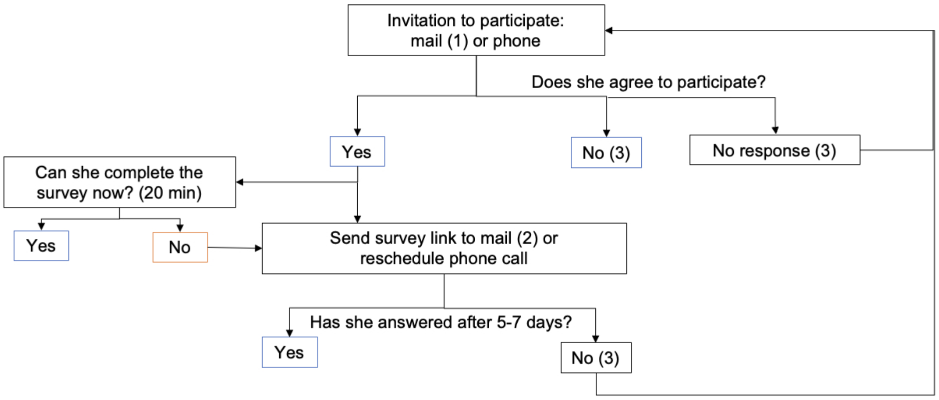

1) Attach ethics committee approval
2) Spanish and Catalan survey link
3) If the participants do not respond to the invitation or survey after three attempts, delete them from the database

Figure 2. Net follow-up of survey responses

226x138mm (144 x 144 DPI)

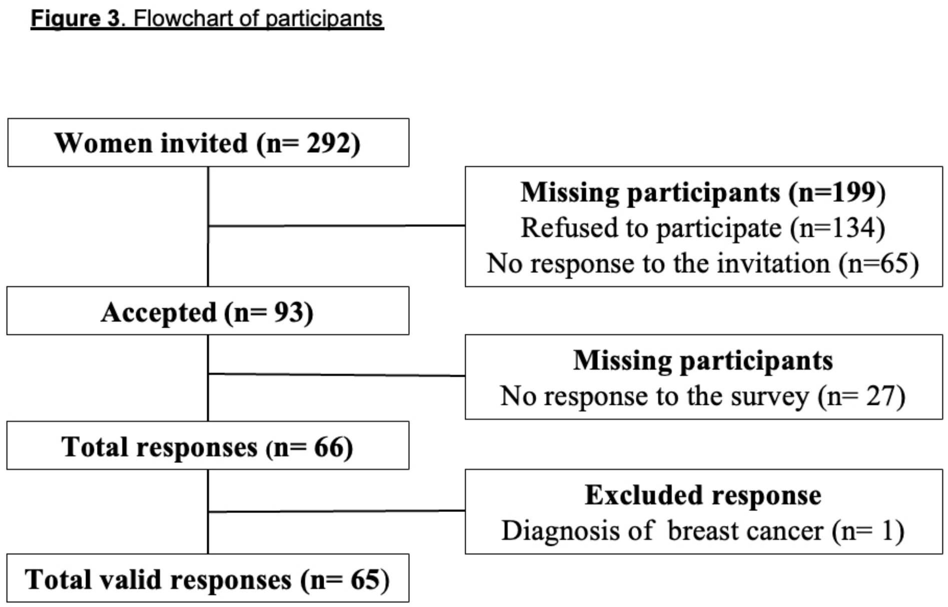

Figure 3. Flowchart of participants

168x107mm (144 x 144 DPI)

“Women’s Preferences for Breast Cancer Screening"

We appreciate your collaboration in the ProShare research project "*The collaboration of healthcare professionals to include Shared Decision Making in the breast cancer screening program*" (P18/00773), led by Dr. Misericòrdia Carles. This project is supported and funded by the Carlos III Institute of Health and co-financed by the European Regional Development Fund (ERDF) “A way of making Europe” y “European Union's Horizon 2020 research and innovation program under the Marie Skłodowska-Curie grant agreement” No. 713679 and from the Universitat Rovira i Virgili (URV).

The objective of your participation is to identify women’s preferences and determine the cost-benefit of a new care model for the breast cancer screening program. So, we present you, in two hypothetical options and you must choose only one of them according to your preferences. Consider that there are no good or bad answers.

Your answers will be confidential and will only be used for research purposes. Answer the following questions truthfully, considering that your identity will never be exposed.

For peer review only

Shared Decision Making in Health

1. Socio-demographic characteristics
a. Name: __________

b. Age (years):______________

c. Birthplace (mark with an X)

	Spain
	Other country

d. Civil status (mark with an X)

	Single
	With partner

e. Education level (mark with an X)

	Primary
	Secondary
	University

f. Main occupation (mark with an X)

	Paid work
	Household chores
	Unemployed

g. Family history of breast cancer (mark with an X)

	Yes
	No
	Unknown

h. Do you have your mammogram regularly? (mark with an X)

	Yes
	No

2. Experimental design

The next 12 questions will be asked in consultation on three characteristics of the breast cancer screening program:

For peer review only

1. **How do I receive the information?** You will find two possibilities; in one of them you are informed about the benefits and risks of mammography through a **leaflet** or by **health professional**.

2. Dialogue about my preferences? You'll find two possibilities; in one, you **meet with a healthcare professional** to discuss your preferences, fears, or concerns about mammography. And on the other hand, **the health system** simply programs its mammography according to its age.

3. Who makes the decision? You will find three options about who makes the decision for attended -or no- to mammography: the **health professional**, **the woman** or **jointly**. Independent if later this is concrete the mammography

In each question you must choose the option you prefer. Each option includes the three features above.

For example:
Question 0: Mark with an "X" the option you prefer

Option A	Option B
I am informed by a **healthcare professional** about the benefits and risks, **I also express my fears**, beliefs, or preferences of mammography. Finally, **I myself made the decision** attend -or not- mammography.	I am informed by a **healthcare professional** about the benefits and risks, **I also express my fears**, beliefs or preferences of mammography. Finally, **we jointly made the decision** attend -or not- to mammography.

We are aware that in some questions the options may be very similar to you, yet there is always some different characteristics There are no correct or incorrect answers, you should select only based on your personal appreciation.

SURVEY

1: Mark with an "X" the option you prefer

Option A	Option B

**The healthcare professionals inform** me about the benefits and risks; **I also tell them about my fears**, beliefs, or preferences for mammography. Finally, **the healthcare professional makes the decision** attend -or not- to the mammography.	**The healthcare professionals inform me** about the benefits and risks; **I also tell them about my fears**, beliefs, or preferences for mammography. Finally, **I myself made the decision** attend -or not- to the mammography.

2.Mark with an "X" the option you prefer

Option A	Option B
I receive information about the benefits and risks through a **leaflet**; **I also share with a healthcare professional my fears** or beliefs about mammography. Finally, **we jointly made the decision** attend -or not- to the mammography.	**I am informed by a healthcare professional** about the benefits and risks; **I also express my fears, beliefs,** or preferences of mammography. Finally, **I myself made the decision** attend -or not- to the mammography.

3. Mark with an "X" the option you prefer

Option A	Option B
I receive information about the benefits and risks through **a leaflet**; **I also tell a healthcare professional my fears** or beliefs about mammography. Finally, **the professional makes the decision** attend -or not- to the mammography.	**The healthcare professionals inform** me about the benefits and risks; **I also tell them about my fears**, beliefs, or preferences for mammography. Finally, **I myself made the decision** attend -or not- to the mammography.

4. Mark with an "X" the option you prefer

Option A	Option B
**I am informed by a healthcare professional** about the benefits and risks, but **without the possibility of expressing my fears** or beliefs about mammography as it is defined by the health program. Finally, **I myself made the decision** attend -or not- to the mammography.	**The healthcare professional informs me** about the benefits and risks**; I also tell them about my fears**, beliefs, or preferences for mammography. Finally, **the healthcare professional makes the decision** attend -or not- to the mammography.

5. Mark with an "X" the option you prefer

Option A	Option B
I receive information about the benefits and risks through **a leaflet**; I also **tell a**	**The healthcare professional informs me** about the benefits and risks; **I also**

For peer review only

**healthcare professional my fears** or beliefs about mammography. Finally, **we jointly made the decision** attend -or not- to the mammography.	**tell them about my fears**, beliefs, or preferences for mammography. Finally, the **healthcare professional makes the decision** attend -or not- to the mammography.

6. Mark with an "X" the option you prefer

Option A	Option B
I receive information about the benefits and risks through **a leaflet**; **I also tell a healthcare professional my fears** or beliefs about mammography. Finally, **I myself made the decision** attend -or not- to the mammography.	**The healthcare professional informs me** about the benefits and risks; **I also tell them about my fears**, beliefs, or preferences for mammography. Finally, **the healthcare professional makes the decision** attend -or not- to the mammography.

7. Mark with an "X" the option you prefer

Option A	Option B
I receive information about the benefits and risks through **a leaflet**; **my mammography programs the health system**, so I do not manifest my fears, beliefs, or preferences about mammography to a health professional. Finally, **I myself made the decision** attend -or not- to the mammography.	**The healthcare professional informs me** about the benefits and risks; **I also tell them about my fears**, beliefs, or preferences for mammography. Finally, the **healthcare professional makes the decision** attend -or not- to the mammography.

8. Mark with an "X" the option you prefer

Option A	Option B
I receive information about the benefits and risks through **a leaflet**; **I also tell a healthcare professional my fears** or beliefs about mammography. Finally, **we jointly made the decision** attend -or not- to the mammography.	**I am informed by a health professional** about the benefits and risks; **my mammography programs the health system**, so I do not manifest my fears, beliefs, or preferences about mammography to a health professional. Finally, **I myself made the decision** attend -or not- to the mammography.

9. Mark with an "X" the option you prefer

Option A	Option B
I receive information about the benefits and risks in a **leaflet; I also share my fears** or beliefs about mammography with a health professional. Finally, **I myself**	**I am informed by a health professional** about the benefits and risks; my mammography programs the health system, **so I do not manifest my fears**, beliefs or preferences about

**made the decision** attend -or not- to the mammography.	mammography with a health professional. Finally, **I myself made the decision** attend -or not- to the mammography.

10. Mark with an "X" the option you prefer

Option A	Option B
I receive information about the benefits and risks in a **leaflet**; **I also share my fears** or beliefs about mammography with a health professional. Finally, **the professional makes the decision** attend -or not- to the mammography.	**I am informed by a health professional** about the benefits and risks; however, **I do not manifest my fears**, beliefs or preferences about mammography as it programs the health system. Finally, **I myself made the decision** attend -or not- to the mammography

11. Mark with an "X" the option you prefer

Option A	Option B
I receive information about the benefits and risks in a **leaflet**; **I also share my fears** or beliefs about mammography with a health professional. Finally, **the professional makes the decision** attend -or not- to the mammography	I receive information about the benefits and risks in a **leaflet**; **I also share my fear**s or beliefs about mammography with a health professional. Finally, **I myself made the decision** attend -or not- to the mammography

12. Mark with an "X" the option you prefer

Option A	Option B
I receive information about the benefits and risks a **leaflet**; **my mammography programs the health system**, so I do not manifest my fears, beliefs or preferences about mammography with a health professional. Finally, **I myself made the decision** attend -or not- to the mammography	I receive information about the benefits and risks in a **leaflet**; **I also share my fears** or beliefs about mammography with a health professional. Finally**, the professional makes the decision** attend -or not- to the mammography

3. Cost of health

The breast cancer screening is currently not paid to women, as it is covered by the public health service. Regardless of the current cost, if you had the option of attending an appointment with a healthcare professional to inform you about the benefits and risks of mammography, discuss with him/her your preferences and concerns and jointly make the decision to attend -or not to mammography **How much money would you be willing to pay?**

	0 euros

For peer review only

	10 euros
	20 euros
	30 euros
	40 euros
	60 euros or more

Thank you so much for the time you took to answer this survey to improve women's care in breast cancer screening.

ProShare

For peer review only

Women's Preference to Apply Shared Decision-Making in Breast Cancer Screening: A Discrete Choice Experiment

STROBE Statement—Checklist of items that should be included in reports of ***cross-sectional studies***

	Item No	Recommendation	Page No
**Title and abstract**	1	(*a*) Indicate the study's design with a commonly used term in the title or the abstract	1
		(*b*) Provide in the abstract an informative and balanced summary of what was done and what was found	2
**Introduction**			
Background/rationale	2	Explain the scientific background and rationale for the investigation being reported	3
Objectives	3	State specific objectives, including any prespecified hypotheses	3
**Methods**			
Study design	4	Present key elements of study design early in the paper	4
Setting	5	Describe the setting, locations, and relevant dates, including periods of recruitment, exposure, follow-up, and data collection	6
Participants	6	(*a*) Give the eligibility criteria, and the sources and methods of selection of participants	6
Variables	7	Clearly define all outcomes, exposures, predictors, potential confounders, and effect modifiers. Give diagnostic criteria, if applicable	5
Data sources/ measurement	8*	For each variable of interest, give sources of data and details of methods of assessment (measurement). Describe comparability of assessment methods if there is more than one group	*5*
Bias	9	Describe any efforts to address potential sources of bias	5
Study size	10	Explain how the study size was arrived at	6
Quantitative variables	11	Explain how quantitative variables were handled in the analyses. If applicable, describe which groupings were chosen and why	6
Statistical methods	12	(*a*) Describe all statistical methods, including those used to control for confounding	6
		(*b*) Describe any methods used to examine subgroups and interactions	6
		(*c*) Explain how missing data were addressed	NA
		(*d*) If applicable, describe analytical methods taking account of sampling strategy	6
		(*e*) Describe any sensitivity analyses	6
**Results**			
Participants	13*	(a) Report numbers of individuals at each stage of study—eg numbers potentially eligible, examined for eligibility, confirmed eligible, included in the study, completing follow-up, and analysed	7
		(b) Give reasons for non-participation at each stage	7
		(c) Consider use of a flow diagram	7
Descriptive data	14*	(a) Give characteristics of study participants (eg demographic, clinical, social) and information on exposures and potential confounders	7-8
		(b) Indicate number of participants with missing data for each variable of interest	7
Outcome data	15*	Report numbers of outcome events or summary measures	8

For peer review only - http://bmjopen.bmj.com/site/about/guidelines.xhtml

Main results	16	(*a*) Give unadjusted estimates and, if applicable, confounder-adjusted estimates and their precision (eg, 95% confidence interval). Make clear which confounders were adjusted for and why they were included	8-9
		(*b*) Report category boundaries when continuous variables were categorized	
		(*c*) If relevant, consider translating estimates of relative risk into absolute risk for a meaningful time period	NA
Other analyses	17	Report other analyses done—eg analyses of subgroups and interactions, and sensitivity analyses	10-12
**Discussion**			
Key results	18	Summarise key results with reference to study objectives	10
Limitations	19	Discuss limitations of the study, taking into account sources of potential bias or imprecision. Discuss both direction and magnitude of any potential bias	15-16
Interpretation	20	Give a cautious overall interpretation of results considering objectives, limitations, multiplicity of analyses, results from similar studies, and other relevant evidence	12-15
Generalisability	21	Discuss the generalisability (external validity) of the study results	15-16
**Other information**			
Funding	22	Give the source of funding and the role of the funders for the present study and, if applicable, for the original study on which the present article is based	16

*Give information separately for exposed and unexposed groups.

Note: An Explanation and Elaboration article discusses each checklist item and gives methodological background and published examples of transparent reporting. The STROBE checklist is best used in conjunction with this article (freely available on the Web sites of PLoS Medicine at http://www.plosmedicine.org/, Annals of Internal Medicine at http://www.annals.org/, and Epidemiology at http://www.epidem.com/). Information on the STROBE Initiative is available at www.strobe-statement.org.

For peer review only

BMJ Open

Women's Preference to Apply Shared Decision Making in Breast Cancer Screening: A Discrete Choice Experiment

Journal:	*BMJ Open*
Manuscript ID	bmjopen-2022-064488.R2
Article Type:	Original research
Date Submitted by the Author:	12-Oct-2022
Complete List of Authors:	Hernández Leal, María José; Universitat Rovira i Virgili, Economic ; Research Group on Statistics, Economic Evaluation and Health (GRAEES).<br>Pérez-Lacasta, María José; Universitat Rovira i Virgili, Economic ; Research Group on Statistics, Economic Evaluation and Health (GRAEES).<br>Cardona, Angels; Area Q: Evaluation and Research in the Field of Social Sciences and Health<br>Codern-Bové, Núria; Autonomous University of Barcelona, School of Nursing and Occupational Therapy (EUIT)<br>Vidal, Carmen; Catalan Institute of Oncology, Cancer Prevention and Control Program<br>Rue, Montserrat; University of Lleida, Basic Medical Sciences; Research Group on Statistics, Economic Evaluation and Health (GRAEES). ,<br>Forné, Carles; University of Lleida, Department of Basic Medical Sciences; Heorfy Consulting, HEOR freelance consultant<br>Carles-Lavila, Misericòrdia; Universitat Rovira i Virgili, Economics; Research Group on Statistics, Economic Evaluation and Health (GRAEES).
**Primary Subject Heading**:	Health economics
Secondary Subject Heading:	Communication, Diagnostics, Health economics, Patient-centred medicine, Research methods
Keywords:	HEALTH ECONOMICS, Breast tumours < ONCOLOGY, PREVENTIVE MEDICINE, PRIMARY CARE

SCHOLARONE™
Manuscripts

I, the Submitting Author has the right to grant and does grant on behalf of all authors of the Work (as defined in the below author licence), an exclusive licence and/or a non-exclusive licence for contributions from authors who are: i) UK Crown employees; ii) where BMJ has agreed a CC-BY licence shall apply, and/or iii) in accordance with the terms applicable for US Federal Government officers or employees acting as part of their official duties; on a worldwide, perpetual, irrevocable, royalty-free basis to BMJ Publishing Group Ltd ("BMJ") its licensees and where the relevant Journal is co-owned by BMJ to the co-owners of the Journal, to publish the Work in this journal and any other BMJ products and to exploit all rights, as set out in our licence.

The Submitting Author accepts and understands that any supply made under these terms is made by BMJ to the Submitting Author unless you are acting as an employee on behalf of your employer or a postgraduate student of an affiliated institution which is paying any applicable article publishing charge ("APC") for Open Access articles. Where the Submitting Author wishes to make the Work available on an Open Access basis (and intends to pay the relevant APC), the terms of reuse of such Open Access shall be governed by a Creative Commons licence – details of these licences and which Creative Commons licence will apply to this Work are set out in our licence referred to above.

Other than as permitted in any relevant BMJ Author's Self Archiving Policies, I confirm this Work has not been accepted for publication elsewhere, is not being considered for publication elsewhere and does not duplicate material already published. I confirm all authors consent to publication of this Work and authorise the granting of this licence.

For peer review only

Original Research
BMJ Open

Women's Preference to Apply Shared Decision Making in Breast Cancer Screening: A Discrete Choice Experiment

Running head: A DCE for SDM in breast cancer screening

María José Hernández-Leal[a,b,c] María José Pérez-Lacasta [a,c] Angels Cardona[d] Núria Codern-Bové[e] Carmen Vidal-Lancis[f] Montserrat Rué[c,g] Carles Forné[h,i], Misericòrdia Carles-Lavila [a,b,c] on behalf of the Pro-Share Group

a. Department of Economics, University Rovira i Virgili, Reus, Spain.
b. Research Centre on Economics and Sustainability (ECO-SOS), Reus, Spain.
c. Research Group on Statistics, Economic Evaluation and Health (GRAEES), Reus, Spain.
d. Area Q: Evaluation and Research in the Field of Social Sciences and Health, Barcelona, Spain.
e. School of Nursing and Occupational Therapy (EUIT), Autonomous University of Barcelona, Terrassa, Spain.
f. Cancer Prevention and Control Program, Catalan Institute of Oncology-IDIBELL, L'Hospitalet de Llobregat, Spain
g. Department of Basic Medical Sciences, University of Lleida-IRB, Lleida, Spain.
h. Department of Basic Medical Sciences, University of Lleida, Spain.
i. HEOR freelance consultant, Heorfy Consulting, Reus, Spain.

Pro-Share Group: Misericòrdia Carles-Lavila, Núria Codern-Bové, María José Hernández-Leal, María José Pérez, Roger Pla, Vanesa Ramos, Carmen Vidal

Corresponding author: Misericòrdia Carles-Lavila, Department of Economics, University Rovira i Virgili, at Av. de la Universitat 1, 43204, Reus, Spain.
Email: misericordia.carles@urv.cat

For peer review only

ABSTRACT

Objective: To analyse women's stated preferences for establishing the relative importance of each attribute of Shared Decision Making (SDM) and their willingness to pay (WTP) for more participatory care in breast cancer screening programmes (BCSP).
Design: A Discrete Choice Experiment was designed with 12 questions (choice tasks). It included three attributes: 'How the information is obtained', regarding benefits and harms; whether there is a 'Dialogue for scheduled mammography' between the healthcare professional and the woman; and, 'Who makes the decision regarding participation in BCSP. Data were obtained using a survey that included 12 choice tasks, one question on WTP, and seven socio-economic-related questions. The analysis was performed using conditional mixed-effect logit regression and stratification according to WTP.
Setting: Data collection related to BCSP was conducted between June and November 2021 in Catalonia, Spain.
Participants: Sixty-five women aged between 50 and 60.
Main outcome measures: Women's perceived utility of each attribute, trade-off on these attributes, and WTP for SDM in BCSP.
Result: The only significant attribute was 'Who makes the decision'. The decision made by oneself alone (coefficient=2.879; 95% CI=2.297, 3.461) and the decision made together with a healthcare professional (2.375; 95%CI=1.573, 3.177) were the options preferred by women. The former contributes 21% more utility than the latter. Moreover, 52.3% of the women stated a WTP 10 euros or more for SDM. Women's preferences regarding attributes did not influence their WTP.
Conclusions: The participant women refused a current paternalistic model and preferred either SDM or Informed Decision making in BCSP.

Keywords: Shared Decision-making; Informed Decision-making; Breast Cancer screening; Discrete Choice Experiments; Willingness to pay.

Strengths and limitations of this study

- DCEs have limitations on the sample size and SDM, as a little-known model, which makes it difficult for participants to understand the hypothetical situations described in the choice task.
- A DCE is an indirect method to obtain stated preferences and information that otherwise would be impossible to reveal when actual choice behaviour is, in some way, restricted.
-The choice experiments were tested and refined through a two-stage piloting process.
-The responses obtained from participants' WTP may be sensitive when performed within a public health system context.
-This is the first study concerning women's preferences related to SDM in BCSPs.

For peer review only

1. BACKGROUND

Breast cancer (BC) screening programmes have been widely adopted in developed countries because of their early detection of malignant lesions, thereby reducing mortality and improving the survival rate (1,2). In 2020 34,000 cases were identified and 6,600 deaths recorded in Spain due to BC (3). The screening programme in the Spanish region of Catalonia is scheduled by the Public Health System. This screening programme entails sending a postal letter to women aged between 50 and 69 every 2 years to undergo a mammography free in a health centre (4). Several research and communication campaigns have focused on women's increasing adherence to screening to raise awareness of its importance, which in 2017 led the Spanish coverage reach 81.5% in the target population (although it would drop to 73.7% 3 years later, during the pandemic) (5). The most cited reason for attending it is the invitation letter issued by the organisations affiliated with the screening programme (6).

However, research also recognises the adverse aspects of BC screening, which are usually not explained to women in a balanced way (7). These include false positives, false negatives, overdiagnosis, and consequently, overtreatment (8). Overdiagnosis refers to screen-detected malignancy that would not have developed into clinical or symptomatic disease and would have never caused health problems throughout the woman's life (9). Currently, it is impossible to identify which lesions may progress. Therefore, all lesions are generally treated.

In cases of uncertainty, the literature recommends Shared Decision Making (SDM) (10). SDM is characterised as a participatory care model in which both the professional and the patient are on a par with each other in terms of power to deliberate the best decision. This contrasts with the paternalistic health model, in which the health professional or the system makes such decisions (11). The latter grew stronger in some health programmes despite declarations and intentions to change it to a more participatory one. SDM allows women, according to their own beliefs and values, to decide together with a health professional whether to undergo a mammography (12) while considering scientific evidence and the options available to them. SDM has shown multiple benefits (13); despite that, it is necessary to examine its costs, quality, and efficiency (13) to determine its applicability in different contexts. Some studies indicate that the application of SDM can mean a reduction in health expenses (14), whereas other studies suggest that the degree of such savings is unclear (15,16). Thus, from an economic-health perspective, an approach to cost-benefit analysis can be generated through Discrete Choice Experiments (DCEs) (17).

Using DCE allows to determine the utility of more participatory healthcare in BC screening, enabling women to declare their preferences as well as monetising the intangible values that improve the patient`s satisfaction (18,19).

There is a dearth of studies focusing on awareness of patients' preferences in SDM (20), and there are none on women's preferences regarding breast cancer screening programmes (BCSP) and their willingness to pay (WTP) for them. Therefore, this study is aimed to analyse women's declared preferences in Catalonia regarding the attributes of more participatory care (SDM) in contrast to the usual care, which reflects characteristics of a paternalistic model for BC screening; all this by establishing the relative importance of each

For peer review only - http://bmjopen.bmj.com/site/about/guidelines.xhtml

one through a DCE. In addition, the study participants were asked about their WTP for this type of healthcare.

2. METHODS

Our DCE design includes eight hypothetical profiles, referred to as choice sets; each one with three characteristics—attributes—that can present different values or termed levels. For the pairing of these attributes and levels a factorial design is considered, assuming that they are independent from each other. Finally, each *choice set* contrasts with another forming a *choice task* (21) that is presented to women to let them choose as per their preference.

2.1. Definitions of attributes and levels

The Three Talk model describes and simplifies the implementation of SDM in three steps (22,23). The attributes and levels of this experiment were defined considering the model and adapting it to the characteristics of the current BCSP. Table 1 describes the levels of a participatory healthcare model with characteristics of SDM (a health professional informs the patient of both risks and benefits and, there is a dialogue between the woman and the healthcare professional (who jointly make the decision) as well as the current model of care, which has characteristics of a paternalistic model (the risks and benefits are provided via a leaflet, the healthcare system schedules the mammography according to age criteria, and the healthcare professional makes the decision). Moreover, included in the third attribute ('Decision talk') there is an additional level wherein the woman makes a decision for herself, that is, informed decision-making (IDM). Here, women receive the same information as in the first two attributes, but it is only their responsibility to decide whether to participate in the screening or not.

Table 1. Attributes and levels of the options for shared decision making in breast cancer screening

Attribute	Definition	Model	Description	Levels
**1. Information talk**	How the information is obtained	Paternalist	Women are informed via a leaflet on the risks and benefits of BC screening	Leaflet
		SDM	Women are informed by a health professional of the risks and benefits of BC screening	Healthcare professional
**2. Dialogue talk**	Dialogue for mammography scheduled	Paternalist	The health system schedules mammography screening appointments in relation to age criteria	The health system schedules the mammography
		SDM	A discussion of therapeutic options and women's values determines the appointment for BC screening	Women share and discuss their beliefs and values regarding the

For peer review only - http://bmjopen.bmj.com/site/about/guidelines.xhtml

				mammography with a healthcare professional
**3. Decision talk**	Who makes the decision	Paternalist	The decision is made by the healthcare professional.	The healthcare professional makes the decision
		SDM	The decision is made through a deliberative process between the health professional and the woman	Shared decision making by the healthcare professional and the woman
		IDM	The decision is made exclusively by the woman without the health professional's support	The woman makes the decision

SDM: Shared Decision-making; IDM: Informed Decision-making: BC: breast cancer

The current functioning of the BCSP in the Spanish region of Catalonia was used to determine the levels for each attribute: the health system schedules a mammography appointment every 2 years for women aged between 50 and 69 via an invitation letter; an informational leaflet is attached to the invitation letter and, women decide whether to attend it. Conversely, in relation to SDM, women inform themselves through a health professional, meeting him/her to discuss their beliefs regarding screening, and decide whether to undergo a mammography.

As a result, two levels were established for both the 'Information talk' and the 'Dialogue talk' attributes representing the functioning of SDM; the typical model and three levels were considered for the last attribute, 'Decision talk' (Table 1).

2.2. Willingness-to-pay

There is no previous literature on WTP regarding SDM in BCSP. Therefore, the options were determined according to the average value (40€) of a consultation with a primary care doctor in Spain (24), and a variation (30€) was considered for the fraction of time devoted to SDM. Moreover, we considered other research papers that have analysed the way to introduce SDM, which indicate that the ideal context is the primary care context, with either a physician or a nurse to apply the SDM model (25).

2.3. Experimental design

In this phase, attributes and levels are combined to create different choice sets to be evaluated by women. The number of choice sets depends on the number of attributes and their levels. Following Reed Johnson et al.'s suggestions (26), four-choice sets were removed because they were neither plausible nor consistent combinations with the theory, leaving then a total of eight choice sets. Those would be eliminated if, without prior discussion, the professional and the woman jointly decided whether to participate, no matter if they had received the relevant information via a leaflet or by a healthcare professional.

The eight choice sets presented in pairs resulted in a maximum of 28 tasks (the action of choosing between two alternatives). Many of these could be eliminated, such as in the 16 cases in which one alternative was dominant or dominated by the other. Since SDM is

For peer review only

considered a better option to resolve uncertainty in this context (27), it was considered a dominant choice set. The remaining 12 choice tasks were incorporated into the DCE survey, as this number is below the maximum number of tasks that can be included without causing fatigue on the participants (28,29).

2.4. Patient and public involvement: Piloting and refinement of the questionnaire

In March 2021, a snowball sampling was carried out. Eleven women completed the questionnaire, which helped clarify its difficulty level and ensure that the questions could be understood. This pre-pilot questionnaire allowed us to identify the difficulties in understanding the elements to be evaluated. The choice tasks were reconfigured from the conventional (structured) arrangement to a descriptive one that narrated a hypothetical scenario with the three attributes in question. To better clarify structured vs descriptive surveys, the two versions are shown in Figure 1. Subsequently, a randomised pilot study was conducted on 10 women to evaluate the recruitment process and the survey was performed in a mixed procedure: online (self-applied) and on the telephone (guided) (29).

Figure 1. Examples of a choice task for conventional (first version) and narrative (final version)

The survey was conducted between June and November of the same year in both Spanish and Catalan languages. Women were invited to participate, voluntarily and unpaid, through a phone call in which they were informed of the objectives of the research project and if they agreed to participate, then they were asked to complete a survey on the telephone or online. Those who did not complete the survey within one week were reminded to do so (Figure 2). The 12-choice task corresponding to the DCE included seven questions on sociodemographic aspects (age, nationality, marital status, education, employment status, family history of BC, and participation in screening) and also a single, multiple-choice question on WTP for BC screening care with SDM characteristics (Annex 1).

Figure 2. Net follow-up of survey responses

2.5. Participants

The study was conducted in Catalonia, Spain, with a random sample of women aged between 50–60 who participated in three BCSP: at Hospital del Mar in Barcelona, in the Cancer Prevention and Control Program of the Catalan Institute of Oncology, and in the Health Region of Lleida. This population had already been invited, at least once, through the national programme to undergo a mammography. Therefore, it was assumed that the topic was more sensitive to their interests. The women who had been diagnosed with BC were excluded from the study.

The minimum required sample size was estimated to be 63 participants, whose calculation was based on the empirical rule recommended by Johnson and Orme (30).

For peer review only

$$N>(500c)/(t*a).$$

N=number of respondents, t=number of choice tasks, a=number of alternatives, and c= maximum number of levels.

2.6 Data analysis

A mixed-effect-conditional logit model was used to estimate women's preferences for different levels of SDM attributes in BC screening. This was based on Daniel McFadden's theory of Discrete Choice, which seeks to describe the behaviour of decision-makers when facing a decision problem, assuming that the declared preferences of those elections are based on the maximum possible-utility achievement (31). The mixed-effect logistic regression model enables the heterogeneity of preferences in the sample by treating the coefficients as random. It also allows multiple observations from each respondent, which is appropriate for our study, it presenting each woman 12 choice tasks. All models included main effects without interaction terms. A model for each subgroup was fitted to allow comparisons according to the WTP.

All attribute variables were coded as dummy variables, with reference levels identified with their results as shown in tables. Furthermore, they were specified as having a random component, assuming a normal distribution for all model coefficients. These coefficients indicate a change of preference from the reference level for each attribute (32).

When interpreting the model results, the statistical significance of the coefficients indicates whether attribute levels influence the choice set; whereas the coefficient size indicates the relative importance from one attribute level to another one. We did not include an alternative-specific constant variable because our choice sets unlabelled. Therefore, they had no utility beyond the attributes assigned to them in the experiment.

For all analyses, statistical significance was set at 0.05. All statistical analyses were performed using the R statistical software.

3. RESULTS

Of the 292 women invited to participate, 93 accepted; 27 did not reply, and 66 submitted the survey (reflecting a 70.97% response rate); and one being excluded because of a history of BC. Therefore, a total sample of 65 women was obtained; the responses from two were collected on the phone, and those from the remaining 63 were collected via online forms (Figure 3). The participants' characteristics are presented in Table 2.

Figure 3. Flowchart of participants

Table 2. Characteristics of participants

**Characteristic**

For peer review only - http://bmjopen.bmj.com/site/about/guidelines.xhtml

Age	56.9	(1.34)
Birthplace		
Spain	57	(87.7%)
Other country	8	(12.3%)
Relationship status		
Single	17	(26.2%)
With a partner	48	(73.8%)
Education		
Primary	18	(27.7%)
Secondary	26	(40.0%)
University	21	(32.3%)
Main occupation		
Unemployed	6	(9.23%)
Paid work	48	(73.8%)
Homemaker	11	(16.9%)
Family history of breast cancer		
No	50	(76.9%)
Yes	14	(21.5%)
Unknown	1	(1.54%)
Do you have a mammogram regularly?		
No	11	(16.9%)
Yes	54	(83.1%)

Categorical variables are summarised using frequency and percentage. Age is summarised using mean and standard deviation.

3.1. Discrete Choice Experiment results and trade–off

According to the mixed-effect-conditional logit model, the weighting for each attribute was estimated using the responses to the 12 tasks. There were no missing values among the responses.

The results show that the attribute ‘Decision talk’ was the only important one for women when considering SDM in BC screening. Women preferred to make decisions alone (coefficient=2.879; 95%CI=2.297, 3.461) or to engage in SDM with healthcare professionals (coefficient=2.375; 95%CI=1.573, 3.177), in comparison to having a healthcare professional make the decision for them (Table 3). The marginal rate of substitution (MRS) between the two coefficients for the person who made the decision was 1.21. This meant that women perceive 21% more utility when making decisions than making them with a healthcare professional.

The attributes ‘Information talk’ and ‘Dialogue talk’ showed no significant results. Therefore, women do not mind to either receiving information on the benefits and risks of mammography through a leaflet or being informed by a healthcare professional

For peer review only - http://bmjopen.bmj.com/site/about/guidelines.xhtml

(coefficient=-0.168; 95% CI=-0.665, 0.329). Neither did they show any clear preference for an appointment with a healthcare professional to discuss their beliefs in scheduling their next mammography over a standard screening schedule (coefficient=0.145; 95% CI=-0.373, 0.663).

Table 3: Results from the mixed-effects conditional logit model

Attribute	Levels	Coefficient/SD	SE	p-value
**1. Information talk**	Leaflet	Reference category	-	-
	Healthcare professional, mean	-0.168	0.2536	0.509
	Healthcare professional, SD	1.473	0.3206	<0.001
**2. Dialogue talk**	No, health system schedules mammography	Reference category	-	-
	Yes, women share and discuss their beliefs and values about mammography with a healthcare professional, mean	0.145	0.2645	0.583
	Yes, women share and discuss their beliefs and values about mammography with a healthcare professional, SD	1.371	0.3212	<0.001
**3. Decision talk**	The healthcare professional makes the decision	Reference category	-	-
	Shared decision making by the	2.375	0.4093	<0.001

For peer review only - http://bmjopen.bmj.com/site/about/guidelines.xhtml

healthcare professional and the woman, mean			
Shared decision making by the healthcare professional and the woman, SD	1.415	0.5208	0.007
The woman makes the decision, mean	2.879	0.2967	<0.001
The woman makes the decision, SD	1.988	0.2626	<0.001

Table 3: SD, standard deviation (of the random effects); SE, standard error.

For peer review only

3.2. Willingness to pay for Shared Decision making

More than half of the participants (52.3%) were willing to pay for the SDM process in BC screening; most (38.5%) would be willing to pay between 10€ and 30€, and the remaining ones (13.8%) would be willing to pay 40€ (Table 4).

Table 4: Women's willingness to pay for Shared Decision Making in breast cancer screening

**Co-payment (euros)**	**n**	**%**
0	31	(47.7%)
10	6	(9.23%)
20	6	(9.23%)
30	13	(20.0%)
40	2	(3.08%)
60 or more	7	(10.8%)

Two regression models were fitted for the sample of women according to their WTP: those who showed WTP and those who did not (Table 5). The results for both subgroups were similar to those obtained for the entire sample. Thus, the attribute 'who makes the decision' was the only important one for women regardless their WTP.

In addition, the results for the subgroups show the same trends in women's preferences regarding both 'Information talk' and 'Dialogue talk', as in the regression model for the entire sample.

For peer review only - http://bmjopen.bmj.com/site/about/guidelines.xhtml

Table 5: Results from the mixed-effect-conditional logit model stratified by willingness to pay.

		Not willing to pay			Willing to pay		
**Attribute**	**Levels**	**Coef./SD**	**SE**	**p-value**	**Coef./SD**	**SE**	**p-value**
**1. Information talk**	Leaflet	Ref.	-	-	Ref.	-	-
	Healthcare professional, mean	-0.323	0.3706	0.383	-0.074	0.3768	0.845
	Healthcare professional, SD	1.537	0.4233	<0.001	1.434	0.4684	0.002
**2. Dialogue talk**	No, health system schedules mammography	Ref.	-	-	Ref.	-	-
	Yes, women share and discuss their beliefs and values about mammography with a healthcare professional, mean	0.187	0.3944	0.636	0.136	0.3825	0.721
	Yes, women share and discuss their beliefs and values about mammography with a healthcare professional, SD	1.599	0.4653	<0.001	1.479	0.4756	0.002
**3. Decision talk**	The healthcare professional makes the decision	Ref.	-	-	Ref.	-	-
	Shared decision making by the	2.220	0.5852	<0.001	2.826	0.6392	<0.001

For peer review only - http://bmjopen.bmj.com/site/about/guidelines.xhtml

healthcare professional and the woman, mean						
Shared decision making by the healthcare professional and the woman, SD	1.449	0.5600	0.010	1.649	0.8826	0.062
The woman makes the decision, mean	2.784	0.4220	<0.001	3.006	0.4328	<0.001
The woman makes the decision, SD	1.803	0.3734	<0.001	2.548	0.4410	<0.001

Coef., coefficient; SD, standard deviation (of the random effects); SE, standard error; Ref., reference category.

4. DISCUSSION AND CONCLUSION

4.1. Main findings

This study analysed women's preferences for the attributes of SDM—Information talk, Dialogue talk, and Decision talk—in a BCSP through a DCE. The main result is that women prefer to make decisions themselves or together with the healthcare professional, while they do not like the healthcare professional making the decision for them. Women perceive more utility in deciding for themselves than in deciding jointly with the healthcare professional. More than half of the women were willing to pay for screening with SDM characteristics. There was no difference in the responses obtained between women with a positive WTP and those who were not willing to pay. Regarding methodology, this study has a strong response rate (>70%) due to the close monitoring of participants' responses (33).

This study is important for its contribution to the scarce existing literature on the economic quantification of SDM, research which has always been carried out in the form of qualitative studies. Moreover, most studies have focused on its applicability to public health systems; few have examined the topic from an economic perspective, and even fewer have focused on WTP using a DCE methodology.

4.2. Attribute implications

For the first attribute, ***Information talk***, the women in our study did not show a clear preference to be given an informational leaflet explaining, in a simple and balanced way, the benefits and risks of screening instead of having this information explained by a health professional. The lack of difference between the two alternatives may be related to the fact that women do not perceive any difference in the information received. This is supported by studies such as the one conducted by Longo et al. (20), who performed a DCE to determine

For peer review only - http://bmjopen.bmj.com/site/about/guidelines.xhtml

the SDM preferences of patients` in the context of chronic diseases. In their study they reported that the second most important attribute for patients was that information could be easily understood. Therefore, using a patient decision aid (PtDA) may help women to better understand the information.

PtDAs deliver balanced information on the risks and benefits of BC screening, which increases informed decision-making (IDM) (34). In a recent study in Spain (35), 23% of women who received information in this way made informed decisions about their participation in screening, compared to 0.5% in the control group, who had only received information attached to the invitation letter provided by the BCSP. PtDAs have the advantage of being repeatedly consulted by women to review information, as opposed to a clinical appointment. However, the two strategies could act in a complementary fashion, and PtDAs could be utilised to prepare for the latter appointments to resolve doubts and review the information they contain (36).

Some institutions, such as the Public BCSP in Catalonia, have already incorporated an invitation letter for screening with a leaflet including information on the explicit need to make a decision, such as overdiagnosis, the balance of benefits and risks, and scientific uncertainties (37). Documentation is scarce in Spain, and until recently, various programmes have not provided balanced information on the benefits and adverse effects of early detection testing (38).

Improving the information provided in the programme is proposed as a strategy of PtDAs, which can be used with individuals of different literacy levels; in the local context of this study, they would ideally be used with women of a higher educational level because they are really interested in a SDM (20), and are able to understand complex information such as prevalence and absolute risks (39,40) without needing an explanation by a healthcare professional.

Regarding the second attribute, ***Dialogue talk***, the women in our study were indifferent in regard to the choice between attending a face-to-face appointment with a healthcare professional to discuss their beliefs and concerns about participation in the screening and the standard approach, in which the healthcare system schedules the screening. These results may be due to the wide acceptance of screening among women not only because of the well-known benefits but also the minimisation of risks; information which has been disseminated by persuasive preventive public health campaigns of different governments. This has led women to unquestionably participate in screening and more openly discuss on the benefits and risks as well as and on their beliefs and fears (41).

However, in a study by Longo et al. (2006) (20), the most relevant attribute for patients was their need to feel heard by their physicians, particularly because patients reported the feeling of not being listened to (42). Thus, communication remains an element that should be considered by healthcare professionals when they interact with their patients. Showing a receptive attitude, acceptance, empathy, and lack of judgment are the first steps in generating a trustful relationship and thus advancing to a more participatory health model (43). Without this, it is impossible to have awareness of the beliefs of patients' and, therefore, encourage

For peer review only - http://bmjopen.bmj.com/site/about/guidelines.xhtml

them to participate in the process of SDM. This requires training in competences and communication skills (42).

Conversely, there is a high probability that women already have beliefs or fears regarding BC and want to discuss them with a health professional. In particular, the fact that having BC and having survived it is not unusual (6), it means that women usually know other women already diagnosed with or treated for BC. In our study, one out of five women had a first-degree relative with a history of BC. This would increase, if the scope were extended to other affective bonds such as friends and acquaintances. This is interesting when considering personalisation of the screening, in which women must be assisted by a healthcare professional to determine whether their risk is high, medium, or low, and thus recommendations must be made for the frequency of screening through an SDM process (44).

Regarding the third attribute, ***Decision talk***, women reject paternalistic health models, in which the healthcare professional or health system exclusively makes health decisions. This result is consistent with the current trend of empowering patients to be more autonomous in clinical decisions (34,37). However, this degree of autonomy may be unrealistic because of the lack of knowledge regarding adverse effects (45). For example, a recent study indicated that Spanish women have low awareness of overdiagnosis (46,47). Only 8.1% knew the meaning of overdiagnosis, even though, the percentage increased to 54.2% in women who had received PtDAs (47).

In Longo et al.'s study (20), patients preferred to make decisions for themselves or with a healthcare professional and rejected the approach of the decision being made exclusively by a professional, which accords with our results. However, in Longo's study, this attribute was one of the least important.

The characteristics of the clinical process differ either when deciding on treatment or when working in an early detection context, in which the target population is healthy. An option well-evaluated by women in previous studies (48) is recommending the development of a targeted SDM to specific groups of women, such as those who need more information on the benefits and risks, those who have their first experience with screening, those who have lower educational levels, and those who have a high risk of being diagnosed with BC. All of them require face-to-face appointments with healthcare professionals (36,49). However, the implementation of Informed Decision making (IDM) for women with a high educational level and a low risk of being diagnosed with BC would be sufficient, and an appointment with a healthcare professional would be unnecessary (50). This second group can be given PtDAs with simple and clear information on screening for them to make the decision autonomously (34,51,52), which implies a lack of follow-up by the healthcare professional. A study is currently being conducted at the Mayo Clinic to determine whether women's discussion groups for decision making could provide a new line to support and prepare women for deciding on BC screening (53). This is based on the strategy mentioned above: the PtDAs could be delivered before the clinical meeting, and subsequently there would be a conversation with other women about the information provided there. As such, the appointment between the healthcare professional and the woman would only aim at clearing up doubts or concerns originated from the PtDAs (36).

For peer review only - http://bmjopen.bmj.com/site/about/guidelines.xhtml

In conclusion, the results of the DCE allow us to analyse SDM barriers and the difficulties in its applicability (40,54,55) and because of this, the main approaches have been developed within contexts in which decision making is particularly difficult (55). Besides, there are still few examples of studies like ours to be applied in primary care, where patients make less difficult decisions, yet, not less important for their impact on people's quality of life (51).

4.3. Willingness to pay

The subgroups, separating those who were or were not willing to pay, showed a preference for making decisions jointly or alone. No studies were found regarding WTP for SDM in the context of BC screening. Only one article for patients with BC diagnosis was found, but the results focused on WTP according to the type of treatment and not on SDM characteristics (56). Another study, focused on prostate cancer, and reported that participants with high WTP preferred an active rather than a passive participation model (57).

Regarding the monetary value of SDM, this study's findings can be compared to the results of Wilson et al.'s (57) study on patients with prostate cancer in the United States. It was determined that men had a WTP between $25 and $50 for the implementation of SDM (57). WTP was associated with marital status (single people valued SDM more highly), the stage of SDM (those who were initiating the process of decision making were more likely to be willing to pay compared to those who were close to deciding), and the participation model (more active patients are more likely to be willing to pay compared to passive ones) (57).

In addition, Brito et al. (2020) (58) evaluated patient-reported experiences among adult population in outpatient care through a DCE, in which SDM characteristics were included. In this case, respondents were willing to pay, on average, €16 for a doctor who provided easy-to-understand explanations in comparison to one who did not provide understandable ones. They were also willing to pay, on average, €20 for physicians to whom they could ask questions or raise concerns as opposed to physicians who focused on providing information. Finally, they were willing to pay, on average, €22 to physicians who involved the patient in making decisions, in contrast to those who made decisions in an only manner (58).

Finally, it should be considered that in Spain, screening is fully covered by the National Health System (NHS) for women aged between 50–69. Although it is free, and therefore, the ability to pay does not effectively coincide with the real payment, WTP shows how much society is willing to pay for a healthcare innovation; specifically, in our case, its willingness to pay for a relationship between professional and patient in the clinical appointments (59). and, in the case of innovation being implemented in the NHS, it may imply a burden on the health system and co-payment or direct payment from women (14). In the last case, the out-of-pocket cost for a screening is one of the determining factors for adherence to SDM in BCSP (60).

Future research could include randomised clinical trials, in which women would experience healthcare with all the attributes that SDM elicits; their preferences, perceived satisfaction, and health benefits could be analysed in comparison to regular attendance; and the costs that

For peer review only - http://bmjopen.bmj.com/site/about/guidelines.xhtml

the implementation of SDM would entail in the screening of BC for the NHS could be identified.

4.4. Limitations

The main limitation of the study, which forced us to change the design of the questions, was the difficulty women experienced when comparing hypothetical profiles. We detected difficulties in understanding the differences between the profiles proposed in each choice task. In addition, their ability to detect differences among the 12 questions in the DCE survey was limited. This may also be due to women's lack of familiarity with participatory health characteristics because of the current BCSP model (exposure bias). Moreover, the decisions regarding screening seem to reflect a social consensus on the need for screening and thus its utility is not questioned. To eliminate exposure bias, future randomised studies should be conducted among women who can experience both models of care (paternalistic and SDM), and then, their preferences and perceived satisfaction can be compared to their experience of regular attendance to identify the costs that the implementation of SDM would entail in the BCSP. Considering that 'who makes the decision' is the most important factor for women, strategies could be proposed to ensure that through discussion groups, women can make an informed decision, as contexts of prevention, in contrast to those of treatment, which may be more accepted by women (53). These new procedures are currently being introduced in studies conducted at the Mayo Clinic, among others which aimed at facilitating more participatory and more informed decision making (53).

Finally, it is difficult to precisely determine the ability to pay because in Spain, BC screening is within the purview of the NHS. Therefore, services are free at the time they are provided; and the population assumes that this is a consolidated health benefit and consequently something not to be paid for, in particular because screening for other types of cancer (e.g., colorectal) is currently being introduced and heavily promoted by the NHS.

4.5. Conclusion

This is the first study examining women's preferences regarding SDM in relation to BC screening. Women were shown to reject paternalistic health models in the context of BCSP and to prefer models with IDM or SDM. More than half of the participants were willing to pay for active involvement in their health decisions.

4.6. Practical Implications

The change from a paternalistic model to a participatory model of person-centred medicine would require restructuring the BCSP so that women can make informed decisions for themselves or engage in SDM together with a healthcare professional.

5. CONTRIBUTION STATEMENT

María José Hernández-Leal, María José Pérez-Lacasta, and **Misericòrdia Carles-Lavila**: Conceptualisation, Methodology, Formal analysis, Investigation, Project administration, Supervision, Visualisation, Writing-original draft. **Angels Cardona** and **Núria Codern-Bové**: Data curation, Formal analysis, Writing-review and editing. **Carmen**

For peer review only - http://bmjopen.bmj.com/site/about/guidelines.xhtml

Vidal-Lancis: Resources, Writing-review and editing. **Montserrat Rué**: Resources, Formal analysis, Writing-review and editing. **Carles Forné**: Software, Formal analysis, Writing-review and editing.

6. COMPETING INTERESTS

The authors declare that they have no conflict of interest.

7. FUNDING INFORMATION

Financial support for this study was entirely provided by a grant from Instituto de Salud Carlos III through the project PI18/00773 (co-funded by European Regional Development Fund) and the European Union's Horizon 2020 research and innovation programme under Marie Skłodowska-Curie grant agreement No. 713679 from the Universitat Rovira i Virgili (URV). The funding agreement ensures the authors' independence in designing the study, interpreting data, and writing and publishing the report.

8. DATA SHARING STATEMENT

All data relevant to the study are included in the manuscript and tables or uploaded as supplementary information.

9. ETHICAL APPROVAL STATEMENT

This research was approved by the Medicinal Product Research Ethics Committee (CEIm) of the Institut d'Investigació Sanitària Pere Virgili (Pere Virgili Health Research Institute) Ref. CEIM: 175/2019. Participants in the informed consent agreed to participate in the study and it was established that the responses were confidential and would be used only for this research. Informed consent in the case of telephone participants was verbal. While those who did it online had to accept to be part of the study through the virtual platform previous to their accessing the survey.

10. REFERENCES

1. Kerlikowske K, Grady D, Rubin SM, Sandrock C, Ernster VL. Efficacy of screening mammography. A meta-analysis. JAMA. 1995;273(2):149-154. PMID: 7799496
2. Walter LC, Lewis CL, Barton MB. Screening for colorectal, breast, and cervical cancer in the elderly: a review of the evidence. Am J Med. 2005;118(10):1078-1086. doi: 10.1016/j.amjmed.2005.01.063.
3. Sung H, Ferlay J, Siegel RL, Laversanne M, Soerjomataram I, Jemal A, et al. Global cancer statistics 2020: GLOBOCAN estimates of incidence and mortality worldwide for 36 cancers in 185 countries. CA Cancer J Clin. 2021;71(3):209-249. doi:https://doi.org/10.3322/caac.21660.
4. Institut Català d'Oncologia, de Càncer de Mama DP. [Early breast cancer detection]. 2020. Available from: http://gencat.cat/ca/el-cancer/programes_de_deteccio_precoc/programa_de_deteccio_precoc_de_cancer_de_mama/index.html.

For peer review only - http://bmjopen.bmj.com/site/about/guidelines.xhtml

5. Ministerio de Sanidad, Servicios Sociales e Igualdad, Instituto Nacional de Estadística. Encuesta europea de Salud en España EESE 2020 [European survey of health in Spain EESE 2020]. Madrid: ministerio de sanidad. 2020. Available from: https://www.sanidad.gob.es/estadEstudios/estadisticas/EncuestaEuropea/EncuestaEuropea2020/EESE2020_inf_evol_princip_result.pdf.
6. Ministerio de Sanidad, Servicios Sociales e Igualdad, Instituto Nacional de Estadística. Encuesta europea de salud en España EESE 2017 [European survey of health in Spain EESE 2017]. 2010. Disponible enhttps: Available from: http://www.mscbs.gob.es/estadEstudios/estadisticas/encuestaNacional/encuestaNac2017/DETECCION_PRECOZ_CANCER.pdf.
7. Jørgensen KJ, Klahn A, Gøtzsche PC. Are benefits and harms in mammography screening given equal attention in scientific articles? A cross-sectional study. BMC Med. 2007;5:12. doi: 10.1186/1741-7015-5-12.
8. Marmot MG, Altman DG, Cameron DA, Dewar JA, Thompson SG, Wilcox M. The benefits and harms of breast cancer screening: an independent review. Br J Cancer. 2013;108(11):2205-2240. doi: 10.1038/bjc.2013.177.
9. Houssami N. Overdiagnosis of breast cancer in population screening: does it make breast screening worthless? Cancer Biol Med. 2017;14(1):1-8. doi: 10.20892/j.issn.2095-3941.2016.0050.
10. Hargraves IG, Montori VM, Brito JP, Kunneman M, Shaw K, LaVecchia C, Wilson M, Walker L, Thorsteinsdottir B. Purposeful SDM: A problem-based approach to caring for patients with shared decision making. Patient Educ Couns 2019102. 2019;102(10):1786-1792. doi: 10.1016/j.pec.2019.07.020. PMID: 31353170; PMCID: PMC6717012.
11. Barr PJ, Scholl I, Bravo P, Faber MJ, Elwyn G, McAllister M. Assessment of patient empowerment--a systematic review of measures. PLoS One. 2015 May 13;10(5):e0126553. doi: 10.1371/journal.pone.0126553. PMID: 25970618; PMCID: PMC4430483.
12. Elwyn G, Edwards A, Kinnersley P, Grol R. Shared decision making and the concept of equipoise: the competences of involving patients in healthcare choice. Br J Gen Pract. 2000;50:892-897. Available from: https://bjgp.org/content/bjgp/50/460/892.full.pdf.
13. Elwyn G, Frosch DL, Kobrin S. Implementing shared decision-making: consider all the consequences. Implement Sci. 2016;11:114. doi: 10.1186/s13012-016-0480-9. PMID: 27502770; PMCID: PMC4977650.
14. Walsh T, Barr PJ, Thompson R, Ozanne E, O'Neill C, Elwyn G. Undetermined impact of patient decision support interventions on healthcare costs and savings: systematic review. BMJ. 2014;348:g188. doi: 10.1136/bmj.g188. PMID: 24458654; PMCID: PMC3900320.
15. Katz SJ, Hawley S. The value of sharing treatment decision making with patients: expecting too much? JAMA. 2013;310(15):1559-1560. doi: 10.1001/jama.2013.278944. PMID: 24061082.
16. Oshima Lee E, Emanuel EJ. Shared decision making to improve care and reduce costs. N Engl J Med. 2013;368(1):6-8. doi: 10.1056/NEJMp1209500. PMID: 23281971.
17. Ryan M, Gerard K. Using discrete choice experiments to value health care programmes: current practice and future research reflections. Appl Health Econ Health Policy. 2003;2(1):55-64. PMID: 14619274.

For peer review only - http://bmjopen.bmj.com/site/about/guidelines.xhtml

18. Ryan M, Bate A, Eastmond CJ, Ludbrook A. Use of discrete choice experiments to elicit preferences. Qual Health Care. 2001;10;Suppl 1:i55-60. doi: 10.1136/qhc.0100055: 11533440; PMCID: PMC1765744.
19. Taguchi R, Yamazaki Y, Nakayama K. Eliciting preferences for mammography: using a discrete choice experiment approach. Nihon koshu eisei zasshi. 2010;57(2):83-94. PMID: 20415238.
20. Longo MF, Cohen DR, Hood K, Edwards A, Robling M, Elwyn G, Russell IT. Involving patients in primary care consultations: assessing preferences using discrete choice experiments. Br J Gen Pract. 2006 ;56(522):35-42. PMID: 16438813; PMCID: PMC1821413.
21. Norman R, Craig BM, Hansen P, Jonker MF, Rose J, Street DJ, Mulhern B. Issues in the design of discrete choice experiments. Patient. 2019;12(3):281-285. doi: 10.1007/s40271-018-0346-0. PMID: 30446958.
22. Elwyn G, Frosch D, Thomson R, Joseph-Williams N, Lloyd A, Kinnersley P, Cording E, Tomson D, Dodd C, Rollnick S, Edwards A, Barry M. Shared decision making: a model for clinical practice. J Gen Intern Med. 2012 Oct;27(10):1361-7. doi: 10.1007/s11606-012-2077-6. Epub 2012 May 23. PMID: 22618581; PMCID: PMC3445676.
23. Elwyn G, Durand MA, Song J, Aarts J, Barr PJ, Berger Z, Cochran N, Frosch D, Galasiński D, Gulbrandsen P, Han PKJ, Härter M, Kinnersley P, Lloyd A, Mishra M, Perestelo-Perez L, Scholl I, Tomori K, Trevena L, Witteman HO, Van der Weijden T. A three-talk model for shared decision making: multistage consultation process. BMJ. 2017;359:j4891. doi: 10.1136/bmj.j4891. PMID: 29109079; PMCID: PMC5683042.
24. Department of Health. Approval of public prices of the Catalan Health Service SLT/30/2013. Num 6323 Diari Oficial de la Generalitat de Catalunya [Aprobación de precios públicos del Servicio Catalán de Salud SLT/30/2013. Num 6323 Diari Oficial de la Generalitat de Catalunya]. 2013, 6323; 10695-10862. Available from: https://www.uch.cat/documents/preus-publics-catsalut.pdf
25. Laza-Vásquez C, Codern-Bové N, Cardona-Cardona À, Hernández-Leal MJ, Pérez-Lacasta MJ, Carles-Lavila M, Rué M; DECIDO group. Views of health professionals on risk-based breast cancer screening and its implementation in the Spanish National Health System: A qualitative discussion group study. PLoS One. 2022 Feb 4;17(2):e0263788. doi: 10.1371/journal.pone.0263788. PMID: 35120169; PMCID: PMC8815913.Reed Johnson F, Lancsar E, Marshall D, Kilambi V, Mühlbacher A, Regier DA, Bresnahan BW, Kanninen B, Bridges JF. Constructing experimental designs for discrete-choice experiments: report of the ISPOR Conjoint Analysis Experimental Design Good Research Practices Task Force. Value Health. 2013;16(1):3-13. doi: 10.1016/j.jval.2012.08.2223. PMID: 23337210
26. Reed Johnson F, Lancsar E, Marshall D, Kilambi V, Mühlbacher A, Regier DA, Bresnahan BW, Kanninen B, Bridges JF. Constructing experimental designs for discrete-choice experiments: report of the ISPOR Conjoint Analysis Experimental Design Good Research Practices Task Force. Value Health. 2013;16(1):3-13. doi: 10.1016/j.jval.2012.08.2223. PMID: 23337210.
27. Hernández-Leal MJ, Carles-Lavila M, Pérez-Lacasta M. La participación de los profesionales de la salud en la Toma de Decisiones Compartida en el cribado de cáncer de mama: manual de apoyo para implementación de la TDC. [The participation of health professionals in shared decision-making in breast cancer screening: A support manual

For peer review only - http://bmjopen.bmj.com/site/about/guidelines.xhtml

for the implementation of SDM]. Spain: María José Hernández editor. 2021. Available from: https://www.researchgate.net/publication/358277309_The_Participation_of_Health_Professionals_in_Shared_Decision-Making_on_Breast_Cancer_Screening
28. Ryan M, Bate A. Testing the assumptions of rationality, continuity and symmetry when applying discrete choice experiments in health care. Appl Econ Lett. 2001;8(1):59-63. doi: 10.1080/13504850150204112.
29. Mariel P, Hoyos D, Meyerhoff J, Czajkowski M, Dekker T, Glenk K, Jacobsen JB, Liebe U, Olsen SB, Sagebiel J, Thiene M. 2020. Environmental valuation with discrete choice experiments: guidance on design, implementation and data analysis. Springer;2020, pp 54-58. Available from: http://eprints.whiterose.ac.uk/168622/.
30. de Bekker-Grob EW, Donkers B, Jonker MF, Stolk EA. Sample size requirements for discrete-choice experiments in healthcare: a practical guide. Patient. 2015;8(5):373-384. doi: 10.1007/s40271-015-0118-z. PMID: 25726010; PMCID: PMC4575371.
31. McFadden D. Conditional logit analysis of qualitative choice behavior. 1973. Zarembka, P. Frontiers in econometrics. Academic Press: New York, pp 105-142.
32. Herrera C. Valoración y modelado de las preferencias en calidad de vida relacionadas con la salud de menores [Valuation and modeling of health related quality of life preferences of younger population]. 2020. Available from: https://riull.ull.es/xmlui/bitstream/handle/915/20075/Valoracion%20y%20modelado%20de%20las%20preferencias%20en%20calidad%20de%20vida%20relacionadas%20con%20la%20salud%20de%20menores..pdf?sequence=1.
33. Watson V, Becker F, de Bekker-Grob E. Discrete Choice Experiment Response Rates: A Meta-analysis. Health Econ. 2017 Jun;26(6):810-817. doi: 10.1002/hec.3354. Epub 2016 Apr 27. PMID: 27122445.
34. Hersch JK, Nickel BL, Ghanouni A, Jansen J, McCaffery KJ. Improving communication about cancer screening: moving towards informed decision making. Public Health Res Pract. 2017;27(2):2731728. doi: 10.17061/phrp2731728. PMID: 28765861.
35. Pérez-Lacasta MJ, Martínez-Alonso M, Garcia M, Sala M, Perestelo-Pérez L, Vidal C, et al. Effect of information about the benefits and harms of mammography on women's decision making: the InforMa randomised controlled trial. PLOS ONE. 2019;14(3):e0214057. doi: 10.1371/journal.pone.0214057.
36. Maschke A, Paasche-Orlow MK, Kressin NR, Schonberg MA, Battaglia TA, Gunn CM. Discussions of potential mammography benefits and harms among patients with limited health literacy and providers: 'oh, there are harms?' J Health Commun. 2020;25(12):951-961. doi: 10.1080/10810730.2020.1845256. PMID: 33455518; PMCID: PMC8062298.
37. Forbes LJ, Ramirez AJ, Expert group on Information about Breast Screening. Offering informed choice about breast screening. J Med Screen. 2014;21(4):194-200. doi: 10.1177/0969141314555350. PMID: 25312639.
38. Ballesteros-Peña S, Gavilán-Moral E. Contenido de los documentos informativos dirigidos a las mujeres sobre el cribado de cáncer de mama en España [Content of official addressed to women informative documents about breast cancer screening in Spain]. Rev Esp Salud Publica. 2018;92:e201810076. Spanish. PMID: 30361472.
39. Millaruelo J, Martín F. Toma de decisiones compartidas con el paciente [Shared decision-making with the patient]. Rev Clin Elect en Aten Pri. 2009. Available from.

For peer review only

Available from: https://ddd.uab.cat/pub/rceap/rceap_a2009m5n17/rceap_a2009m5n17a9.pdf
40. Hernández-Leal MJ, Pérez-Lacasta MJ, Feijoo-Cid M, Ramos-García V, Carles-Lavila M. Healthcare professionals' behaviour regarding the implementation of shared decision-making in screening programmes: A systematic review. Patient Educ Couns. 2021 Aug;104(8):1933-1944. doi: 10.1016/j.pec.2021.01.032. PMID: 33581968.
41. Hersch J, Jansen J, McCaffery K. Decision-making about mammographic screening: pursuing informed choice. Climacteric. 2018;21(3):209-213, doi: 10.1080/13697137.2017.1406912
42. Holmström I, Rosenqvist U. A change of the physicians understanding of the encounter parallels competence development. Patient Educ Couns. 2001;42(3):271-278. doi: 10.1016/s0738-3991(00)00132-4. PMID: 11164326.
43. Vargas I, Guerrero S, Hernández-Leal M, Campos R. La Confianza en la relación profesional de la salud y paciente [Confidence in the health professional and patient relation] Rev Cubana Salud Pública. 2020;26(3). Available from: http://www.revsaludpublica.sld.cu/index.php/spu/article/view/1575/1634.
44. Sala M, Domingo L, Rué M, Comas M, Torá-Rocamora I, Macià F, Castells X. Investigación en cribado de cáncer de mama: camino hacia estrategias personalizadas y decisiones compartidas [Research on breast cancer screening: toward personalized strategies and shared decision-making]. Revista de Senología y Patología Mamaria. 2014;27(4):176-182. doi: 10.1016/j.senol.2014.07.007.
45. Mathioudakis AG, Salakari M, Pylkkanen L, Saz-Parkinson Z, Bramesfeld A, Deandrea S, Lerda D, Neamtiu L, Pardo-Hernandez H, Solà I, Alonso-Coello P. Systematic review on women's values and preferences concerning breast cancer screening and diagnostic services. Psycho-Oncology. 2019;28(5):939-947. doi: 10.1002/pon.5041. PMID: 30812068; PMCID: PMC6594004.
46. Baena-Cañada JM, Rosado-Varela P, Expósito-Álvarez I, González-Guerrero M, Nieto-Vera J, Benítez-Rodríguez E. Women's perceptions of breast cancer screening. Spanish screening programme survey. Breast. 2014;23(6):883-888. doi: 10.1016/j.breast.2014.09.010. PMID: 25456103.
47. Toledo-Chávarri A, Rué M, Codern-Bové N, Carles-Lavila M, Perestelo-Pérez L, Pérez-Lacasta MJ, et al. A qualitative study on a decision aid for breast cancer screening: views from women and health professionals. Eur J Cancer Care (Engl). 2017;26(3):1-11. doi: 10.1111/ecc.12660.
48. Rainey L, van der Waal D, Donnelly LS, Southworth J, French DP, Evans DG, Broeders MJM. Women's health behaviour change after receiving breast cancer risk estimates with tailored screening and prevention recommendations. BMC Cancer. 2022;22(1):69. doi: 10.1186/s12885-022-09174-3. PMID: 35033023.
49. Esquivel-Sada D, Lévesque E, Hagan J, Knoppers BM, Simard J. Envisioning implementation of a personalized approach in breast cancer screening programs: stakeholder perspectives. Healthc Policy. 2019;15(2):39-54. doi: 10.12927/hcpol.2019.26072. PMID: 32077844; PMCID: PMC7020798.
50. Rimer BK, Briss PA, Zeller PK, Chan EC, Woolf SH. Informed decision making: what is its role in cancer screening? Cancer. 2004;101(5);Suppl:1214-1228. doi: 10.1002/cncr.20512. PMID: 15316908.
51. Caverly TJ, Hayward RA. Dealing with the lack of time for detailed shared decision-making in primary care: everyday shared decision-making. J Gen Intern Med.

For peer review only - http://bmjopen.bmj.com/site/about/guidelines.xhtml

2020;35(10):3045-3049. doi: 10.1007/s11606-020-06043-2; PMID: 32779137; PMCID: PMC7572954.
52. Stacey D, Légaré F, Lewis K, Barry MJ, Bennett CL, Eden KB, Holmes-Rovner M, Llewellyn-Thomas H, Lyddiatt A, Thomson R, Trevena L. Decision aids for people facing health treatment or screening decisions. Cochrane Database Syst Rev. 2017;4(4):CD001431. doi: 10.1002/14651858.CD001431.pub5. PMID: 28402085; PMCID: PMC6478132.
53. Hernández-Leal M, Montori V. 2021. Discussion groups for decision-making on breast cancer screening [research proyect]. Mayo Clinic.
54. Légaré F, Ratté S, Gravel K, Graham ID. Barriers and facilitators to implementing shared decision-making in clinical practice: update of a systematic review of health professionals' perceptions. Patient Educ Couns. 2008;73(3):526-535. doi: 10.1016/j.pec.2008.07.018.
55. Légaré F, Witteman HO. Shared decision making: examining key elements and barriers to adoption into routine clinical practice. Health Aff (Millwood). 2013;32(2):276-284. doi: 10.1377/hlthaff.2012.1078.
56. Johnson P, Bancroft T, Barron R, Legg J, Li X, Watson H, Naeim A, Watkins A, Marshall DA. Discrete choice experiment to estimate breast cancer patients' preferences and willingness to pay for prophylactic granulocyte colony-stimulating factors. Value Health. 2014;17(4):380-389. doi: 10.1016/j.jval.2014.01.002. PMID: 24968998.
57. Wilson LS, Blonquist TM, Hong F, Halpenny B, Wolpin S, Chang P, Filson CP, Master VA, Sanda MG, Chien GW, Jones RA, Krupski TL, Berry DL. Assigning value to preparation for prostate cancer decision making: a willingness to pay analysis. BMC Med Inform Decis Mak. 2019;19(1):6. doi: 10.1186/s12911-018-0725-4. PMID: 30626400; PMCID: PMC6327504.
58. Brito Fernandes Ó, Péntek M, Kringos D, Klazinga N, Gulácsi L, Baji P. Eliciting preferences for outpatient care experiences in Hungary: A discrete choice experiment with a national representative sample. PLoS One. 2020;15(7):e0235165. doi: 10.1371/journal.pone.0235165. PMID: 32735588; PMCID: PMC7394384.
59. Puig-Junoy J, Pinto-Prades JL, Ortún-Rubio V. El análisis coste-beneficio en sanidad [Cost-benefit analysis in health care]. Aten Primaria. 2001;27(6):422-7. Spanish. doi: 10.1016/s0212-6567(01)78825-7. PMID: 11334581; PMCID: PMC7677961.
60. Mansfield C, Tangka FK, Ekwueme DU, Smith JL, Guy GP Jr, Li C, Hauber AB. Stated preference for cancer screening: A systematic review of the literature, 1990-2013. Prev Chronic Dis. 2016;13:E27. doi: 10.5888/pcd13.150433. PMID: 26916898; PMCID: PMC4768876.

11. FIGURE LEGENDS

Figure 1. Examples of a choice task for conventional (first version) and narrative (final version)

Figure 2. Net follow-up of survey responses

Figure 3. Flowchart of participants

For peer review only - http://bmjopen.bmj.com/site/about/guidelines.xhtml

Figure 1. Examples of a choice task for conventional (first version) and narrative (final version)

Conventional choice task (first version)

**Characteristics**	**Option A**	**Option B**
Information	I was informed by leaflet	I was informed by a health professional
Communication	I discuss mammography with a healthcare professional	The health system schedules my mammography
Make a decision	I make the decision of whether to have a mammogram	I make the decision of whether to have a mammogram
Choose one option		

Pre-test →

Narrative choice task (final version)

**Option A**	**Option B**
I receive **information about the benefits and risks in a leaflet**, and I also **talk with a healthcare professional** about my fears or beliefs about mammography. Finally, **I make the decision** to attend -or not- to mammography.	I am **informed by a health professional** about the benefits and risks; my **mammography is scheduled by the health system**, so I do not manifest my fears, beliefs or preferences about the screening to a health professional. Finally, **I make the decision** to attend -or not- to mammography.

Figure 1. Examples of a choice task for conventional (first version) and narrative (final version)

254x97mm (144 x 144 DPI)

Figure 2. Net follow-up of survey responses

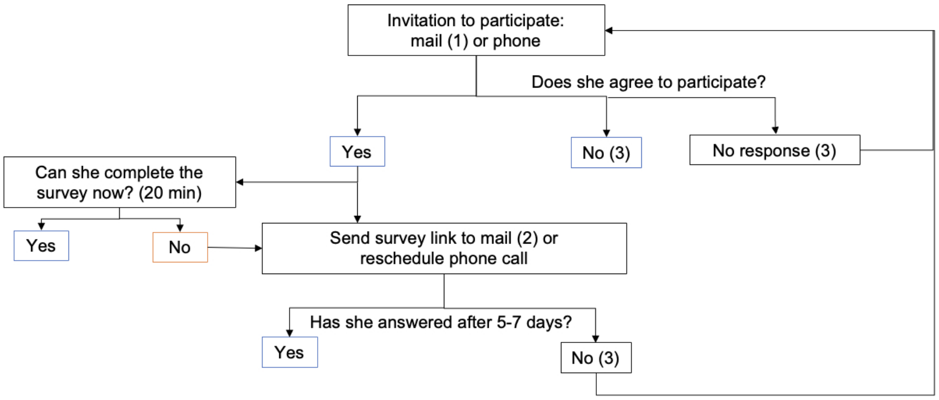

1) Attach ethics committee approval
2) Spanish and Catalan survey link
3) If the participants do not respond to the invitation or survey after three attempts, delete them from the database

Figure 2. Net follow-up of survey responses

226x138mm (144 x 144 DPI)

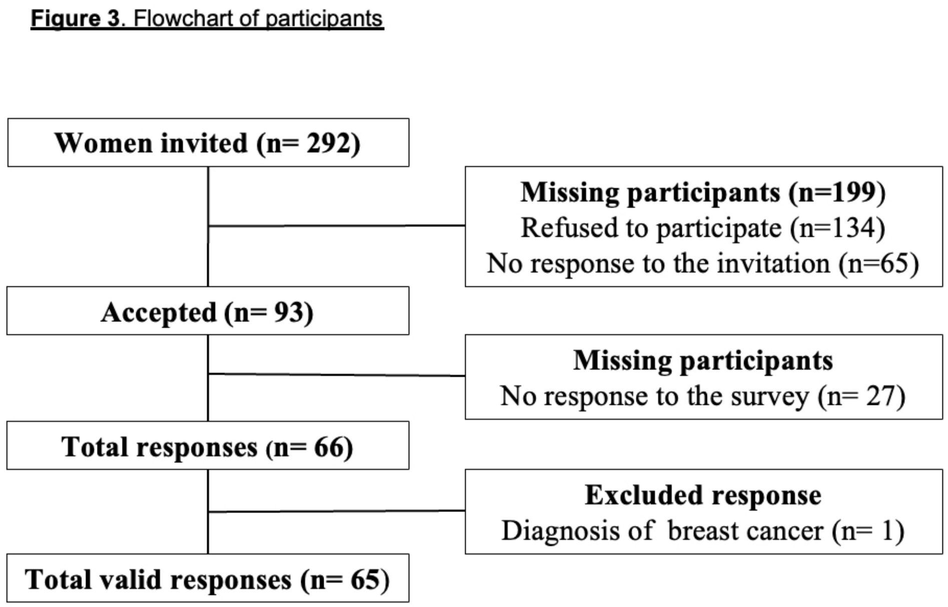

Figure 3. Flowchart of participants

168x107mm (144 x 144 DPI)

"Women's Preferences for Breast Cancer Screening"

We appreciate your collaboration in the ProShare research project "*The collaboration of healthcare professionals to include Shared Decision Making in the breast cancer screening program*" (P18/00773), led by Dr. Misericòrdia Carles. This project is supported and funded by the Carlos III Institute of Health and co-financed by the European Regional Development Fund (ERDF) "A way of making Europe" y "European Union's Horizon 2020 research and innovation program under the Marie Skłodowska-Curie grant agreement" No. 713679 and from the Universitat Rovira i Virgili (URV).

The objective of your participation is to identify women's preferences and determine the cost-benefit of a new care model for the breast cancer screening program. So, we present you, in two hypothetical options and you must choose only one of them according to your preferences. Consider that there are no good or bad answers.

Your answers will be confidential and will only be used for research purposes. Answer the following questions truthfully, considering that your identity will never be exposed.

ProShare
SHARED DECISION MAKING IN HEALTH
Shared Decision Making in Health

1. Socio-demographic characteristics
a. Name: ___________

For peer review only

b. Age (years):______________

c. Birthplace (mark with an X)

	Spain
	Other country

d. Civil status (mark with an X)

	Single
	With partner

e. Education level (mark with an X)

	Primary
	Secondary
	University

f. Main occupation (mark with an X)

	Paid work
	Household chores
	Unemployed

g. Family history of breast cancer (mark with an X)

	Yes
	No
	Unknown

h. Do you have your mammogram regularly? (mark with an X)

	Yes
	No

For peer review only

2. Experimental design

The next 12 questions will be asked in consultation on three characteristics of the breast cancer screening program:

1. **How do I receive the information?** You will find two possibilities; in one of them you are informed about the benefits and risks of mammography through a **leaflet** or by **health professional**.

2. Dialogue about my preferences? You'll find two possibilities; in one, you **meet with a healthcare professional** to discuss your preferences, fears, or concerns about mammography. And on the other hand, **the health system** simply programs its mammography according to its age.

3. Who makes the decision? You will find three options about who makes the decision for attended -or no- to mammography: the **health professional**, **the woman** or **jointly**. Independent if later this is concrete the mammography

In each question you must choose the option you prefer. Each option includes the three features above.

For example:
Question 0: Mark with an "X" the option you prefer

Option A	Option B
I am informed by a **healthcare professional** about the benefits and risks, **I also express my fears**, beliefs, or preferences of mammography. Finally, **I myself made the decision** attend -or not- mammography.	I am informed by a **healthcare professional** about the benefits and risks, **I also express my fears**, beliefs or preferences of mammography. Finally, **we jointly made the decision** attend -or not- to mammography.

We are aware that in some questions the options may be very similar to you, yet there is always some different characteristics There are no correct or incorrect answers, you should select only based on your personal appreciation.

SURVEY

1: Mark with an "X" the option you prefer

Option A	Option B

**The healthcare professionals inform** me about the benefits and risks; **I also tell them about my fears**, beliefs, or preferences for mammography. Finally, **the healthcare professional makes the decision** attend -or not- to the mammography.	**The healthcare professionals inform me** about the benefits and risks; **I also tell them about my fears**, beliefs, or preferences for mammography. Finally, **I myself made the decision** attend -or not- to the mammography.

2.Mark with an "X" the option you prefer

Option A	Option B
I receive information about the benefits and risks through a **leaflet**; **I also share with a healthcare professional my fears** or beliefs about mammography. Finally, **we jointly made the decision** attend -or not- to the mammography.	**I am informed by a healthcare professional** about the benefits and risks; **I also express my fears, beliefs,** or preferences of mammography. Finally, **I myself made the decision** attend -or not- to the mammography.

3. Mark with an "X" the option you prefer

Option A	Option B
I receive information about the benefits and risks through **a leaflet**; **I also tell a healthcare professional my fears** or beliefs about mammography. Finally, **the professional makes the decision** attend -or not- to the mammography.	**The healthcare professionals inform** me about the benefits and risks; **I also tell them about my fears**, beliefs, or preferences for mammography. Finally, **I myself made the decision** attend -or not- to the mammography.

4. Mark with an "X" the option you prefer

Option A	Option B
**I am informed by a healthcare professional** about the benefits and risks, but **without the possibility of expressing my fears** or beliefs about mammography as it is defined by the health program. Finally, **I myself made the decision** attend -or not- to the mammography.	**The healthcare professional informs me** about the benefits and risks**; I also tell them about my fears**, beliefs, or preferences for mammography. Finally, **the healthcare professional makes the decision** attend -or not- to the mammography.

5. Mark with an "X" the option you prefer

Option A	Option B
I receive information about the benefits and risks through **a leaflet**; I also **tell a**	**The healthcare professional informs me** about the benefits and risks; **I also**

For peer review only

**healthcare professional my fears** or beliefs about mammography. Finally, **we jointly made the decision** attend -or not- to the mammography.	**tell them about my fears**, beliefs, or preferences for mammography. Finally, the **healthcare professional makes the decision** attend -or not- to the mammography.

6. Mark with an "X" the option you prefer

Option A	Option B
I receive information about the benefits and risks through **a leaflet**; **I also tell a healthcare professional my fears** or beliefs about mammography. Finally, **I myself made the decision** attend -or not- to the mammography.	**The healthcare professional informs me** about the benefits and risks; **I also tell them about my fears**, beliefs, or preferences for mammography. Finally, **the healthcare professional makes the decision** attend -or not- to the mammography.

7. Mark with an "X" the option you prefer

Option A	Option B
I receive information about the benefits and risks through **a leaflet**; **my mammography programs the health system**, so I do not manifest my fears, beliefs, or preferences about mammography to a health professional. Finally, **I myself made the decision** attend -or not- to the mammography.	**The healthcare professional informs me** about the benefits and risks; **I also tell them about my fears**, beliefs, or preferences for mammography. Finally, the **healthcare professional makes the decision** attend -or not- to the mammography.

8. Mark with an "X" the option you prefer

Option A	Option B
I receive information about the benefits and risks through **a leaflet**; **I also tell a healthcare professional my fears** or beliefs about mammography. Finally, **we jointly made the decision** attend -or not- to the mammography.	**I am informed by a health professional** about the benefits and risks; **my mammography programs the health system**, so I do not manifest my fears, beliefs, or preferences about mammography to a health professional. Finally, **I myself made the decision** attend -or not- to the mammography.

9. Mark with an "X" the option you prefer

Option A	Option B
I receive information about the benefits and risks in a **leaflet; I also share my fears** or beliefs about mammography with a health professional. Finally, **I myself**	**I am informed by a health professional** about the benefits and risks; my mammography programs the health system, **so I do not manifest my fears**, beliefs or preferences about

**made the decision** attend -or not- to the mammography.	mammography with a health professional. Finally, **I myself made the decision** attend -or not- to the mammography.

10. Mark with an "X" the option you prefer

Option A	Option B
I receive information about the benefits and risks in a **leaflet**; **I also share my fears** or beliefs about mammography with a health professional. Finally, **the professional makes the decision** attend -or not- to the mammography.	**I am informed by a health professional** about the benefits and risks; however, **I do not manifest my fears**, beliefs or preferences about mammography as it programs the health system. Finally, **I myself made the decision** attend -or not- to the mammography

11. Mark with an "X" the option you prefer

Option A	Option B
I receive information about the benefits and risks in a **leaflet**; **I also share my fears** or beliefs about mammography with a health professional. Finally, **the professional makes the decision** attend -or not- to the mammography	I receive information about the benefits and risks in a **leaflet**; **I also share my fear**s or beliefs about mammography with a health professional. Finally, **I myself made the decision** attend -or not- to the mammography

12. Mark with an "X" the option you prefer

Option A	Option B
I receive information about the benefits and risks a **leaflet**; **my mammography programs the health system**, so I do not manifest my fears, beliefs or preferences about mammography with a health professional. Finally, **I myself made the decision** attend -or not- to the mammography	I receive information about the benefits and risks in a **leaflet**; **I also share my fears** or beliefs about mammography with a health professional. Finally**, the professional makes the decision** attend -or not- to the mammography

3. Cost of health

The breast cancer screening is currently not paid to women, as it is covered by the public health service. Regardless of the current cost, if you had the option of attending an appointment with a healthcare professional to inform you about the benefits and risks of mammography, discuss with him/her your preferences and concerns and jointly make the decision to attend -or not to mammography **How much money would you be willing to pay?**

	0 euros

For peer review only

	10 euros
	20 euros
	30 euros
	40 euros
	60 euros or more

Thank you so much for the time you took to answer this survey to improve women's care in breast cancer screening.

ProShare

For peer review only

Women's Preference to Apply Shared Decision-Making in Breast Cancer Screening: A Discrete Choice Experiment

STROBE Statement—Checklist of items that should be included in reports of ***cross-sectional studies***

	Item No	Recommendation	Page No
**Title and abstract**	1	(*a*) Indicate the study's design with a commonly used term in the title or the abstract	1
		(*b*) Provide in the abstract an informative and balanced summary of what was done and what was found	2
**Introduction**			
Background/rationale	2	Explain the scientific background and rationale for the investigation being reported	3
Objectives	3	State specific objectives, including any prespecified hypotheses	3
**Methods**			
Study design	4	Present key elements of study design early in the paper	4
Setting	5	Describe the setting, locations, and relevant dates, including periods of recruitment, exposure, follow-up, and data collection	6
Participants	6	(*a*) Give the eligibility criteria, and the sources and methods of selection of participants	6
Variables	7	Clearly define all outcomes, exposures, predictors, potential confounders, and effect modifiers. Give diagnostic criteria, if applicable	5
Data sources/ measurement	8*	For each variable of interest, give sources of data and details of methods of assessment (measurement). Describe comparability of assessment methods if there is more than one group	*5*
Bias	9	Describe any efforts to address potential sources of bias	5
Study size	10	Explain how the study size was arrived at	6
Quantitative variables	11	Explain how quantitative variables were handled in the analyses. If applicable, describe which groupings were chosen and why	6
Statistical methods	12	(*a*) Describe all statistical methods, including those used to control for confounding	6
		(*b*) Describe any methods used to examine subgroups and interactions	6
		(*c*) Explain how missing data were addressed	NA
		(*d*) If applicable, describe analytical methods taking account of sampling strategy	6
		(*e*) Describe any sensitivity analyses	6
**Results**			
Participants	13*	(a) Report numbers of individuals at each stage of study—eg numbers potentially eligible, examined for eligibility, confirmed eligible, included in the study, completing follow-up, and analysed	7
		(b) Give reasons for non-participation at each stage	7
		(c) Consider use of a flow diagram	7
Descriptive data	14*	(a) Give characteristics of study participants (eg demographic, clinical, social) and information on exposures and potential confounders	7-8
		(b) Indicate number of participants with missing data for each variable of interest	7
Outcome data	15*	Report numbers of outcome events or summary measures	8

For peer review only - http://bmjopen.bmj.com/site/about/guidelines.xhtml

Main results	16	(*a*) Give unadjusted estimates and, if applicable, confounder-adjusted estimates and their precision (eg, 95% confidence interval). Make clear which confounders were adjusted for and why they were included	8-9
		(*b*) Report category boundaries when continuous variables were categorized	
		(*c*) If relevant, consider translating estimates of relative risk into absolute risk for a meaningful time period	NA
Other analyses	17	Report other analyses done—eg analyses of subgroups and interactions, and sensitivity analyses	10-12
**Discussion**			
Key results	18	Summarise key results with reference to study objectives	10
Limitations	19	Discuss limitations of the study, taking into account sources of potential bias or imprecision. Discuss both direction and magnitude of any potential bias	15-16
Interpretation	20	Give a cautious overall interpretation of results considering objectives, limitations, multiplicity of analyses, results from similar studies, and other relevant evidence	12-15
Generalisability	21	Discuss the generalisability (external validity) of the study results	15-16
**Other information**			
Funding	22	Give the source of funding and the role of the funders for the present study and, if applicable, for the original study on which the present article is based	16

*Give information separately for exposed and unexposed groups.

Note: An Explanation and Elaboration article discusses each checklist item and gives methodological background and published examples of transparent reporting. The STROBE checklist is best used in conjunction with this article (freely available on the Web sites of PLoS Medicine at http://www.plosmedicine.org/, Annals of Internal Medicine at http://www.annals.org/, and Epidemiology at http://www.epidem.com/). Information on the STROBE Initiative is available at www.strobe-statement.org.

For peer review only
